# Radotinib inhibits multiple myeloma cell proliferation via suppression of STAT3 signaling

**DOI:** 10.1371/journal.pone.0265958

**Published:** 2022-05-03

**Authors:** Sook-Kyoung Heo, Eui-Kyu Noh, Hye Jin Seo, Yoo Jin Lee, SuJin Koh, Young Joo Min, Yunsuk Choi, Jae-Cheol Jo

**Affiliations:** 1 Biomedical Research Center, Ulsan University Hospital, University of Ulsan College of Medicine, Ulsan, Republic of Korea; 2 Department of Hematology and Oncology, Ulsan University Hospital, University of Ulsan College of Medicine, Ulsan, Republic of Korea; UTHSC: The University of Texas Health Science Center at Houston, UNITED STATES

## Abstract

Multiple myeloma (MM) is a hematological cancer causing from accumulated abnormal plasma cells. STAT3 overexpression in MM appears to be mediated by a variety of factors, and it may be associated with an adverse prognosis and play a role in microenvironment-dependent treatment resistance. Unfortunately, MM remains an incurable disease, as relapse is very common. Therefore, there is urgent need to develop new treatment options for MM. Radotinib is a novel anti-cancer drug, currently approved in South Korea for the treatment of chronic myeloid leukemia patients. It is an oral, multitargeted inhibitor of receptor tyrosine kinases, including BCR-ABL, c-KIT, PDGFR, and Src family kinases. However, little is known about the effects of radotinib on multiple myeloma cells. However, little is known about the effects of radotinib on multiple myeloma cells. But even tinip almost not known about the impact of multiple myeloma cells. Moreover, nothing is known about how it affects STAT3 and JAK2. In this study, we analyzed the effect of radotinib on multiple myeloma cells. Herein, Moreover, nothing is known about how it. Moreover, not all is known about how the affects STAT3 and JAK2. We investigated the effect of radotinib on the STAT3 signaling pathway in MM cells, including several MM cell lines and mouse models. So we investigated the effect of radotinib on MM cells, including several MM cell lines and mouse models. Interestingly, radotinib induced apoptosis, and inhibited cell proliferation in MM cells including RPMI-8226, MM.1S, U266B1, and IM-9 cells. Moreover, radotinib treatment significantly increased the number Annexin V-positive cells and G0/G1-phase cells. In addition, radotinib treatment in various MM cells strongly suppressed the activity and expression of STAT3 and JAK2 proteins. We also observed that diverse proteins related to the STAT3 signaling pathway, including c-Myc, Bcl-xL, Mcl-1, cyclin D1 and cyclin D3, were powerfully inhibited by radotinib treatment in MM cells. Furthermore, radotinib significantly suppressed MM cell growth in a xenograft animal model using IM-9 cells. In conclusion, radotinib may play an important role as a candidate agent for MM treatment.

## Introduction

Multiple myeloma (MM) is a hematological cancer resulting from accumulated abnormal plasma cells [1]. It is characterized by the proliferation and accumulation of malignant plasma cells in the bone marrow, and is generally associated with a monoclonal protein [2]. Recently, there has been a dramatic increase in the number of treatment options available for the treatment of MM. In particular, the advents of immunomodulating agents, proteasome inhibitors, and monoclonal antibodies have led to significant improvements in the survival of patients with MM [3,4]. Unfortunately, MM remains still an incurable disease, as relapse is very common. Therefore, there is a crucial need to develop novel treatment options to cure MM.

Generally, signal transducer and activator of transcription 3 (STAT3), a member of the STAT protein family, can be phosphorylated by receptor-associated Janus kinases (JAKs) in response to stimulation by cytokines and growth factors [5,6]. Constitutive and sustained activation of STAT3 has been observed in many human solid and hematological cancer cells, including lung, breast, colon, leukemia, multiple myeloma, and lymphoma [7,8]. Moreover, aberrant activation of STAT3 is frequently observed in MM, and can upregulate the expression of several genes involved in proliferation, survival, and metastasis. In addition, activation of STAT3 has been found to be associated with shorter survival of the patients with MM [9]. A number of proliferation, pro-survival and anti-apoptotic genes are regulated by activation of STAT3 at the transcription level including Bcl-xL, Mcl-1, c-Myc, cyclin D1, and cyclin D3 [10–13]. For this reason, many researchers are participating with great interest in the research that STAT3 can be a novel molecular biological target in many cancers, including multiple myeloma [10,14,15].

Radotinib is an oral, multitargeted inhibitor of receptor tyrosine kinases, including BCR-ABL [16], c-Abl [17,18], and c-KIT [19,20]. It was approved as a second-line treatment for chronic myeloid leukemia (CML) patients in South Korea [21]. The structure of radotinib is very similar to imatinib and nilotinib [16]. It has recently been shown to have beneficial neuroprotective effects in a mouse model of Parkinson’s disease. Briefly, radotinib prevented dopaminergic neuron loss, neuritis, and behavioral defects through inhibition of c-Abl activation [17]. Furthermore, radotinib induced solid cancer cell death via activation of natural killer cell cytotoxicity [22]. In our previous study, radotinib induced apoptosis in various acute myeloid leukemia (AML) cells [23–25]. In particular, targeting c-KIT by radotinib promoted cell death in c-KIT-positive AML cells, and this mechanism was similar to that of dasatinib in AML cells [19,20,25]. Additionally, radotinib enhanced cytarabine-induced AML cell death *in vitro* and *in vivo* [26]. Furthermore, radotinib induces apoptosis in MM cells via mitochondrial‑dependent pathway [18]. However, little is known about the effects of radotinib on multiple myeloma cells. sSpecifically, nothing is known about how it affects STAT3 and JAK2. Herein, Moreover, nothing is known about how it affects STAT3 and JAK2.

Moreover, not all is known about how the affects STAT3 and JAK2.

We investigated the effect of radotinib on the STAT3 and its downstream signaling pathway in MM cells, including several MM cell lines and mouse models. So we investigated the effect of radotinib on MM cells, including several MM cell lines and mouse models.

However, little is known about the effects of radotinib on multiple myeloma cells.

But even tinip almost not known about the impact of multiple myeloma cells.

So we investigated the effects of radotinib in MM cells, including several MM cell lines and mouse models.

## Materials and methods

### Reagents

Radotinib was a generous gift from Ilyang Pharmaceutical Co., Ltd., (Seoul, South Korea) and its purity was 99.9% based on HPLC analysis [24]. All reagents were obtained from Sigma-Aldrich (St. Louis, MO, USA), unless otherwise indicated. The CellTiter 96 AQueous One Solution Cell Proliferation Assay was purchased from Promega (Madison, WI, USA). Apoptosis Detection Kit I was obtained from BD Bioscience (San Diego, CA, USA). The antibodies used in western blot analysis, anti-β-actin were purchased from Santa Cruz Biotechnology (Santa Cruz, CA, USA), while the rest were purchased from Cell Signaling Technology (Beverly, MA, USA). TUNEL Assay Kit was obtained from Abcam (San Diego, CA, USA).

### Ethics approval

All procedures involving animals were in accordance with the Laboratory Animals Welfare Act, the Guide for the Care and Use of Laboratory Animals, and the Guidelines and Policies for Rodent Experimentation provided by the Institutional Animal Care and Use Committee of the Ulsan University of Korea. This study protocol was approved by the institutional review board of the Ulsan University of Korea (Approval No. 0118–02).

### Cell culture

The RPMI-8226, MM.1S, U266B1, and IM-9 cells were obtained from the American Type Culture Collection (ATCC, Manassas, VA, USA). These cells were grown as suspension in RPMI 1640 medium supplemented with 10% heat-inactivated FBS (or 15% FBS for the U266B1 cell line) and 1% penicillin-streptomycin in a 5% CO_2_ humidified atmosphere at 37°C.

### Cell viability assay

Cell viability was measured by the CellTiter 96 AQueous One Solution Cell Proliferation Assay (Promega, Madison, WI, USA), according to the manufacturer’s protocol.

### Cell proliferation assay (BrdU assay)

Under the same culture conditions above, RPMI-8226, MM.1S, U266B1, and IM-9 cells were incubated with 0, 10, 50, and 100 μM of radotinib for 72 h at 37°C. Cell proliferation was measured by BrdU (5′-bromo-2-deoxyuridine) enzyme-linked immunosorbent assay (Cell Proliferation ELISA, BrdU; Roche Diagnostics), according to the manufacturer’s instructions.

### Flow cytometric analysis

Detection of Annexin V positive cells and cell cycle analysis were performed as previously described [18,25].

### Western blotting analysis and immunoprecipitation

Western blotting analysis and immunoprecipitation were conducted under the same conditions as above, and were performed as previously described [20,23]. The blots were developed using the ChemiDoc Touch Imaging System, and analyzed with the Image Lab Software, Version 6.0.1, (Bio‑Rad Laboratories, CA, USA).

### Transfection of STAT3 siRNA

SiRNA for STAT3 (cat No. E-003544-00-50) and cyclophilin B control siRNA (cat No. D-001910-01-50) were purchased from Dharmacon. U266B1 cells were transfected with siRNA using the Accell SYSTEM (Dharmacon), cultured for 48 h, and then used to measure the diverse proteins by western blotting. To examine for efficiency of transfection, we used Accell Green Non-targeting siRNA. Efficiency of transfection was over the 95% in the U266B1 cells.

### Mice

Specific-pathogen-free five-week-old athymic nude male mice were purchased from OrientBio (Seongnam, Korea), and kept in a clean environment of the Ulsan University of Korea (Korea, Ulsan). All mice were housed in a clean environment with standard conditions performed as previously described [26], and handled in according with the Institutional Animal Care and Use Committee of the University of Ulsan (Ulsan, Korea, Approval No. 0118–02).

### Xenograft animal model using IM-9 cells

The athymic nude male mice were implanted with IM-9 cells (3 × 10^7^ cells/mouse with 200 μl of matrigel) in the right flank region. Radotinib treatment was performed intraperitoneally every morning for five days except weekends. The mice were sacrificed on days 30–32 following tumor cell implantation. The tumors were excised and weighted, and each tumor tissue homogenized for the preparation of cell samples for several analyses.

### TUNEL assay

TUNEL Assay Kit according to the manufacturer’s instructions (Abcam, Cambridge, United Kingdom). The samples were analyzed with a Fluorescence microscope (Olympus, NY, USA).

### Immunofluorescence

Under the same conditions above, performed as previously described [26].

### Statistics

Experimental results were analyzed including calculation of mean and standard error (mean ± SEM) using ImageJ (version 1.5, LOCI, Madison, WI, USA) and GraphPad Prism 7.0 (GraphPad Software, La Jolla, CA, USA). Differences among multiple means were assessed by one-way ANOVA or two-way ANOVA followed by Tukey’s test or by Bonferroni’s multiple comparisons test as appropriate. Probabilities of *P* < 0.05 were considered statistically significant.

## Results

### Radotinib induces apoptosis, and inhibits cell proliferation in multiple myeloma cells

**Fig 1:**
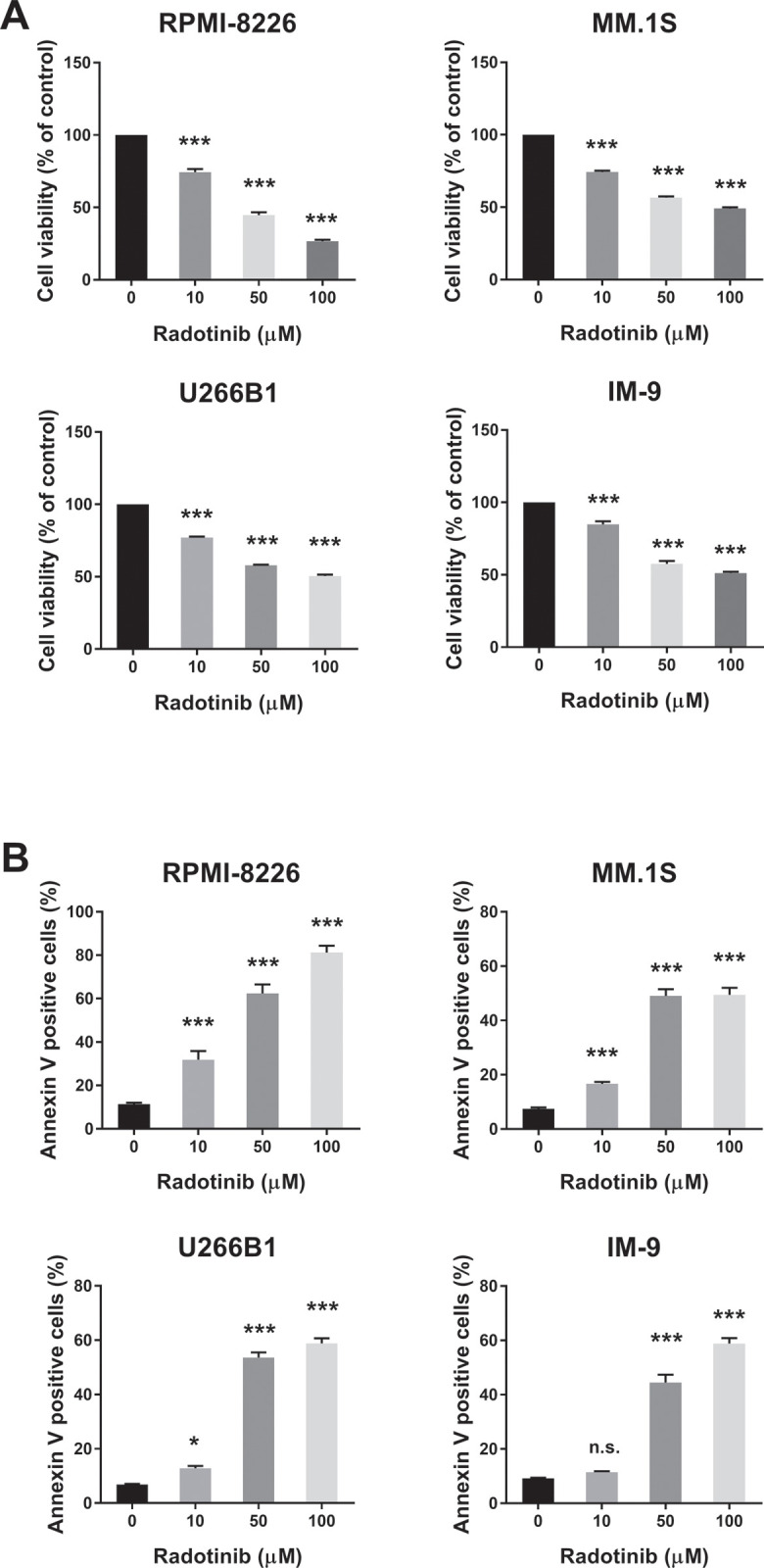
Radotinib induces cell death in diverse multiple myeloma cells. (A) Effects of radotinib on the viability of MM cells. Cells were incubated with 0, 10, 50, and 100 μM radotinib for 72 h in RPMI-8226, MM.1S, U266B1, and IM-9 cells. (B) Radotinib induces apoptosis in MM cells. The cells were also collected and treated under the same conditions described above. Cells were stained with Annexin V, followed by flow cytometry analysis. Data are presented mean ± SEM.; *, *P* < 0.05; and ***, *P* < 0.001. *Significantly different from control cells. n.s. no significance.

**Fig 2:**
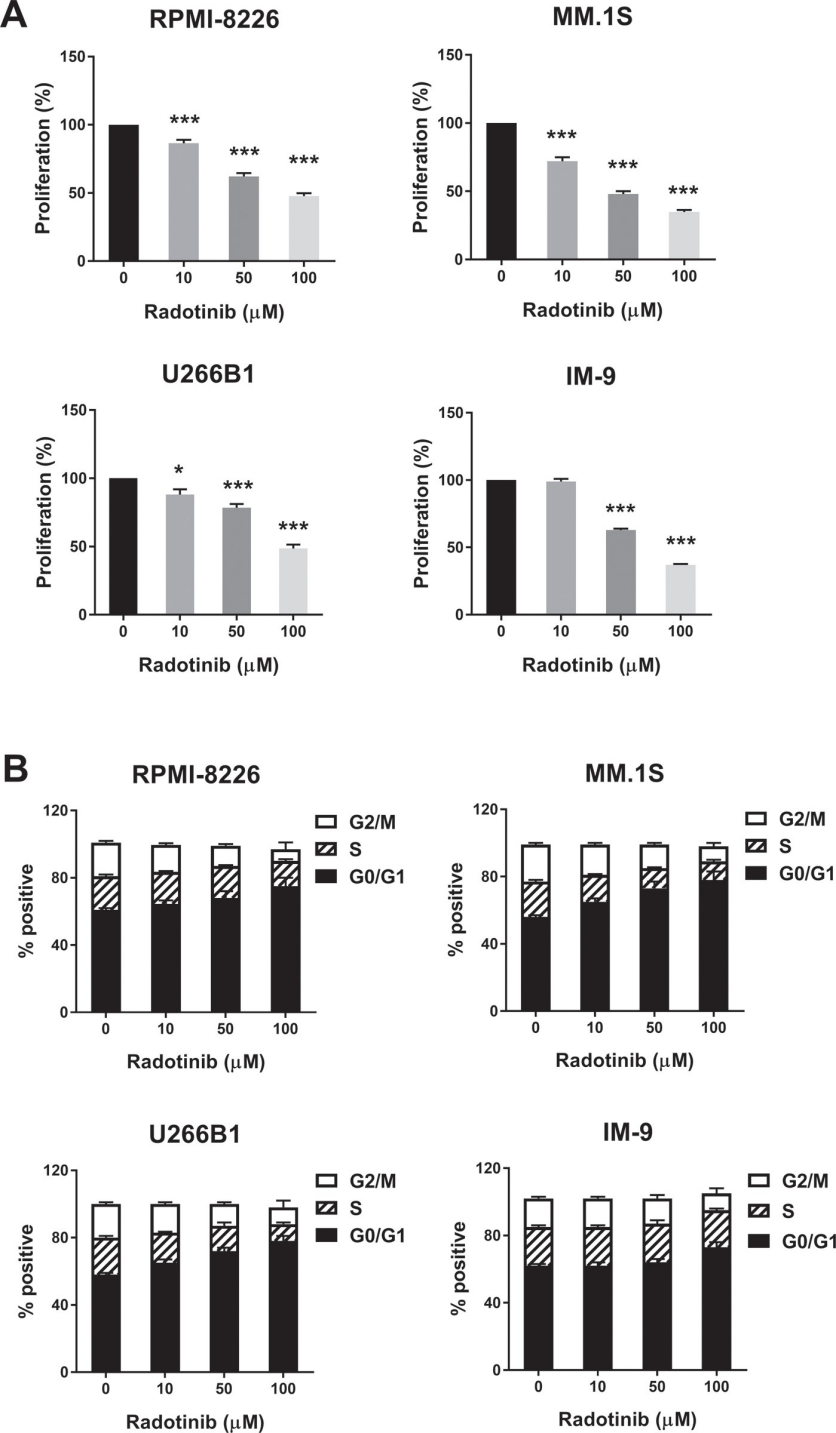
Radotinib inhibits MM cell proliferation, while augments G0/G1 phase cell cycle arrest in the four types of MM cells. (A) Cell proliferation at 72 h after radotinib treatment. (B) Cell cycle distribution at 72 h after radotinib treatment. Cell proliferation and cell cycle distribution were measured as described in Methods The cells were also collected and treated under the same conditions described above. Data are presented mean ± SEM.; *, *P* < 0.05; and ***, *P* < 0.001. *Significantly different from control cells.

To examine the effect of radotinib on the growth of MM cells, RPMI-8226, MM.1S, U266B1, and IM-9 cells were treated with 0, 1, 10, 50, and 100 μM radotinib for 72 h and cell viability was measured by the CellTiter 96 solution. Radotinib induced a significant cytotoxic effect on the four types of MM cell lines in a dose-dependent manner (Fig 1A; IC_50_ in 72hr, RPMI-8226: 10 μM, MM.1S: 100 μM, U266B1: 100 μM and IM-9: 100 μM). These results indicate that radotinib had cytotoxic effects on multiple myeloma cells. Next, we examined the ability of radotinib to induce apoptosis in MM cell lines. The cells were also cultured and treated under the same conditions as described above. Cells were stained with Annexin V-FITC, followed by flow cytometric analysis. As shown in Fig 1B, radotinib increased the number of apoptotic cells in a dose-dependent manner in RPMI-8226, MM.1S, U266B1, and IM-9 cells. Therefore, these results suggest that radotinib induces apoptosis in MM cells (Fig 1). The MM cell proliferation was assessed by BrdU incorporation. As shown in Fig 2A and 2B, radotinib strongly suppressed MM cell proliferation, and accelerated G0/G1-phase cell cycle arrest in RPMI-8226, MM.1S, U266B1, and IM-9 cells. These results indicate that radotinib induces cell death and inhibits cell proliferation in MM cells.

### Radotinib strongly inhibits the activity and expression of STAT3 and JAK2 in various MM cells

**Fig 3:**
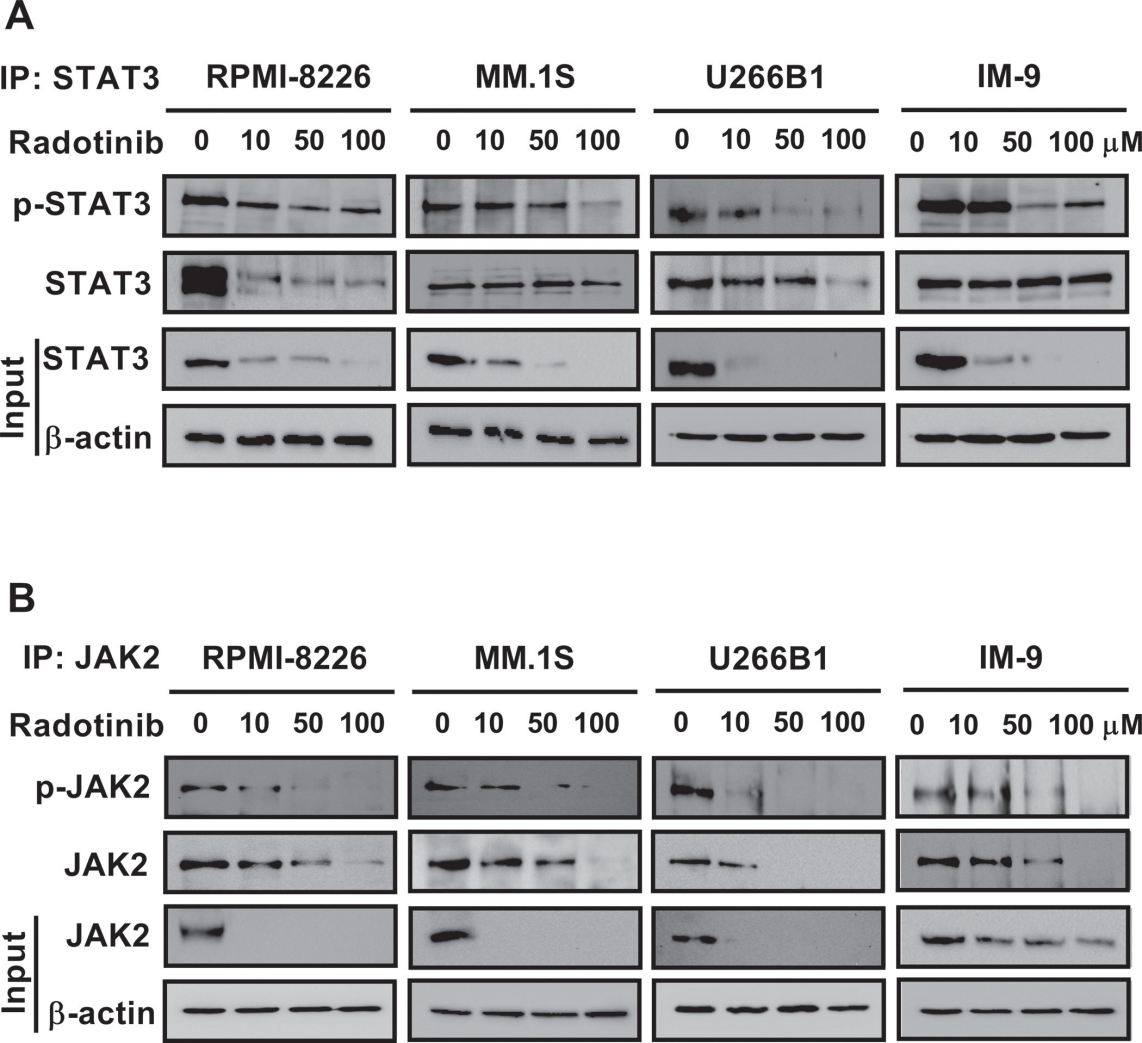
Radotinib strongly inhibits the activity and expression of STAT3 and JAK2 in various MM cells. MM cells were incubated with 0, 10, 50, and 100 μM radotinib for 72 h. (A) Effect of radotinib on the activity and expression of STAT3. (B) Effect of radotinib on the activity and expression of JAK2. The membrane was stripped and reprobed with anti-β-actin mAb to confirm equal loading.

**Fig 4:**
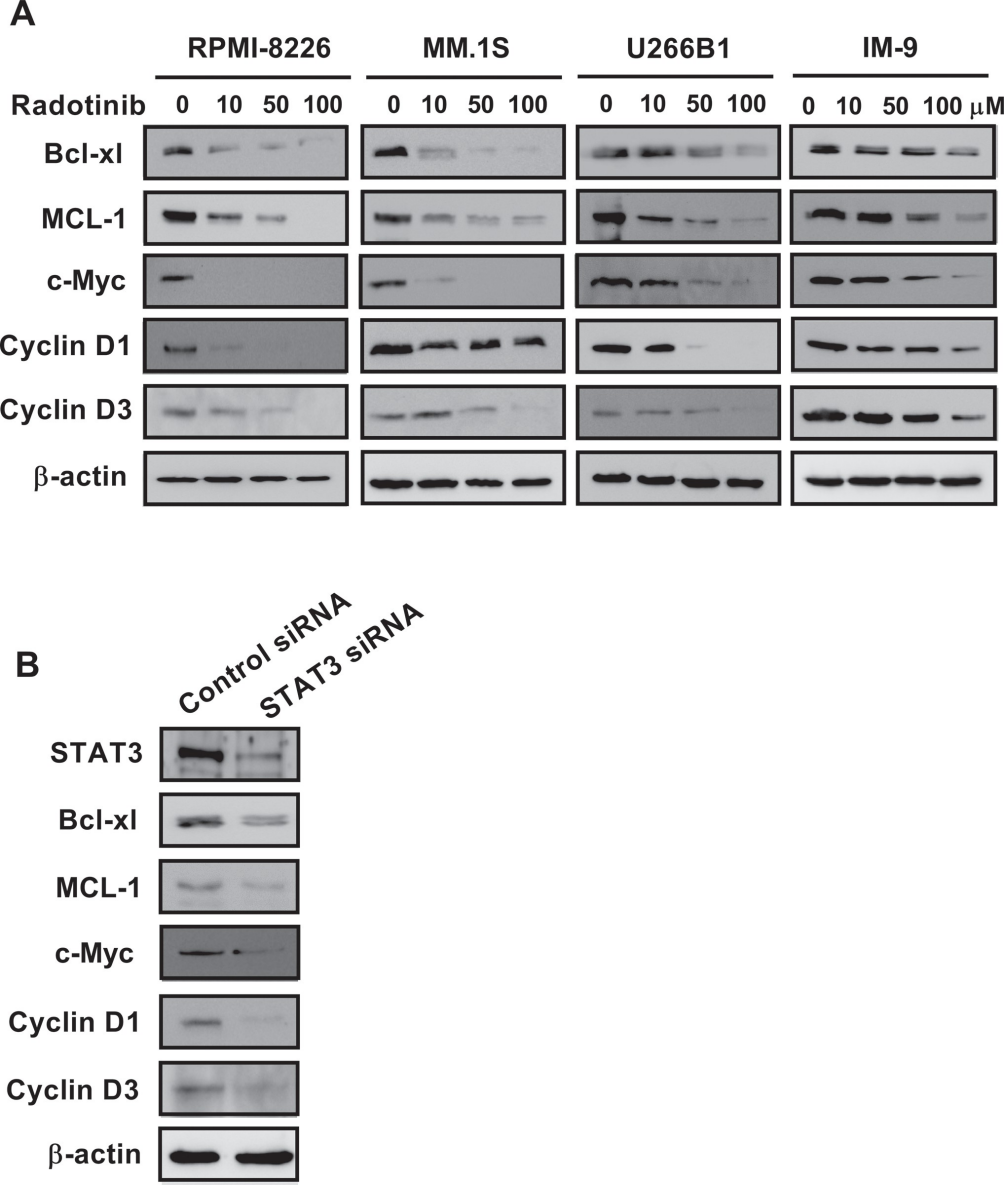
Radotinib strongly inhibits the activity and expression of STAT3 and JAK2 in various MM cells. (A) Effects of radotinib on the downstream proteins of STAT3 including expression of Bcl-xL, Mcl-1, c-Myc, cyclin D1, and cyclin D3. (B) Effects of STAT3 siRNA on MM cell proliferation. The membrane was stripped and reprobed with anti-β-actin mAb to confirm equal loading.

Human MM cells are well-known to express constitutively active STAT3 signaling [9,14,15]. The the survival factors, Bcl-xL, Mcl-1, and cell cycle regulators, c-Myc, cyclin D1, and cyclin D3 were identified as downstream targets of STAT3 [10–13]. Thus, we observed that the activity and expression of STAT3 and JAK2 was decreased by radotinib in a dose-dependent manner in various MM cells (Fig 3A and 3B). In addition, we observed that expression of Bcl-xL, Mcl-1, c-Myc, cyclin D1, and cyclin D3 levels was decreased by radotinib in a dose-dependent manner (Fig 4A). These results show that radotinib strongly inhibits the activity and expression of STAT3 and JAK2 in various MM cells (Figs 3 and 4A). To investigate the role of STAT3 in the inhibition of MM cell proliferation by radotinib, U266B1 cells were transfected with control siRNA and STAT3 siRNA using the Accell system. As shown in Fig 4B, the results demonstrated that MM cell proliferation was significantly reduced by STAT3 siRNA, suggesting that STAT3 activity and expression also play an important role in MM cell survival and proliferation. Therefore, based on Figs 1 and 2, radotinib inhibits multiple myeloma cell survival and proliferation via suppression of STAT3 and JAK2 signaling.

### Radotinib suppresses MM cell growth in a xenograft animal model using IM-9 cells

**Fig 5:**
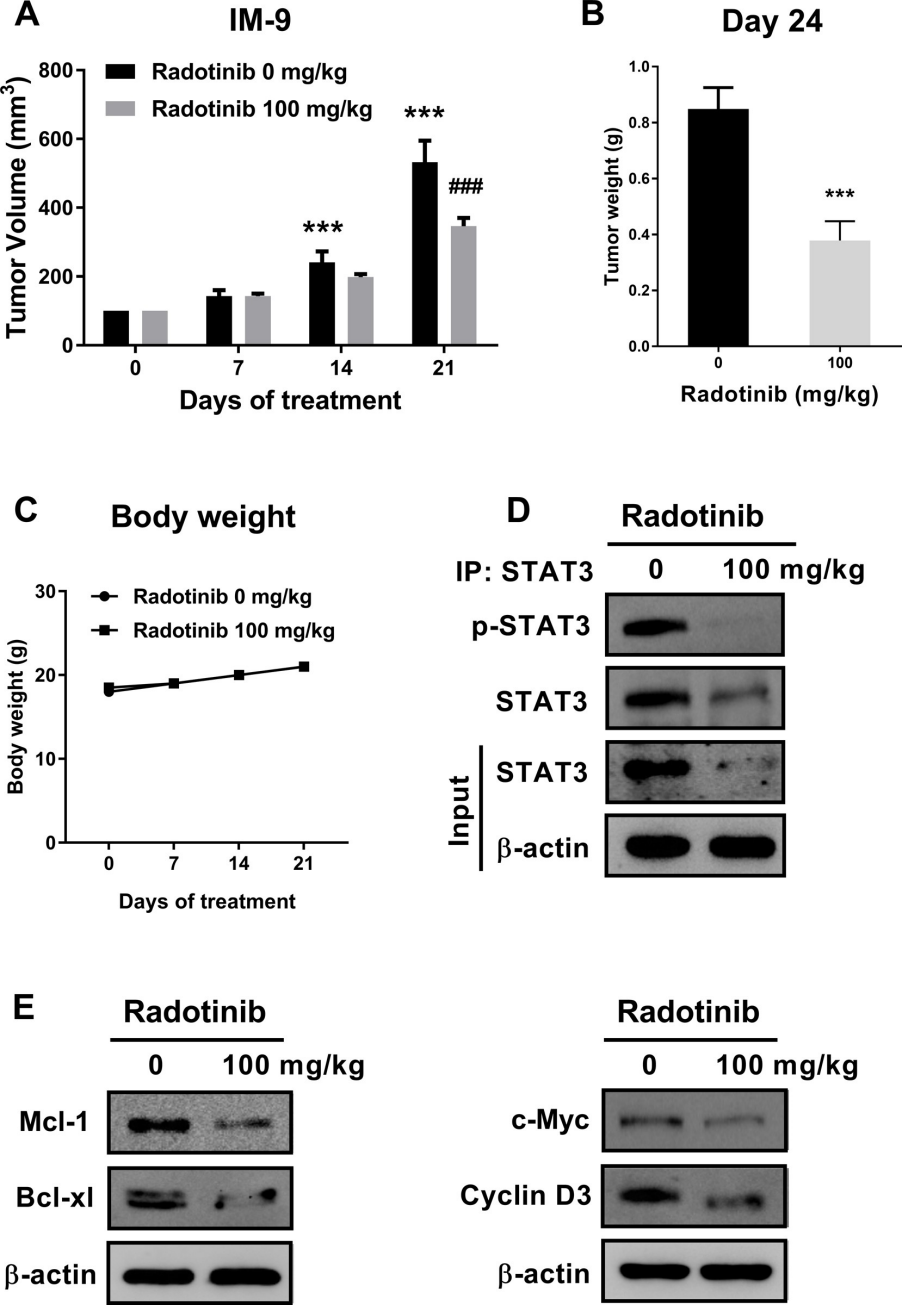
Radotinib significantly suppressed MM cell growth in a xenograft animal model (*n* = 5 for each group). (A) Tumor volume (mm^3^). (B) Tumor weight. (C) Body weight of mouse. (D) Effect of radotinib on the activity and expression of STAT3 in IM-9 cells isolated from the tumor tissue. (E) Effects of radotinib on the downstream proteins of STAT3 including expression of Mcl-1, Bcl-xL c-Myc, and cyclin D3 in IM-9 cells isolated from the tumor tissue. The membrane was stripped and reprobed with anti-β-actin mAb to confirm equal loading. Results are representative of three independent experiments. Data are presented as mean ± SEM. Significantly different from the day 0 control (*) or each day control (#); (*); ***, ###, *P* < 0.001.

**Fig 6:**
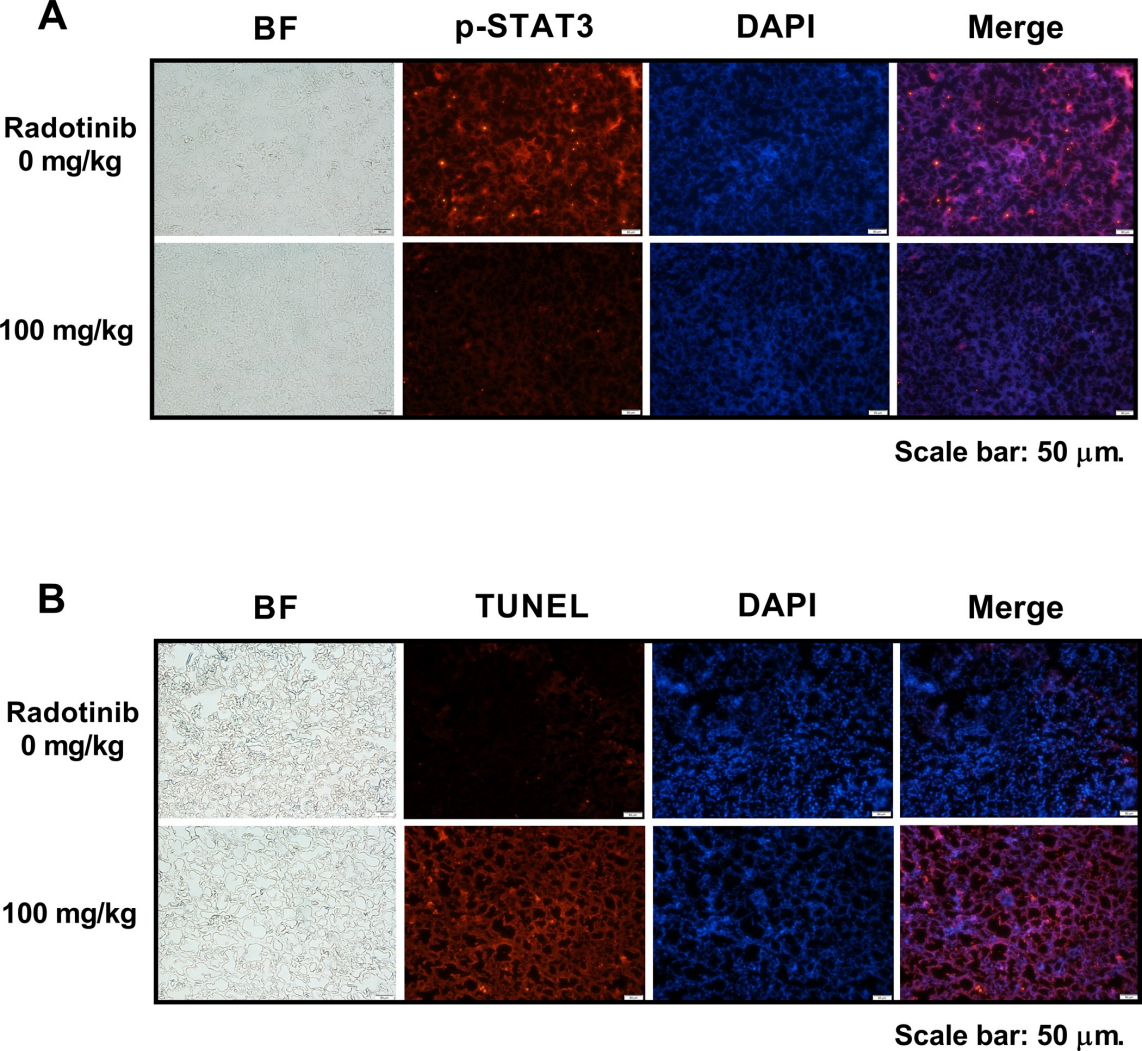
The effects of radotinib on the p-STAT3 and TUNEL positive cells expression in tumor tissue. (A) Measurement of p-STAT3 expression in tumor tissue. Radotinib significantly decreased the p-STAT3 expression in tumor tissue. Upper panel, radotinib 0 mg/kg; lower panel, radotinib 100 mg/kg. (B) Measurement of DNA double-strand breaks in tumor tissue. Radotinib significantly increased the DNA double-strand breaks in tumor tissue. TUNEL assay was used for measurement of DNA double-strand breaks in tumor tissue as described in the Methods. Upper panel, radotinib 0 mg/kg; lower panel, radotinib 100 mg/kg.

To further confirm the molecular mechanism of radotinib *in vivo*, we established murine models of multiple myeloma. As shown in Fig 5A and 5B, radotinib inhibited the growth of xenografted IM-9 cells in nude mice. The body weight of the tumor-bearing mice did not change significantly during the duration of this study, as shown in Fig 5C. In addition, radotinib repressed expression of the activity and expression of STAT3, and its downstream proteins including Bcl-xL, Mcl-1, c-Myc, cyclin D1, and cyclin D3 in IM-9 cells isolated from the tumor tissue (Fig 5D and 5E). In addition, the expression of phspho-STAT3-positive cells was dramatically reduced in the tumor tissue by100 mg/kg radotinib (Fig 6A). These results are similar to the results obtained using the western blotting assay of MM cells as shown in Figs 3 and 4. Furthermore, radotinib significantly increased the number of TUNEL-positive cells in the tumor tissue (Fig 6B). These results are similar pattern to the results obtained using the Annexin V staining of MM cells (Fig 1). Once again, these results suggest that radotinib significantly inhibits MM cell growth *in vivo* and may potentially be used for the treatment of MM in the future. Collectively, the data highlight the therapeutic possibility of radotinib in MM cells (Figs 5 and 6).

## Discussion

MM is a malignant disease characterized by accumulation of terminally differentiated plasma cells in the bone marrow [27,28]. According to classical protocols, treatment of MM is dependent on few drugs, such as lenalidomide, bortezomib, and dexamethasone. In recent years, new and more effective drugs have become available for the treatment of MM [3]. However, MM still recurs easily and is difficult to be cured. Therefore, there is a crucial need for identifying novel therapeutic options for MM.

Members of the STAT family have been connected in human cancer development, progression, metastasis, survival and resistance to treatment [29]. STAT3 and STAT5 are very important in cancer biology. STAT3 constitutively active in many human cancer cells including MM, leukemia, lymphoma, and solid tumors [30]. Activation of STAT3 has been found to be associated with shorter survival of the patients with MM [9]. Moreover, aberrant activation of STAT3 is frequently observed in MM cancer and can upregulate the expression of several genes involved in proliferation, survival, and metastasis. In addition, STAT3 overexpression in MM appears to be mediated by a variety of factors and may be associated with an adverse prognosis and may play a role in microenvironment-dependent treatment resistance.

In MM STAT3 overexpression may be associated with an unfavorable prognosis appears to be mediated by a variety of factors may play a role in the microenvironment-dependent treatment-resistant.

STAT3 overexpression in MM appears to be mediated by a variety of factors and may be associated with an adverse prognosis and may play a role in microenvironment-dependent treatment resistance [15]. Therefore, we thought that it could be one of the effective treatment protocols if the activated STAT3 and JAK2 signals in multiple myeloma can be controlled with radotinib. We were also able to confirm once again that STAT3 could be a new molecular target for the treatment of multiple myeloma [14,15,30]. Also, we are interested in how radotinib regulates the expression and activity of STAT3, and we think that further study of potential mechanisms is needed in future studies. Also, we are paying attention to the co-culture of multiple myeloma cells and bone marrow-derived stromal cells, and we believe that further studies are needed in the surrounding environment.

Radotinib is approved in South Korea for its use as a second-line treatment of CML. Its mechanism of action involves inhibition of the tyrosine kinase Bcr-Abl and of platelet-derived growth factor receptor. Little is known about the effects of radotinib on multiple myeloma cells. However, little is known about the effects of radotinib on multiple myeloma cells.

But even tinip almost not known about the impact of multiple myeloma cells.

Moreover, nothing is known about how it affects STAT3 and JAK2. The purpose of this study was to evaluate whether the potential anticancer effects of radotinib, as known a tyrosine kinase inhibitor, in MM was mediated primarily through blockade of the STAT3 and JAK2 signaling pathway *in vitro* and *in vivo*. Herein, Moreover, nothing is known about how it affects STAT3 and JAK2.

Moreover, not all is known about how the affects STAT3 and JAK2.

We examined the effect of radotinib on the STAT3 signaling pathway in MM cells, Interestingly, radotinib caused death of MM cells (Fig 1A). Radotinib increased the number of Annexin V positive cells, indicating that cells undergo death via apoptosis (Fig 1B). Moreover, radotinib suppressed MM cell proliferation in diverse MM cell lines including RPMI-8226, MM.1S, U266B1, and IM-9 cells (Fig 2A). In addition, it induced G0/G1-phase cell cycle arrest, and finally reduced the proliferation rate of MM cells (Fig 2B). Furthermore, radotinib treatment remarkably decreased the activity and expression of STAT3 and JAK2 and resulted in a dose-depended reduction of STAT3-targeted proteins in RPMI-8226, MM.1S, U266B1, and IM-9 cells (Figs 3 and 4). Therefore, these results indicate that radotinib inhibits multiple myeloma cell proliferation via suppression of STAT3 and JAK2 signaling. To further validate the molecular mechanism of action of radotinib *in vivo*, we established xenograft murine models using the human multiple myeloma cell line IM-9. We confirmed that radotinib had anti-tumor activities in mice bearing IM-9 cells Furthermore, radotinib significantly inhibited MM cell growth *in vivo* in a xenograft model (Fig 5). More interestingly, radotinib inhibited the STAT3-targeted proteins such as Bcl-xl, Mcl-1, c-Myc, and cyclin D3 expression in IM-9 cells isolated from the tumor tissue (Fig 5). In addition, STAT3 activity (phospho-STAT3 Tyr705) was dramatically reduced, while the expression of TUNEL-positive cells was significantly amplified by radotinib in the tumor tissue (Fig 6). Thus, radotinib inhibits MM cell proliferation via suppression of the STAT3 and JAK2 activity and expression. Consequently, these results suggest that radotinib functions as an STAT3 or JAK2 inhibitor in MM cells.

We considered that it was important to determine the expression level of the target molecule for radotinib in the multiple myeloma cell lines RPMI-8226, MM.1S, U266B1 and IM-9 cells. The relative expression levels of STAT3 in RPMI-8226, MM.1S, U266B1 and IM-9 cells were determined by Western blotting. As shown in S1 Fig, STAT3, the target molecule of radotinib, was expressed in all RPMI-8226, MM.1S, U266B1 and IM-9 cells. In particular, the relative expression of STAT3 was high in RPMI-8226 cells. Interestingly, the expression of STAT3 was very high in RPMI-8226 cells with high levels of apoptosis. And apoptosis was lowest in IM-9 cells with the lowest STAT3 expression. Therefore, these results mean that the higher the STAT3 expression, the higher the apoptosis caused by radotinib.

It has been well known that STAT3 is involved in variety of biological process including cell proliferation, differentiation, inflammatory responses, and angiogenesis [31]. Furthermore, we quantitatively evaluated the mRNA expression of VEGF and MMP-9, which affects angiogenesis, using tumor tissues obtained from in vivo experiments in xenograft models using the multiple myeloma cell lines RPMI-8226 and IM-9. It was detected by an experimental method using real-time reverse transcription polymerase chain reaction. The primer sequences are shown in S1 Table. As a result, the mRNA expression of VEGF and MMP-9 on day 24 was significantly lower in the tumor tissues of the radotinib 100 mg/kg group compared to the control group, radotinib 0 mg/kg group. These results were shown in S2 Fig. These results are thought to be naturally expected results as VEGF and MMP-9 are downstream proteins affected by STAT3.

According to a recent preclinical study, INCB052793 (a selective JAK1 inhibitor) exhibited anti-MM activity in both *in vivo* and *in vitro* experiments [32]. Momelotinib (CYT387), a JAK1 and JAK2 inhibitor, also showed strong anti-cancer activity by significantly reducing MM proliferation in preclinical results. [33]. In addition, when the above two JAK1 inhibitors were used together with the conventional MM therapy, they succeeded in killing MM cells [32,33]. Analyzing our results based on the results of other research teams that the JAK inhibition has the therapeutic effect of multiple myeloma, we are expected that radotinib will play an important role in the treatment of multiple myeloma as the radotinib has an excellent effect on STAT3 and JAK2 inhibition. In our previous MM study, we found that there was a strong synergism between radotinib, bortezomib, and dexamethasone in combination [18]. In addition, we think that more research is needed on the combination effect with melphalan or lenalidomide.

It has been well known that MM cells are dependent on Myc for survival [34–36]. Specifically, c-Myc is activated and contributes to the malignant phenotype in multiple myeloma [35]. Holien, et al. showed the evidence that myeloma cells are addicted to c-Myc activity, and c-Myc is a promising therapeutic target in multiple myeloma [35]. In this context, we think medicines that can actively lower the expression of c-Myc can be a very good candidate for treatment of multiple myeloma. As shown in Fig 3C, radotinib strongly inhibits the expression of c-Myc in various MM cells including RPMI-8226, MM.1S, U266B1, and IM-9 cells. These results are important results showing that radotinib has strong potential as a treatment for multiple myeloma.

In conclusion, radotinib inhibits multiple myeloma cell proliferation via suppression of STAT3, JAK2 and c-Myc signaling. Therefore, our results indicate that radotinib can abrogate MM cell growth both *in vitro* and *in vivo* and may serve as a candidate agent for the treatment of MM. This study provides the first evidence that radotinib could be used as an effective therapeutic agent to treat MM patients.

## Supporting information

S1 Fig The relative expression levels of STAT3 in RPMI-8226, MM.1S, U266B1, and IM-9 cells were analyzed by Western blotting. The membrane was stripped and reprobed with anti-β-actin mAb to confirm equal loading.
(TIF)

S2 Fig Radotinib significantly suppressed mRNA expression of VEGF and MMP9 in tumor tissue from a xenograft animal model. The mRNA expression of VEGF (A), and MMP9 (B) was determined by quantitative real-time reverse transcription-polymerase chain reaction (QRT-PCR). Data were normalized to GAPDH mRNA and represent the fold change relative to the control. Data are presented mean ± SEM.; ***, *P* < 0.001. *Significantly different from control cells. VEGF, Vascular endothelial growth factor; MMP9, Matrix metallopeptidase 9; GAPDH, Glyceraldehyde 3-phosphate dehydrogenase.
(TIF)

S1 Table Sequences of primers used for quantitative RT-PCR. (DOCX)

S1 Raw images (PDF)

S2 Raw images (PDF)

S3 Raw images (PDF)

## Abbreviations

MM: Multiple myeloma
STAT3: signal transducer and activator of transcription 3
JAK2: Janus kinase 2
TKI: Tyrosine kinase inhibitor
CML: chronic myeloid leukemia
AML: acute myeloid leukemia
