# Supplementary figures and images for: Radotinib inhibits multiple myeloma cell proliferation via suppression of STAT3 signaling
Source: PLoS One. 2022 May 3;17(5):e0265958. doi: 10.1371/journal.pone.0265958 (PMC9064077; doi:10.1371/journal.pone.0265958)

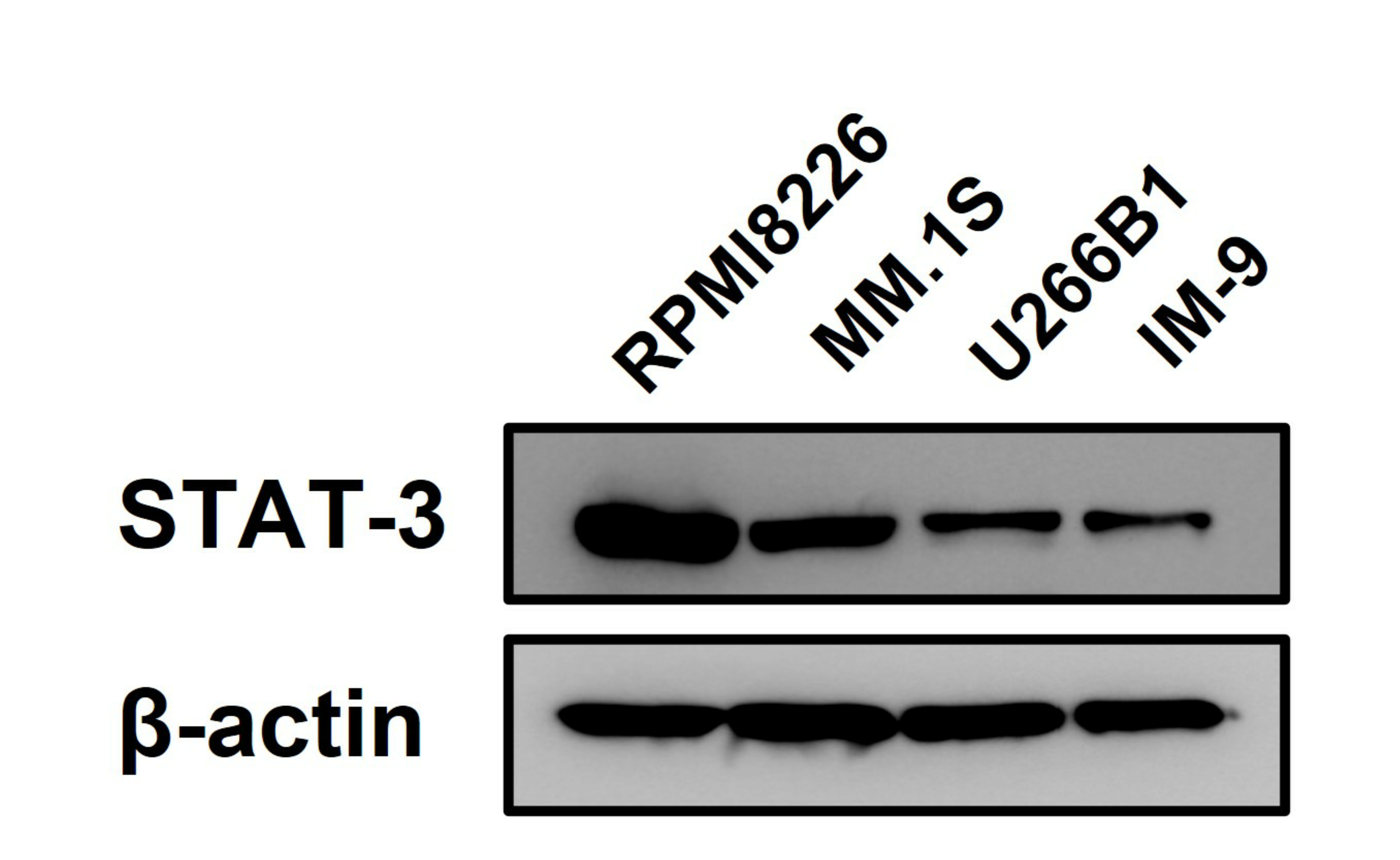

Supplement: S1 Fig — The membrane was stripped and reprobed with anti-β-actin mAb to confirm equal loading. (TIF) [file pone.0265958.s001.tif]

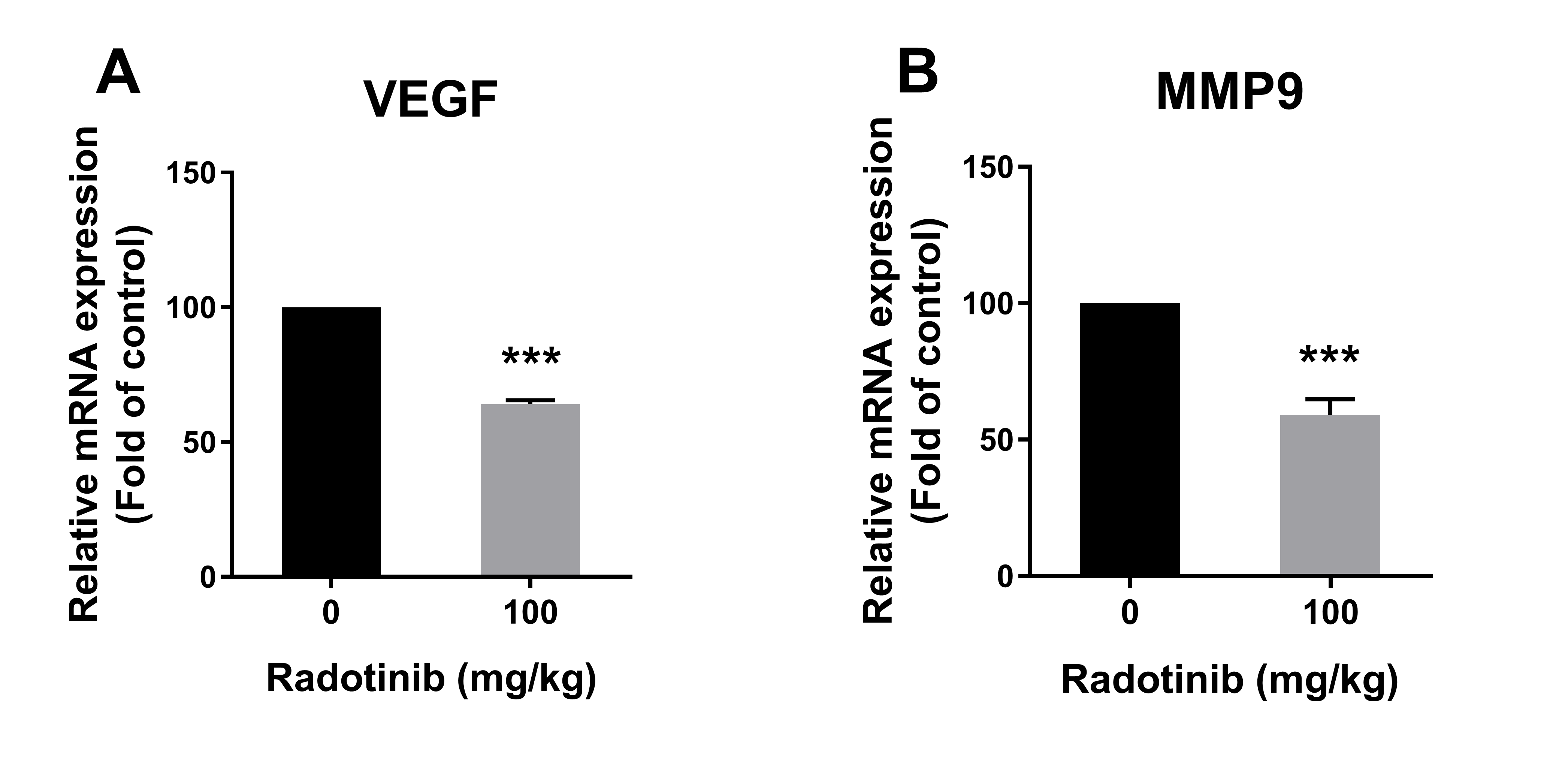

Supplement: S2 Fig — The mRNA expression of VEGF (A), and MMP9 (B) was determined by quantitative real-time reverse transcription-polymerase chain reaction (QRT-PCR). Data were normalized to GAPDH mRNA and represent the fold change relative to the control. Data are presented mean ± SEM.; ***, P < 0.001. *Significantly different from control cells. VEGF, Vascular endothelial growth factor; MMP9, Matrix metallopeptidase 9; GAPDH, Glyceraldehyde 3-phosphate dehydrogenase. (TIF) [file pone.0265958.s002.tif]
