# Supplementary material for: Radotinib inhibits multiple myeloma cell proliferation via suppression of STAT3 signaling
Source: PLoS One. 2022 May 3;17(5):e0265958. doi: 10.1371/journal.pone.0265958 (PMC9064077; doi:10.1371/journal.pone.0265958)
Supplement: S1 Table — (DOCX) [file pone.0265958.s003.docx]

**S1 Table. Sequences of primers used for quantitative RT-PCR.**

| Target gene | Primer sequences (5’ →3’) | |
| --- | --- | --- |
| VEGF | **Forward** | **ACACATTGTTGGAAGAAGCAGCCC** |
|  | **Reverse** | **AGGAAGGTCAACCACTCACACACA** |
| MMP9 | **Forward** | **ACGATGCCTGCAACGTGAACATCT** |
|  | **Reverse** | **TCGCCAGTACTTCCCATCCTTGAACA** |
| GAPDH | **Forward** | **GATCATCAGCAATGCCTCCT** |
|  | **Reverse** | **GTCATGAGTCCTTCCACGATAC** |

VEGF, Vascular endothelial growth factor; MMP9, Matrix metallopeptidase 9; GAPDH, Glyceraldehyde 3-phosphate dehydrogenase.
