# Supplementary material for: Radotinib inhibits multiple myeloma cell proliferation via suppression of STAT3 signaling
Source: PLoS One. 2022 May 3;17(5):e0265958. doi: 10.1371/journal.pone.0265958 (PMC9064077; doi:10.1371/journal.pone.0265958)

Fig. 3A. Heo et al

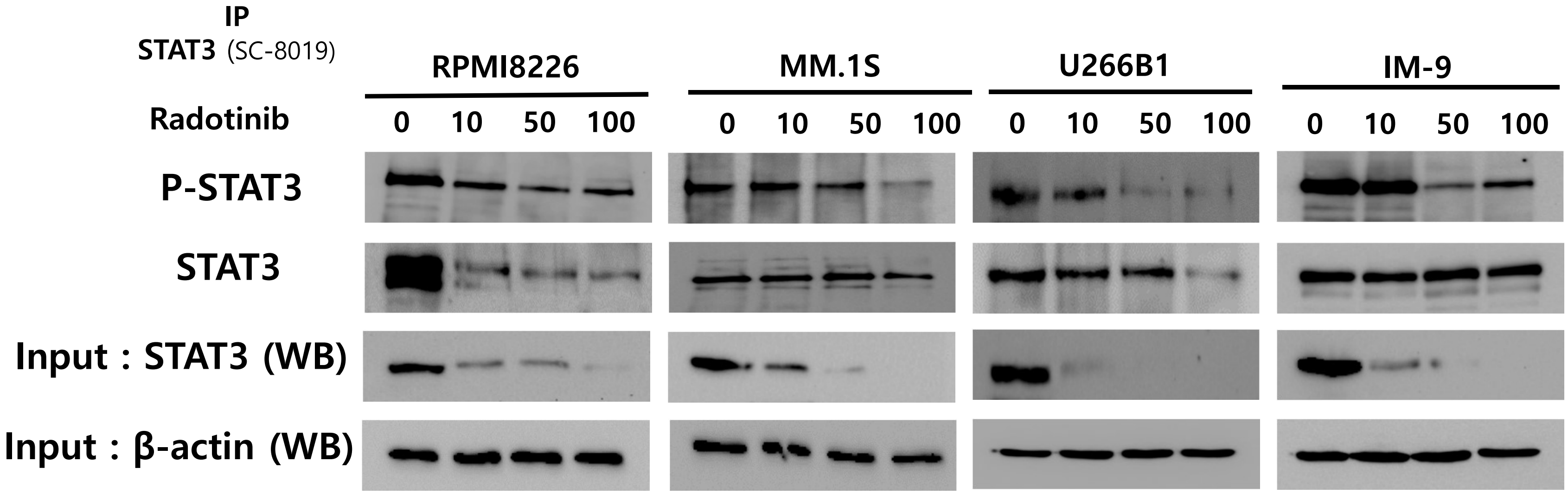

RPMI8226

RPMI8226 20180809 P-STAT-3(SC-8059) 1:200 dilution with 5% skim milk in PBST
2nd ab: Rabbit

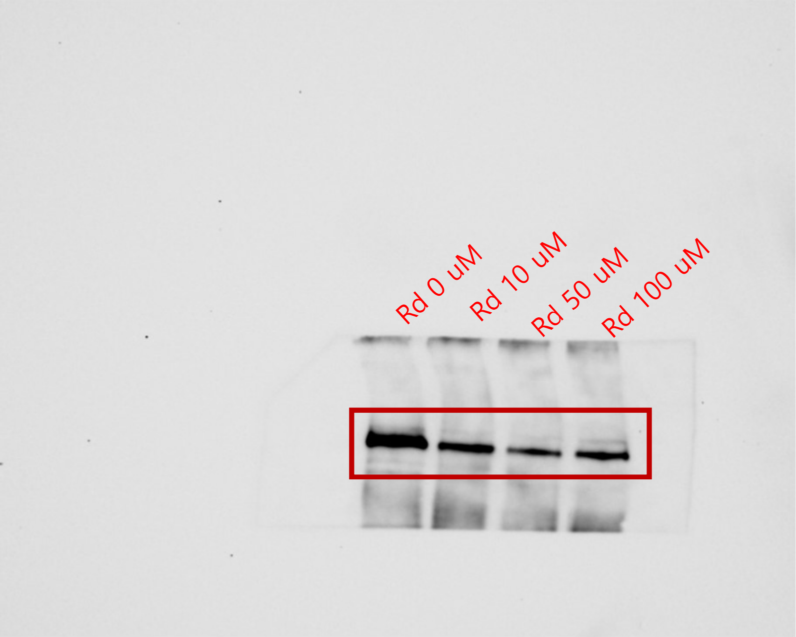

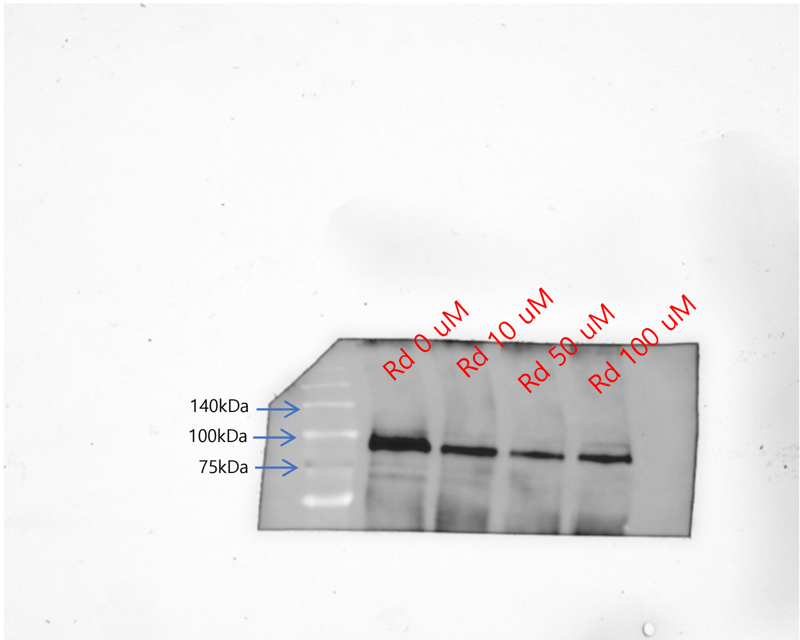

RPMI8226 20191127 STAT3 (ab-226942) 1:500 dilution with 5% skim milk in PBST
2nd ab: Rabbit

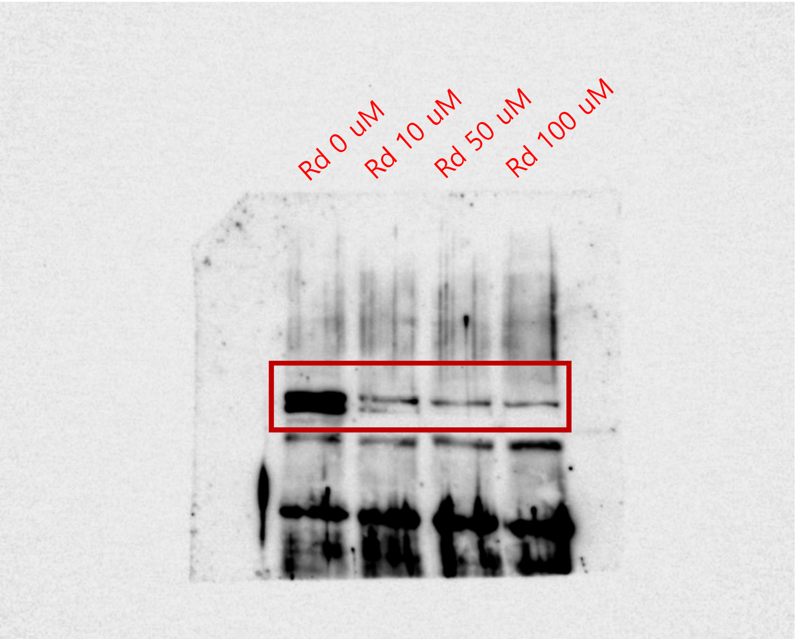

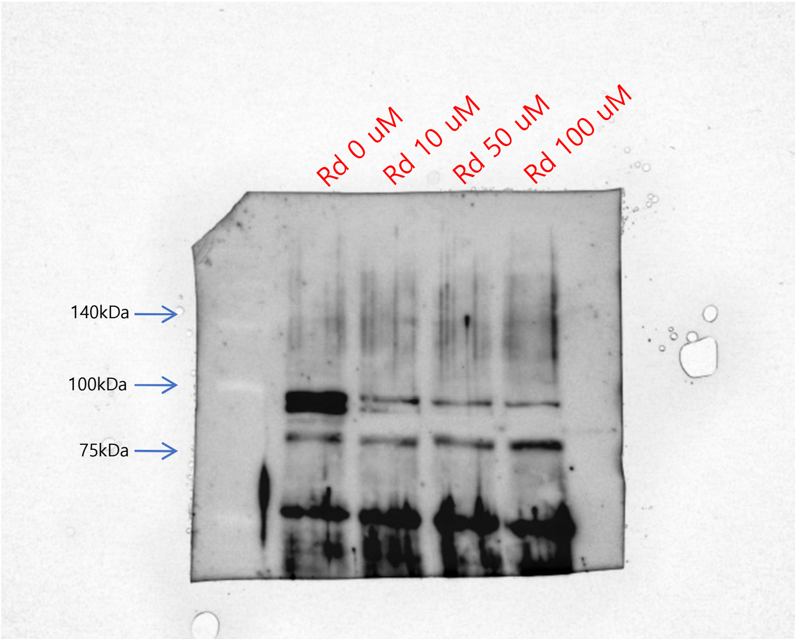

RPMI8226 20200826 STAT3 (CST-12640) 1:500 dilution with 5% skim milk in PBST
2nd ab: Rabbit

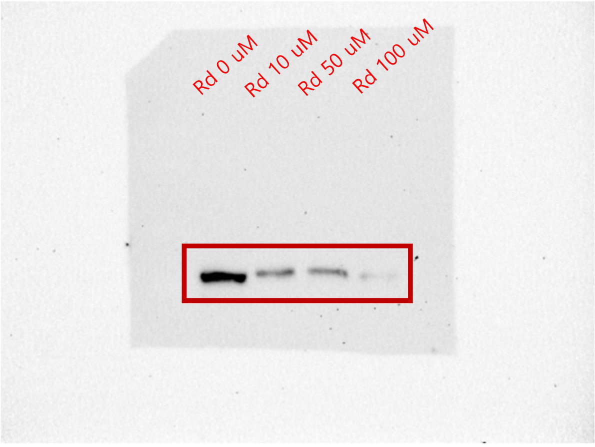

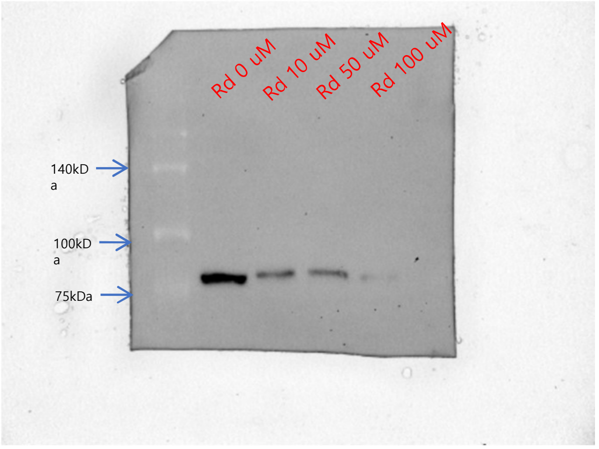

RPMI8226 20200915 β-actin(SC-47778) 1:200 dilution with 5% skim milk in PBST
2nd ab: Mouse

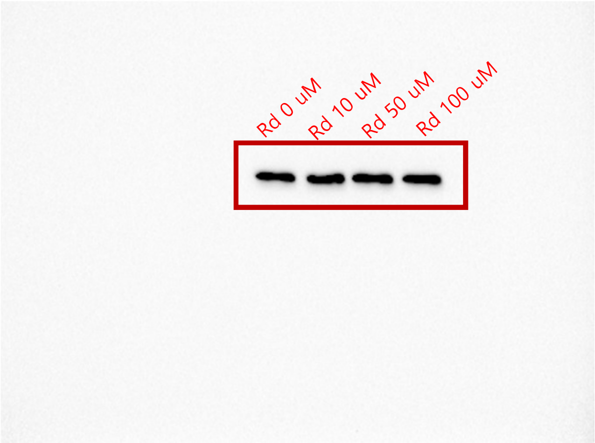

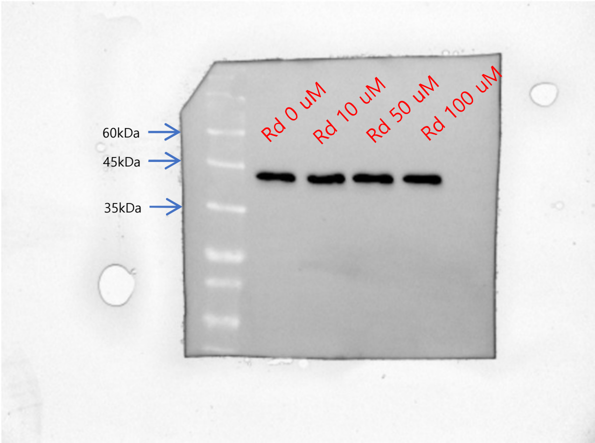

MM.1S

MM.1S_20191122 P-STAT3 (SC-8059) 1:200 dilution with 5% skim milk in PBST
2nd ab: Mouse

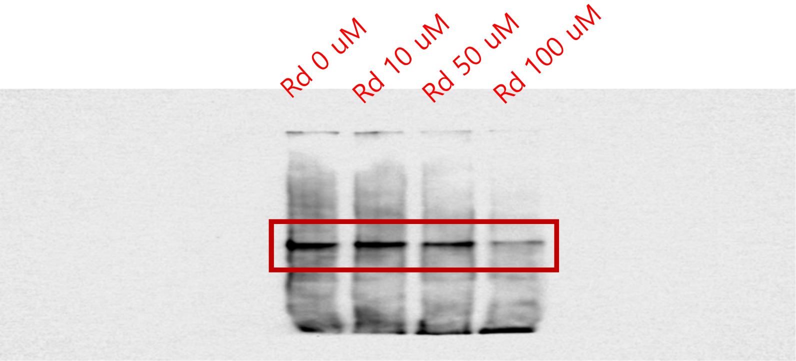

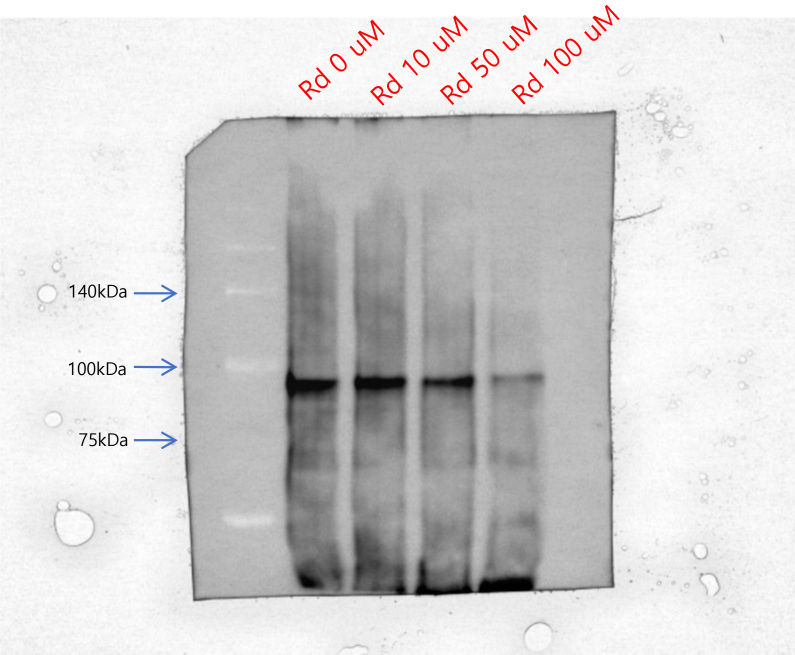

MM.1S_20191206 STAT3 (CST-12640) 1:500 dilution with 5% skim milk in PBST
2nd ab: Rabbit

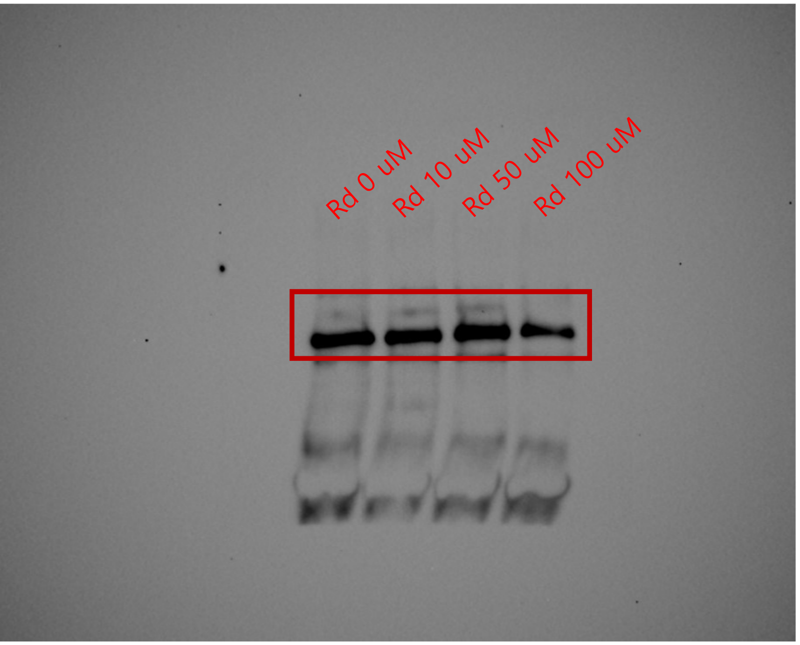

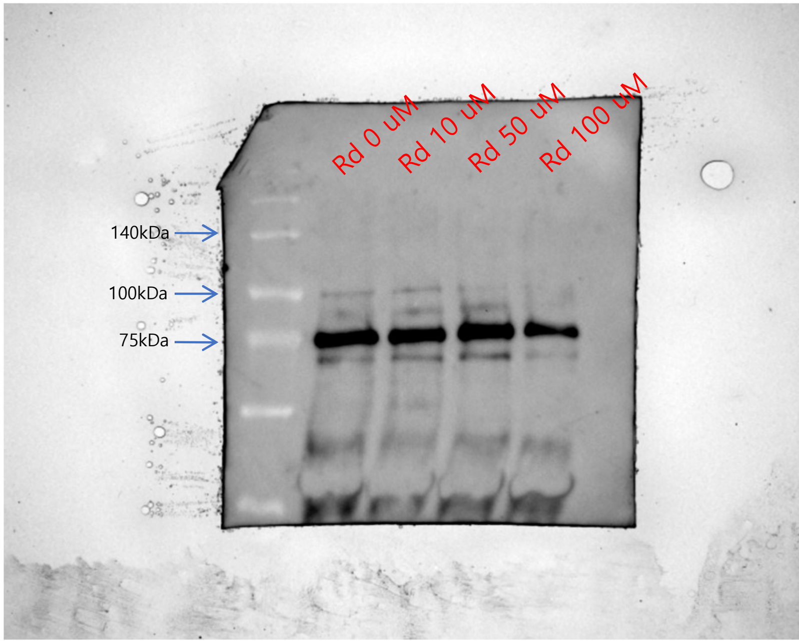

MM.1S 20200916 STAT3 (SC-8019) 1:500 dilution with 5% skim milk in PBST
2nd ab: Mouse

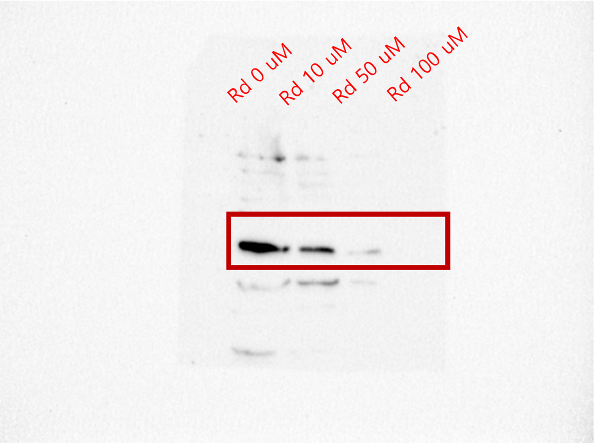

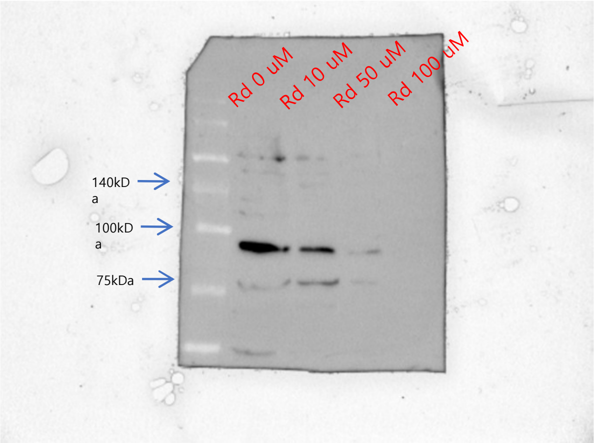

MM.1S 20200909 β-actin(SC-47778) 1:200 dilution with 5% skim milk in PBST
2nd ab: Mouse

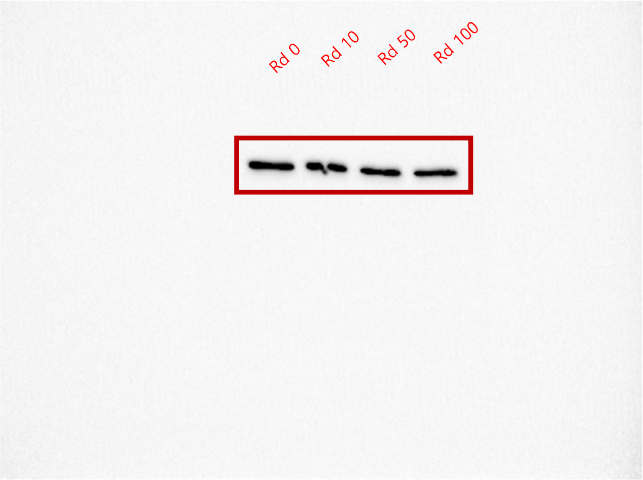

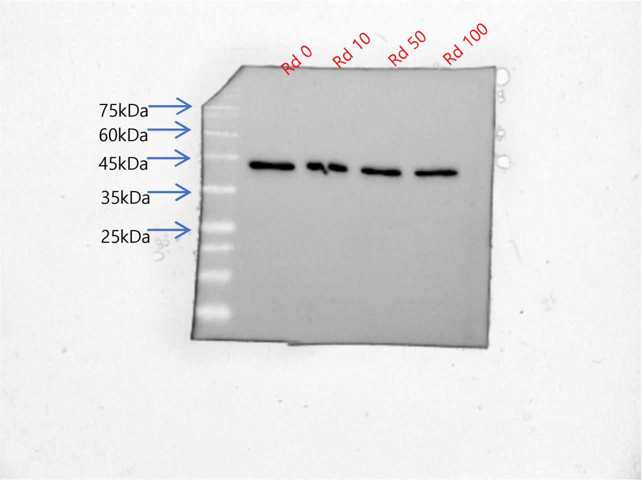

U266B1

U266B1 20210202 p-STAT3(CST-9145) 1:500 dilution with 5% skim milk in PBST
2nd ab: Rabbit

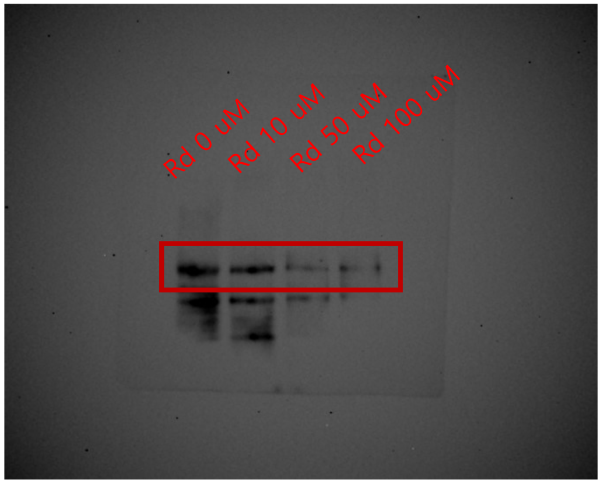

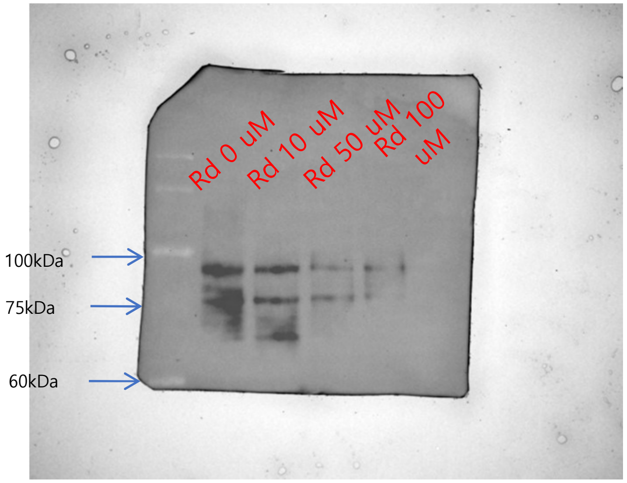

U266B1 20200521 STAT3(ab-226942) 1:500 dilution with 5% skim milk in PBST
2nd ab: goat

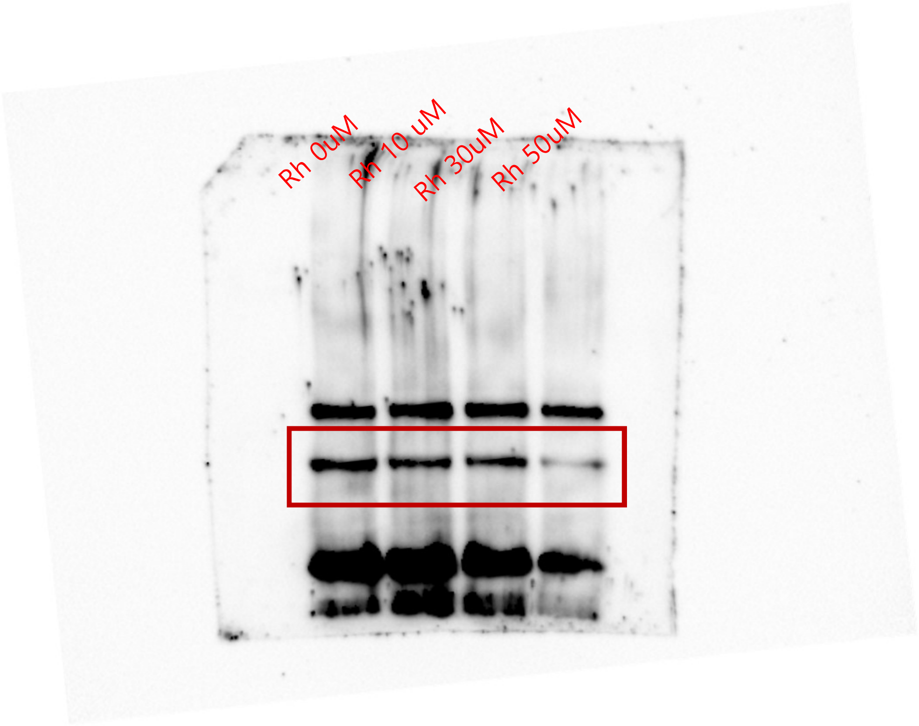

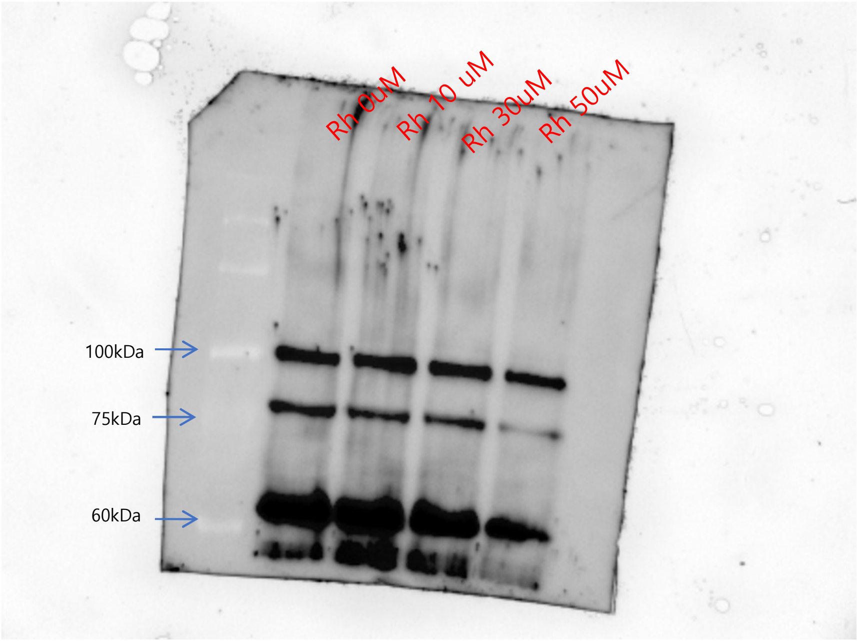

U266B1 20201216 STAT3 (SC-8019) 1:200 dilution with 5% skim milk in PBST
2nd ab: Mouse

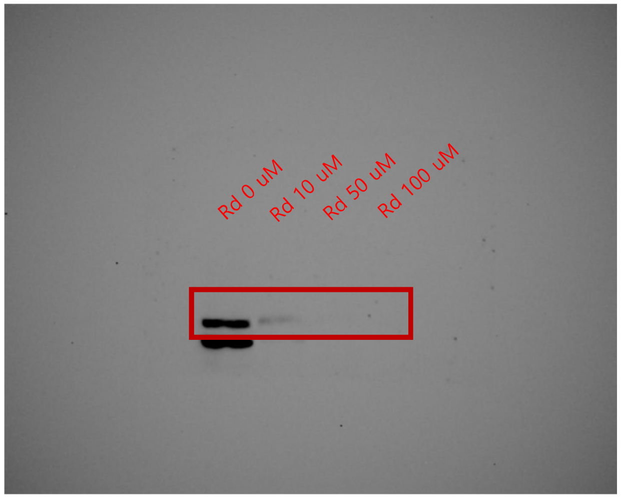

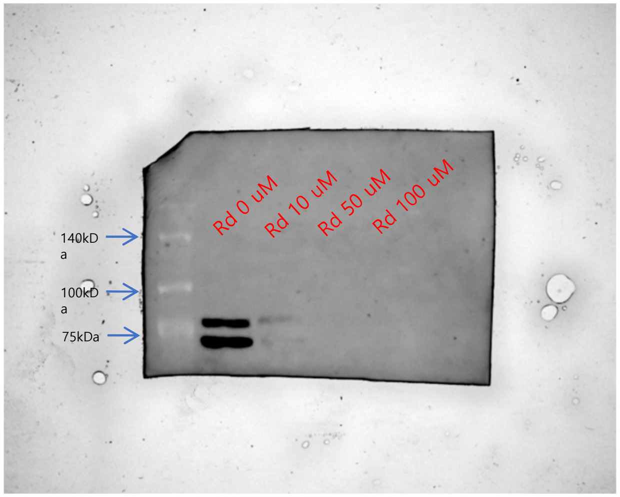

U266B1 20210106 β-actin(SC-47778) 1:200 dilution with 5% skim milk in PBST
2nd ab: Mouse

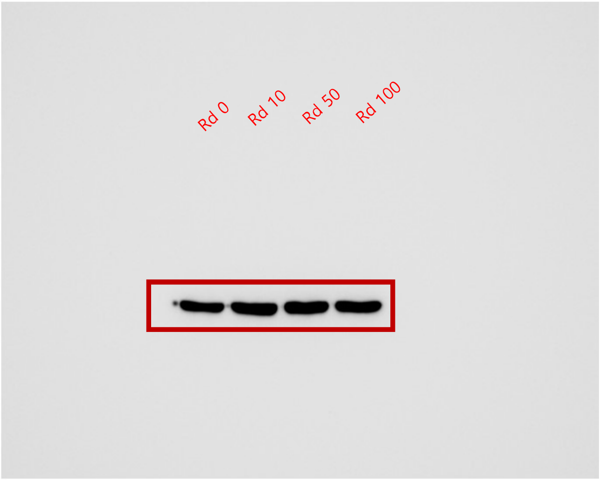

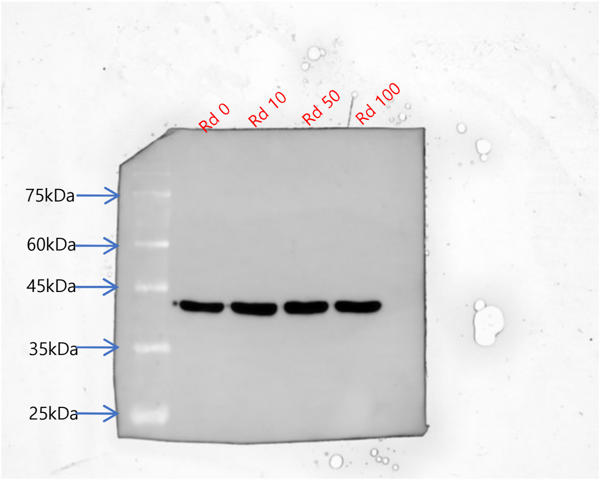

IM-9

IM-9 20191203 p-STAT3(SC-8059) 1:200 dilution with 5% skim milk in PBST
2nd ab: Mouse

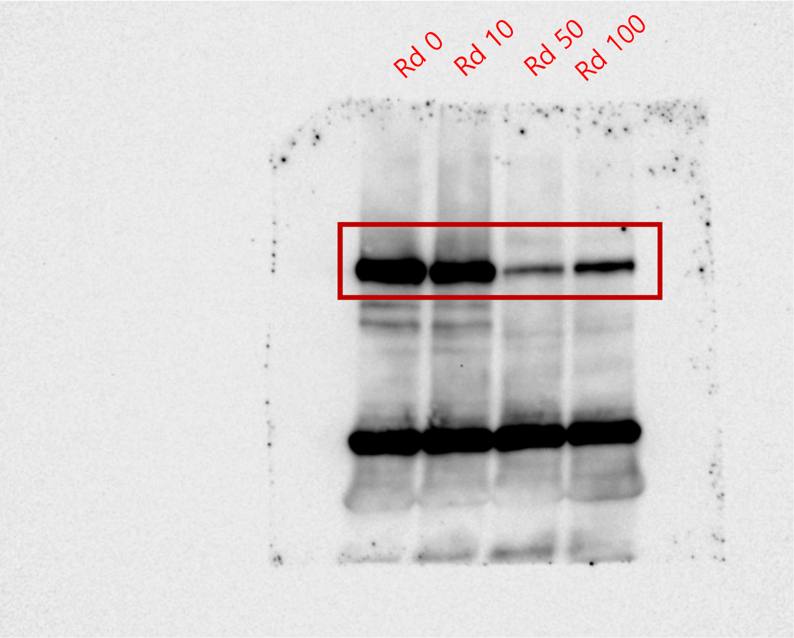

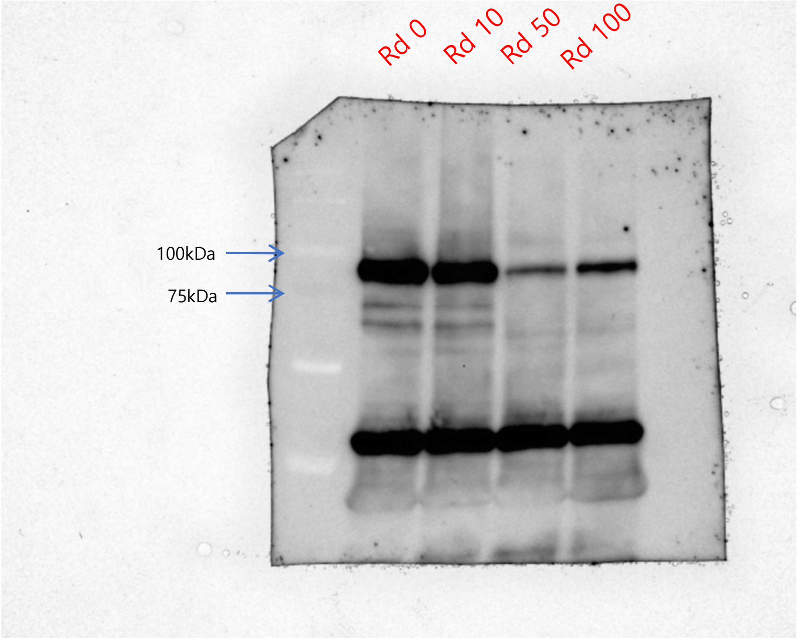

IM-9 20191204 STAT3(CST-12640) 1:500 dilution with 5% skim milk in PBST
2nd ab: Mouse

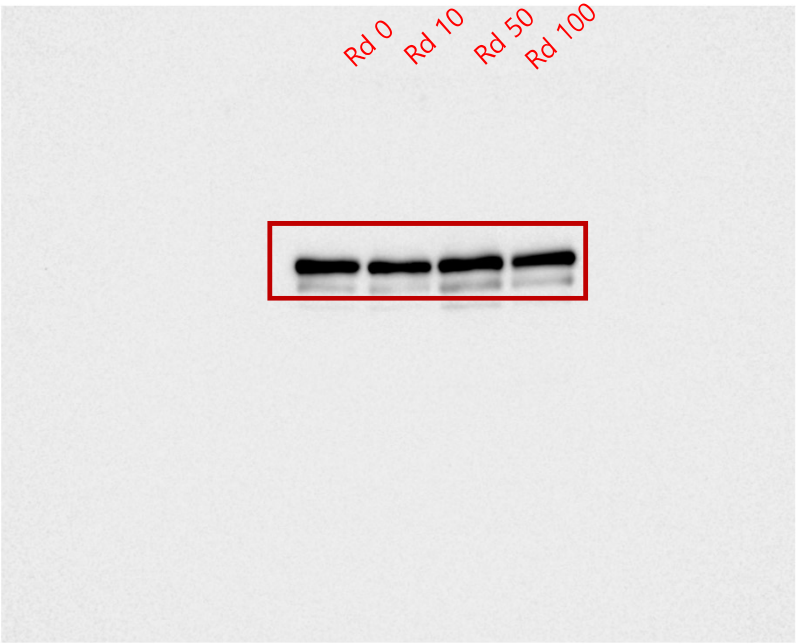

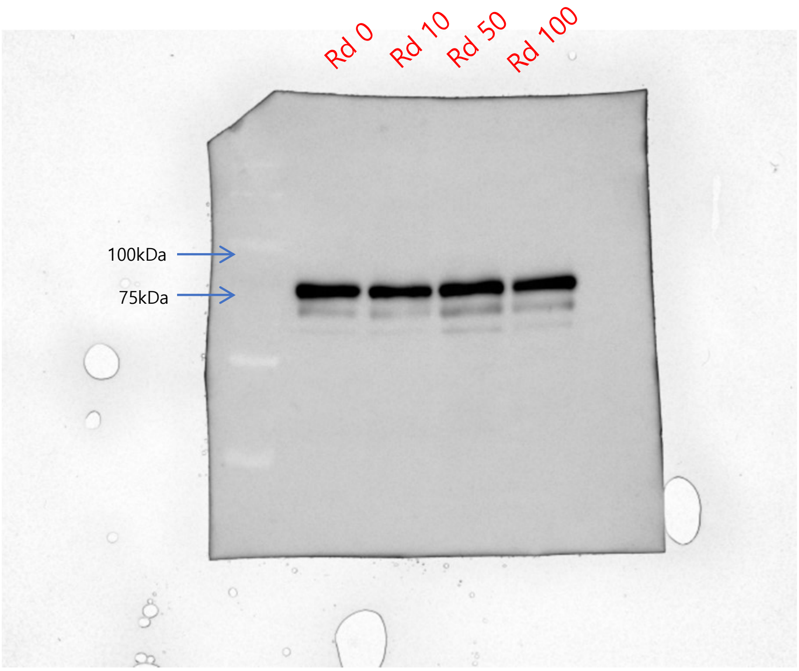

IM-9 20200922 STAT-3(CST-12640) 1:500 dilution with 5% skim milk in PBST
2nd ab: Rabbit 79, 86kda 8%

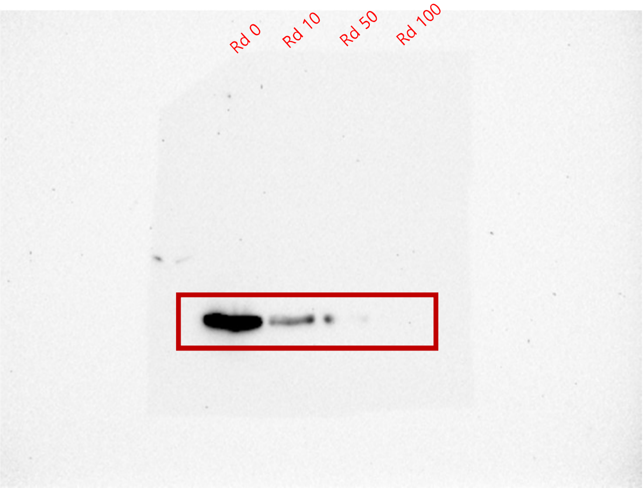

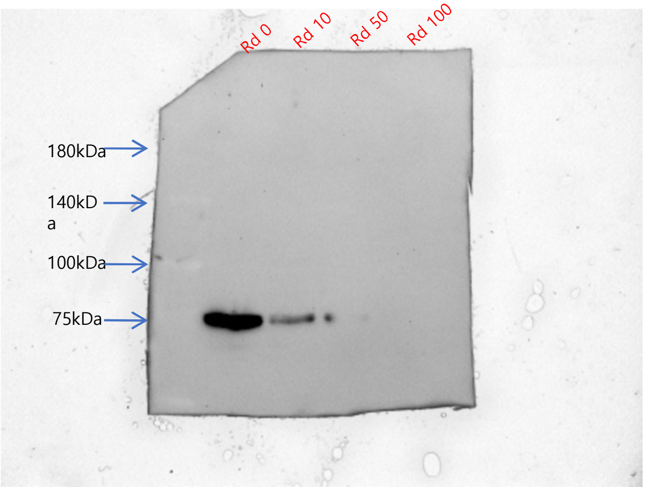

IM-9 20180608 β-actin(SC-47778) 1:200 dilution with 5% skim milk in PBST
2nd ab: Mouse

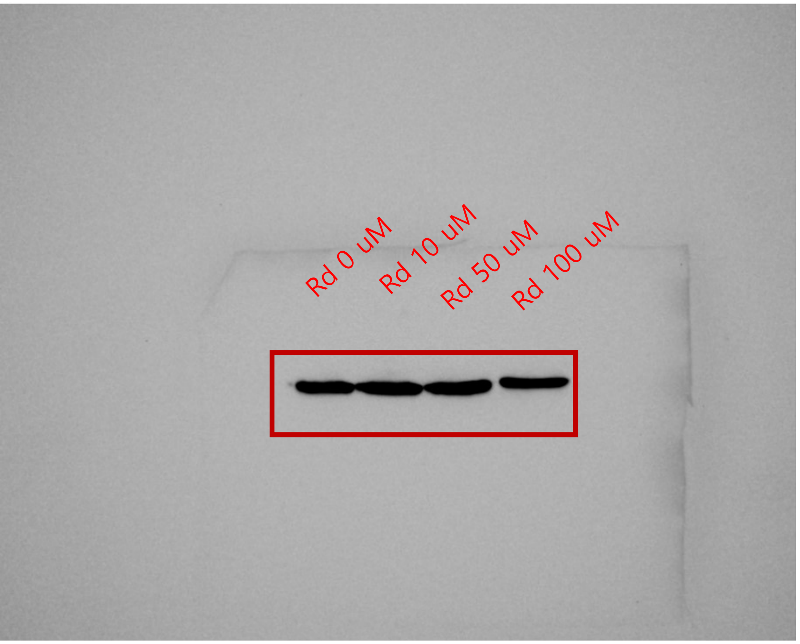

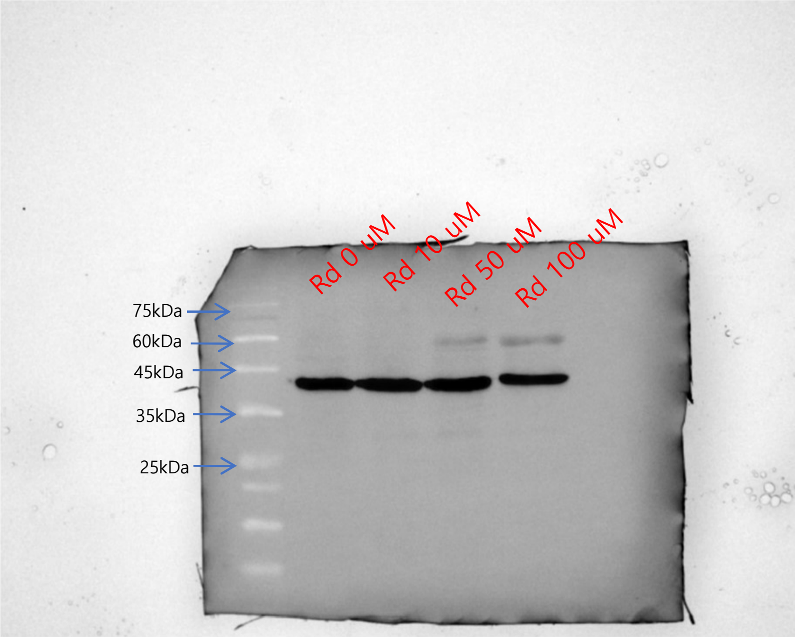

Fig. 3B. Heo et al

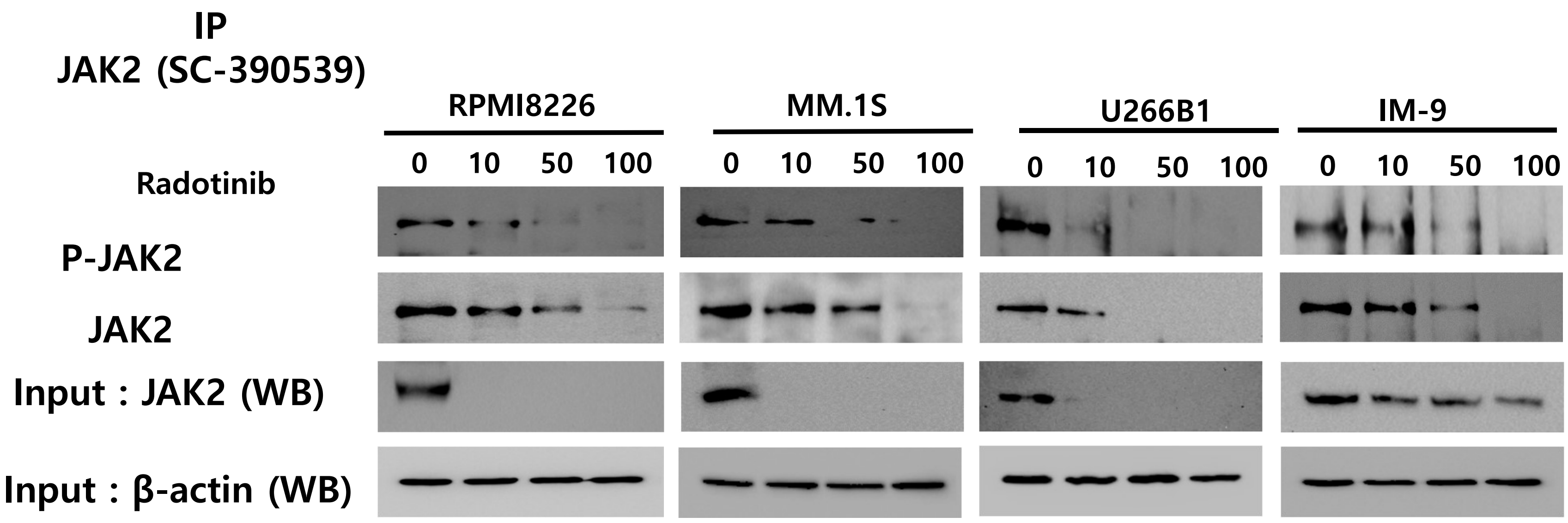

RPMI8226

RPMI8226_20200211 P-JAK2 (CST-8082) 1:500 dilution with 5% skim milk in PBST
2nd ab: Rabbit

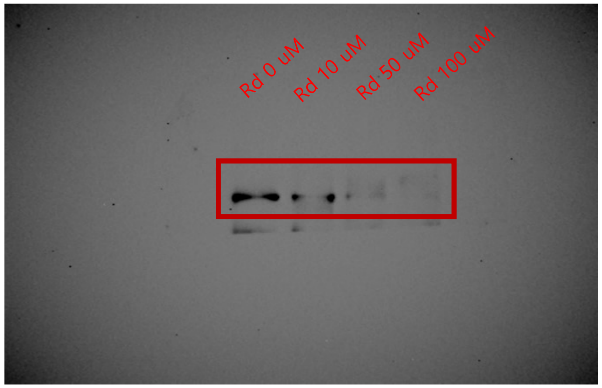

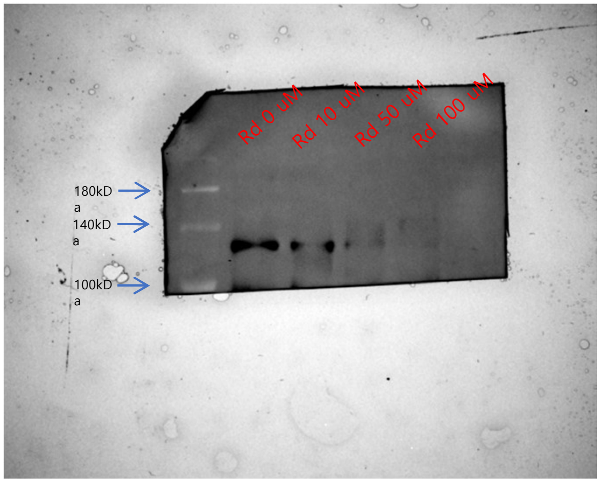

RPMI8226_20200207 JAK2 (CST-3230) 1:500 dilution with 5% skim milk in PBST
2nd ab: Rabbit

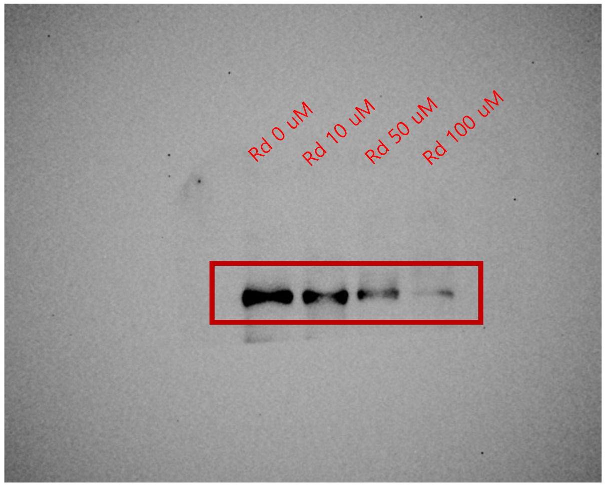

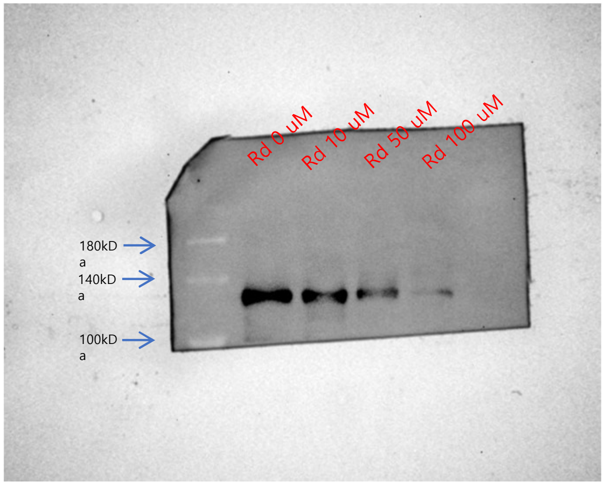

RPMI8226 20201104 β-actin(SC-47778) 1:200 dilution with 5% skim milk in PBST

2nd ab: Mouse

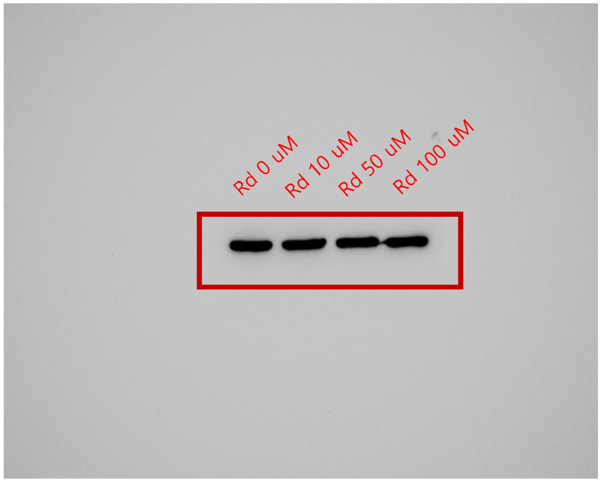

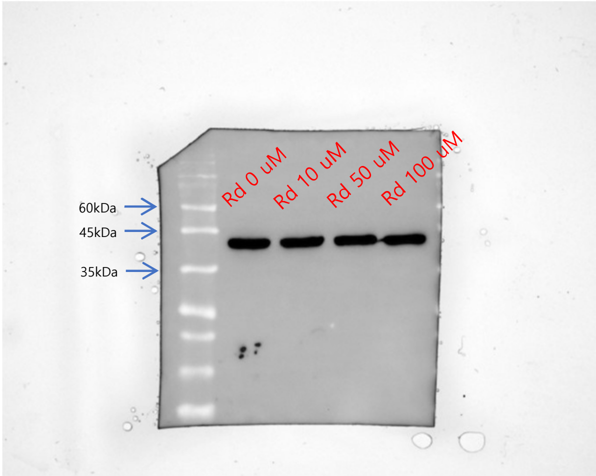

RPMI8226 20201119 JAK2 (CST-3230) 1:500 dilution with 5% skim milk in PBST

2nd ab: Rabbit

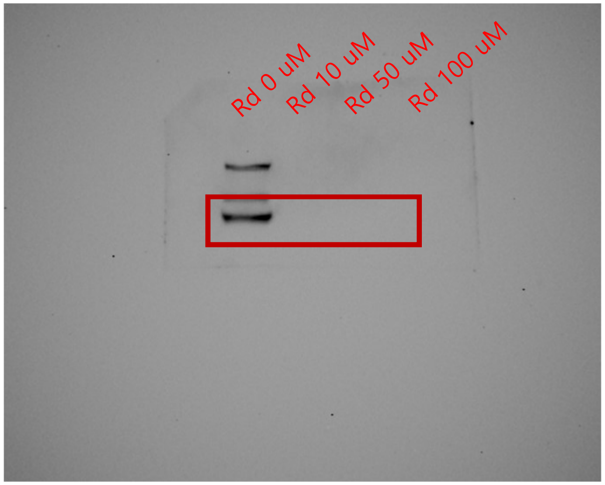

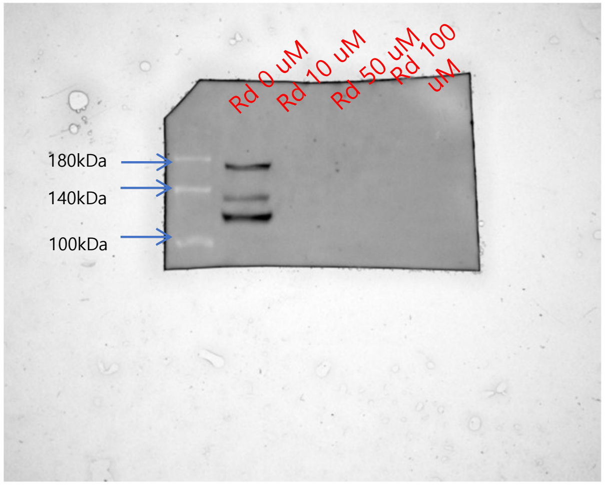

MM.1S

MM.1S_20200212 P-JAK2(CST-8082) 1:500 dilution with 5% skim milk in PBST 2nd ab: Rabbit

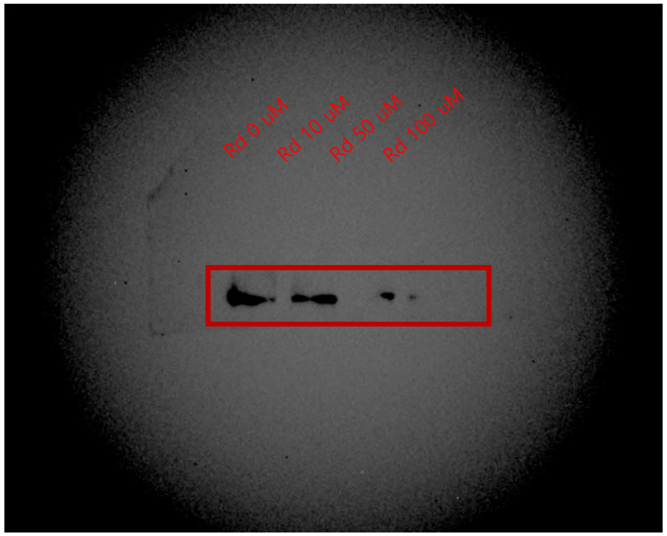

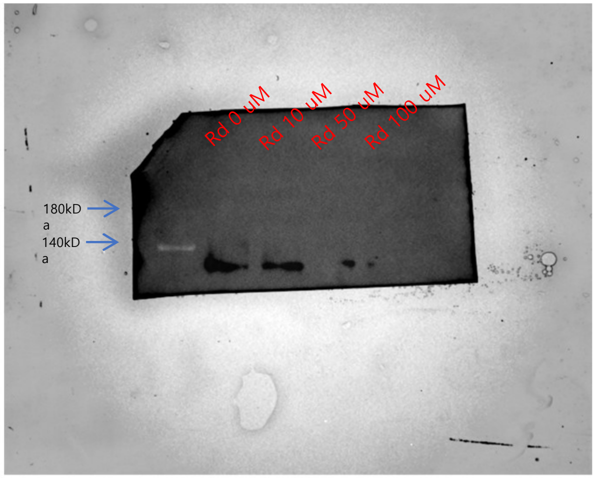

MM.1S 20200220 JAK2 (CST-3230) 1:500 dilution with 5% skim milk in PBST

2nd ab: Rabbit

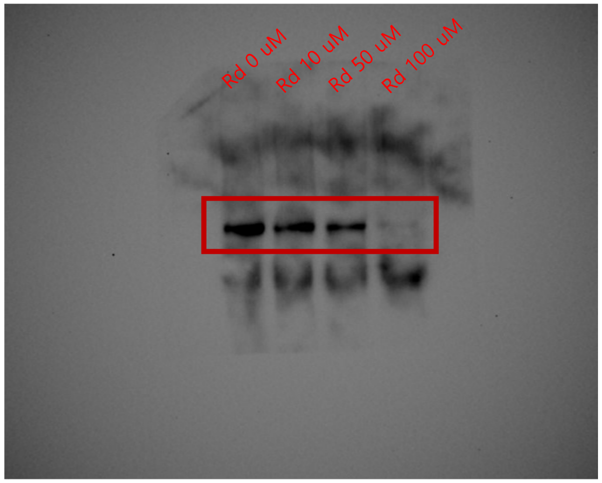

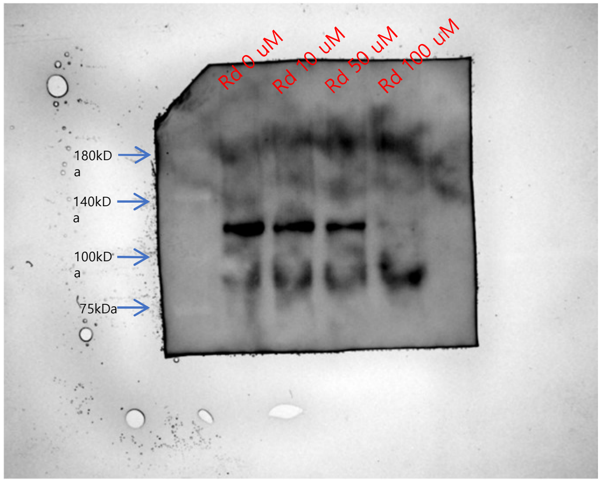

MM.1S 20200909 β-actin(SC-47778) 1:200 dilution with 5% skim milk in PBST

2nd ab: Mouse

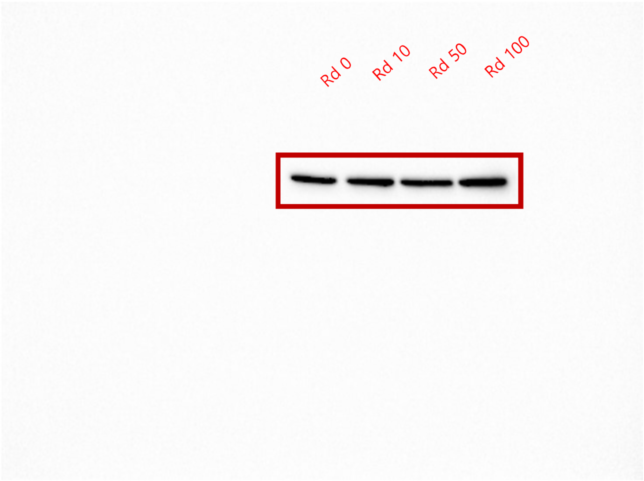

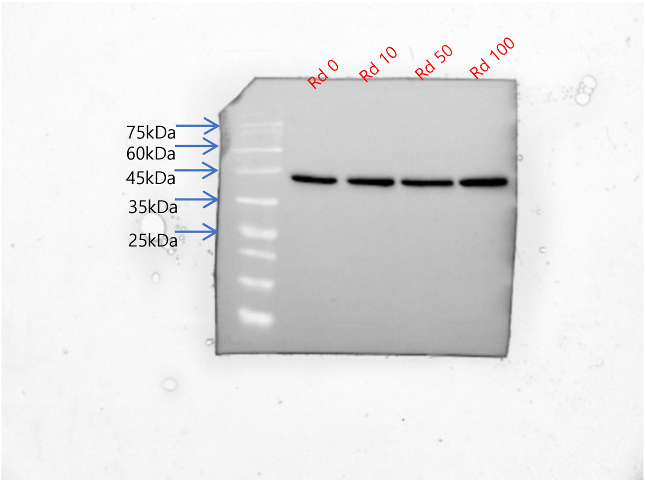

MM.1S 20200904 JAK-2(CST-3230) 1:500 dilution with 5% skim milk in PBST
2nd ab: Rabbit 125kda 8%

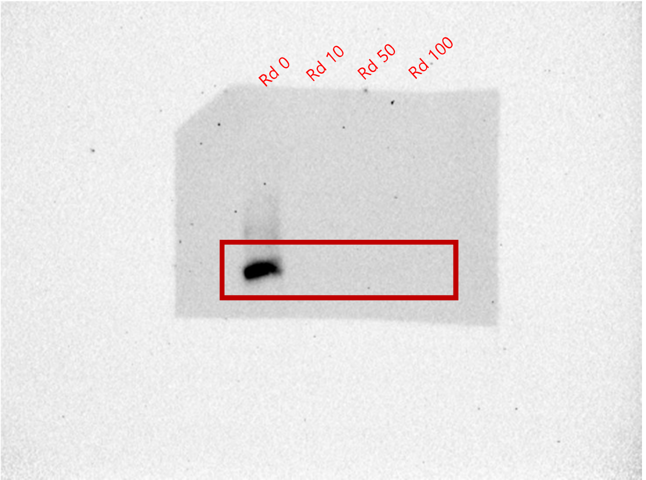

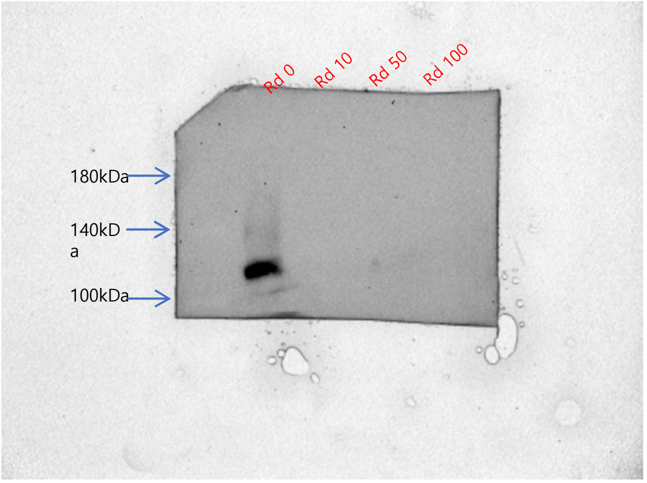

U266B1

U266B1 20201204 P-JAK2(CST-8082) 1:500 dilution with 5% skim milk in PBST

2nd ab: Rabbit

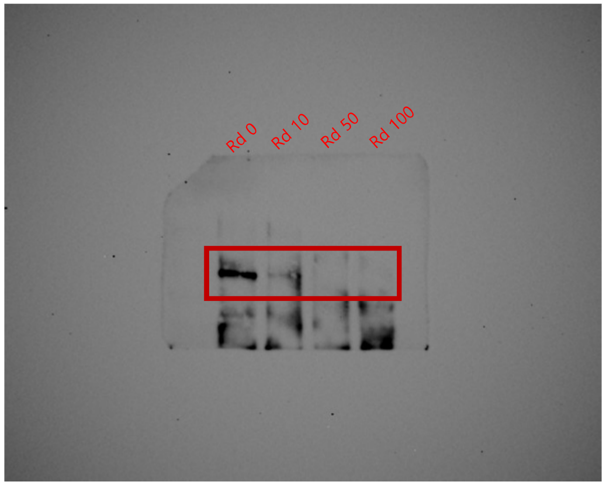

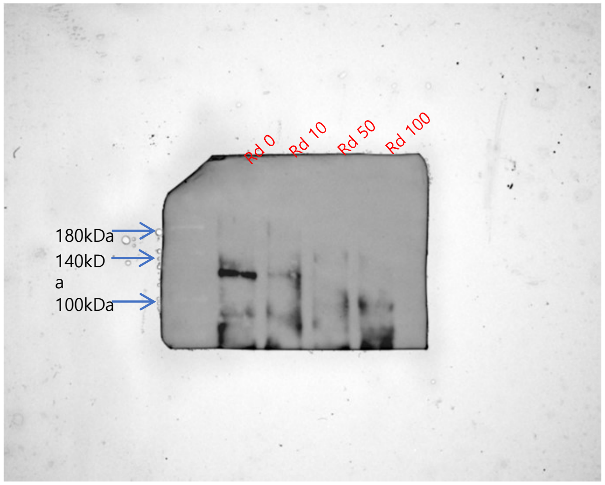

U266B1_20200507 JAK2(CST-3230) 1:500 dilution with 5% skim milk in PBST

2nd ab: Rabbit 125kda 8%

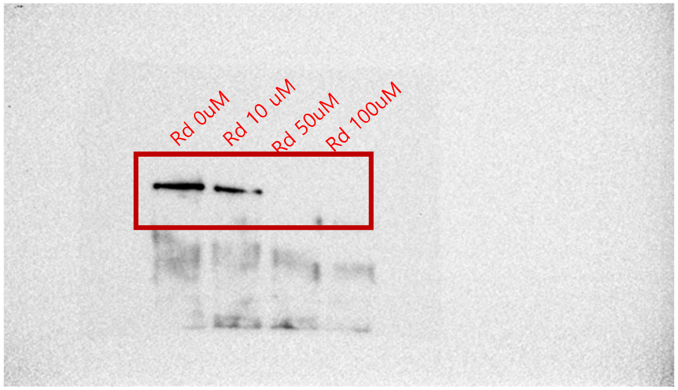

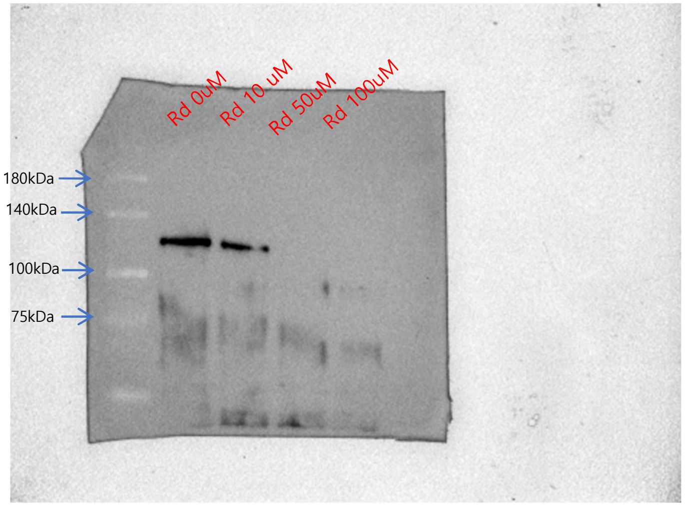

U266B1 20201215 JAK2 (CST-3230) 1:500 dilution with 5% skim milk in PBST
2nd ab: Rabbit 125Kda 8%

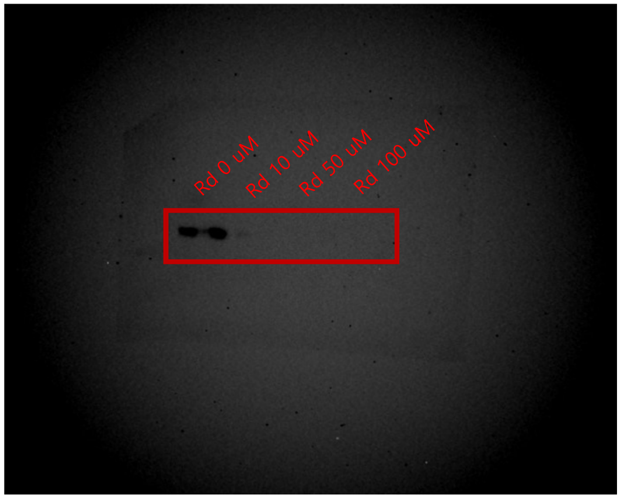

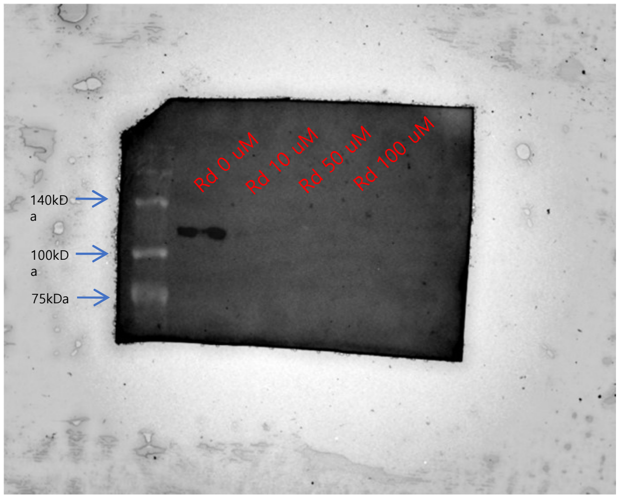

IM-9

IM-9 20201230 P-JAK2 (CST-8082) 1:500 dilution with 5% skim milk in PBST 2nd ab: Rabbit

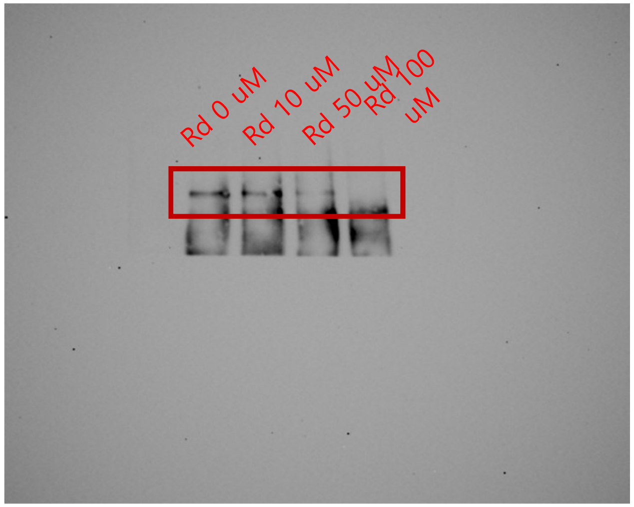

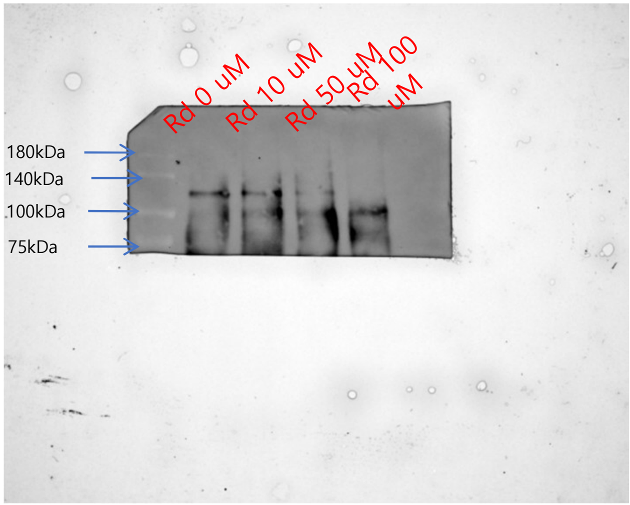

IM-9 20201224 JAK2 (CST-3230) 1:500 dilution with 5% skim milk in PBST

2nd ab: Rabbit

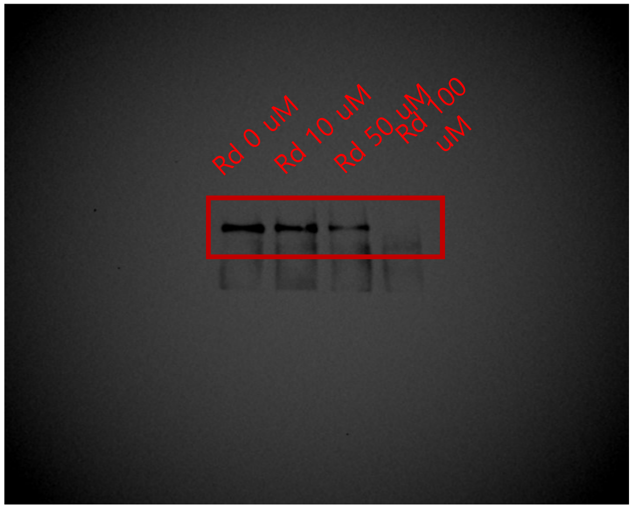

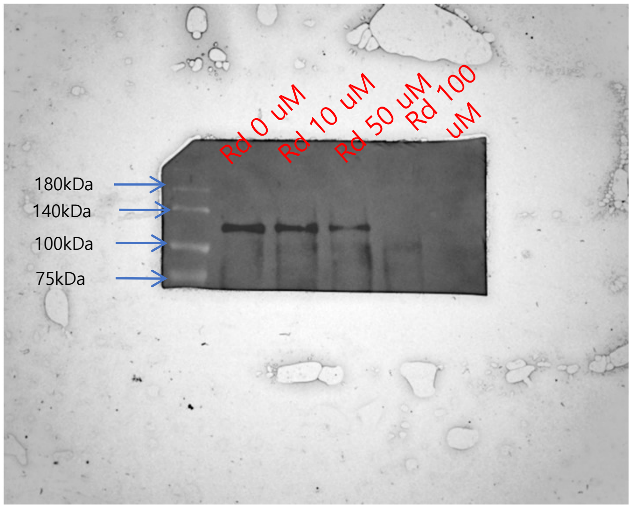

IM-9 20200902 β-actin(SC-47778) 1:200 dilution with 5% skim milk in PBST

2nd ab: Mouse

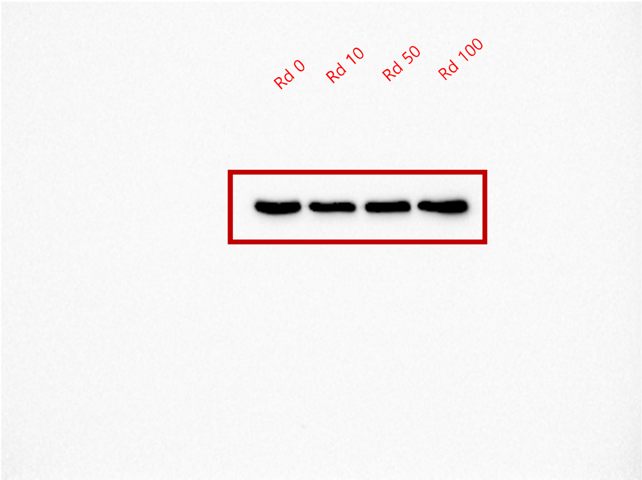

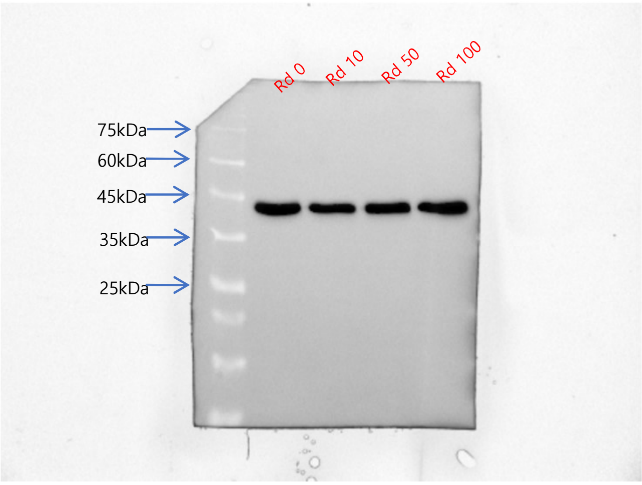

IM-9 20200902 JAK-2(CST-3230) 1:500 dilution with 5% skim milk in PBST

2nd ab: Rabbit

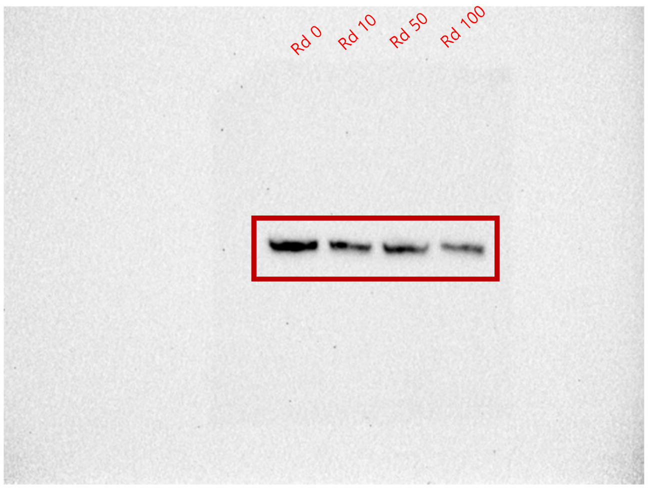

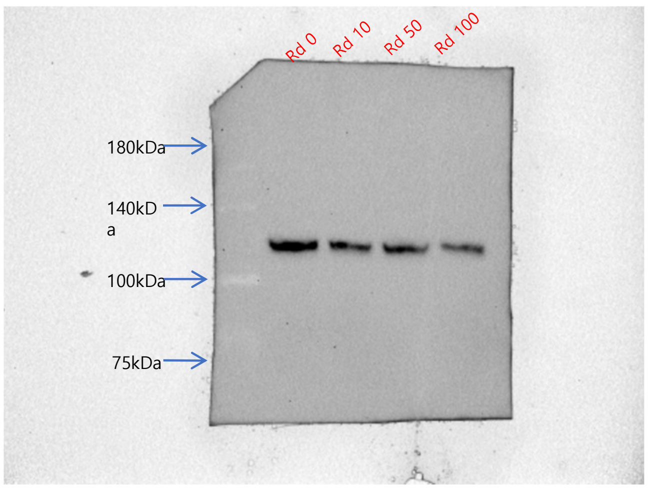

Supplement: S1 Raw images — (PDF) [file pone.0265958.s004.pdf]
