# Supplementary material for: Radotinib inhibits multiple myeloma cell proliferation via suppression of STAT3 signaling
Source: PLoS One. 2022 May 3;17(5):e0265958. doi: 10.1371/journal.pone.0265958 (PMC9064077; doi:10.1371/journal.pone.0265958)

Fig. 3C. Heo et al

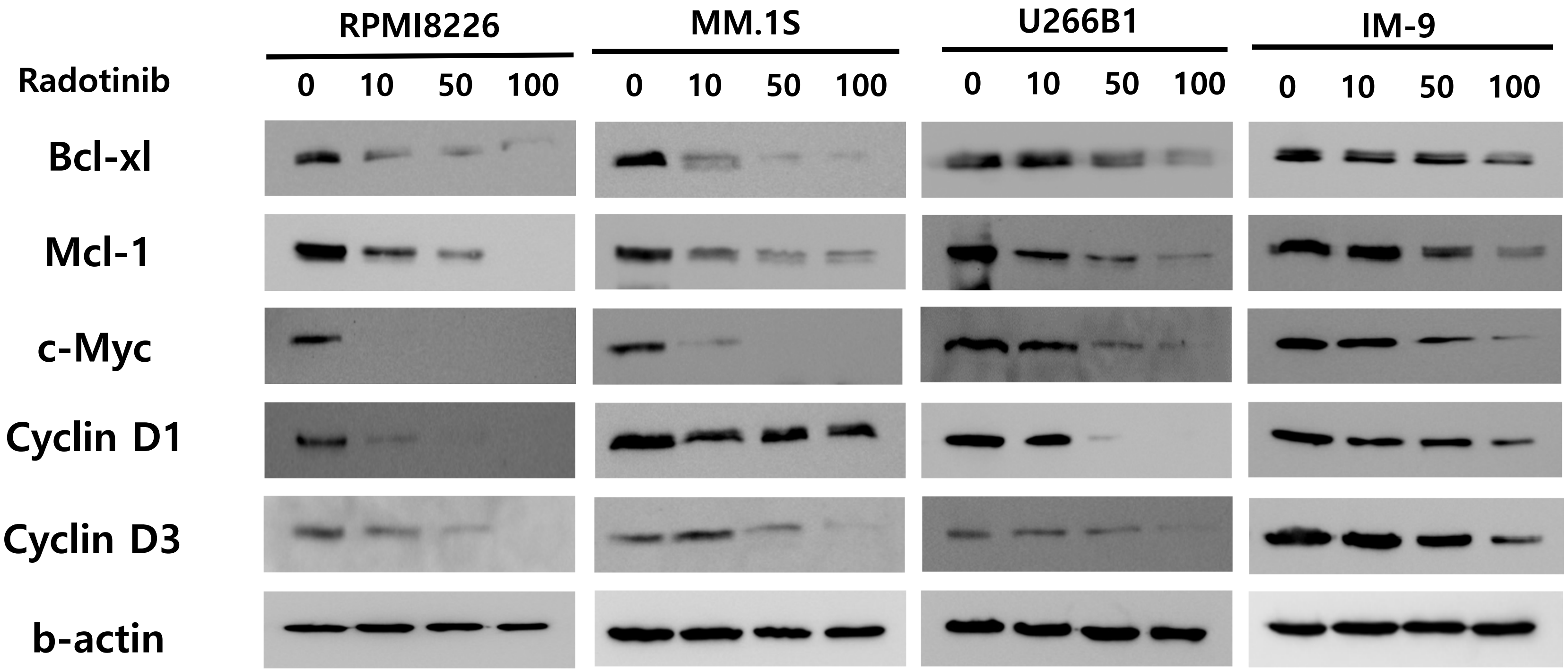

RPMI8226

RPMI8226 20180314 BCL-XL (CST-2764) 1:500 dilution with 5% skim milk in PBST

2nd ab: Rabbit

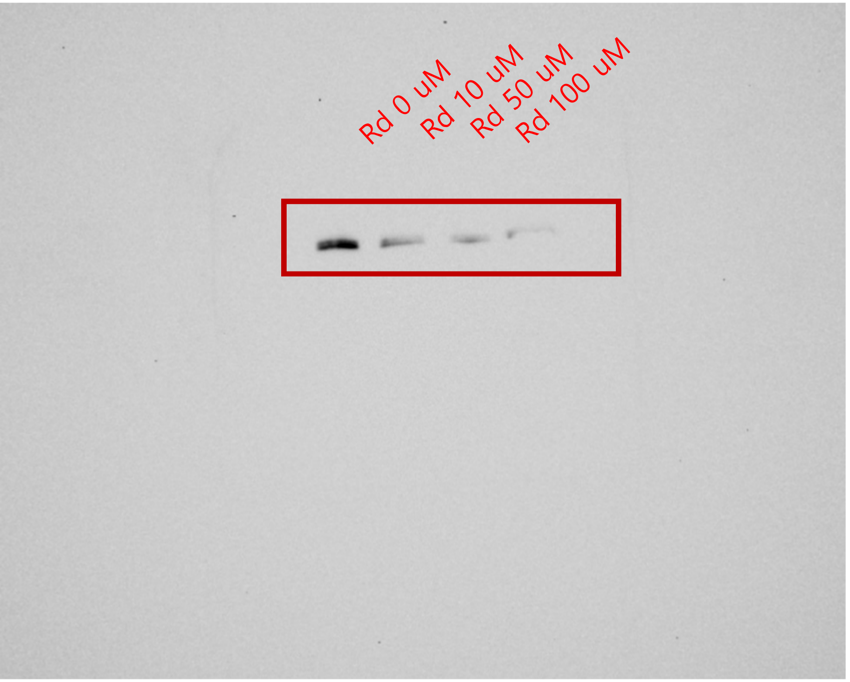

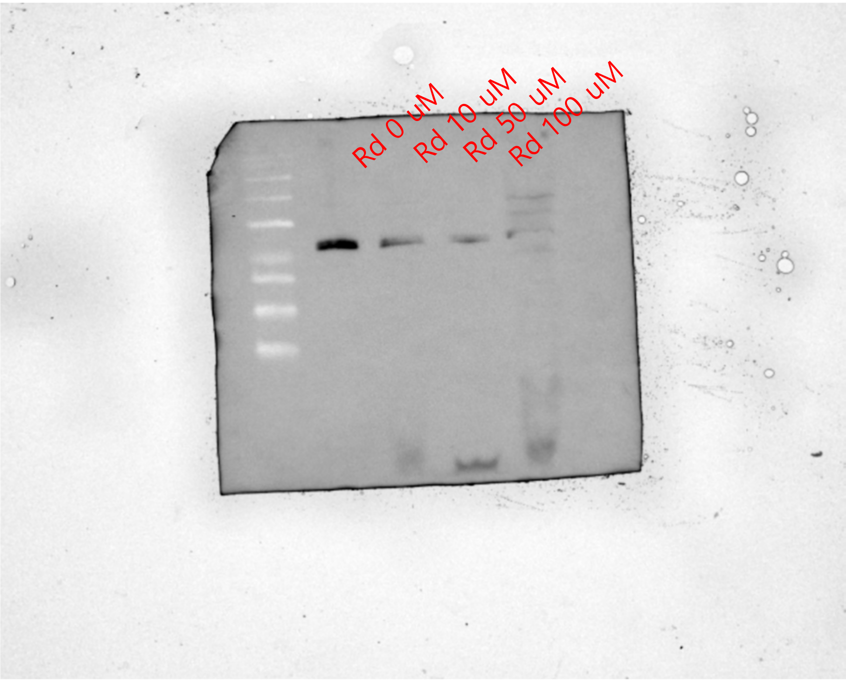

RPMI8226 20190322 MCL-1(CST-5453) 1:500 dilution with 5% skim milk in PBST
2nd ab: Rabbit

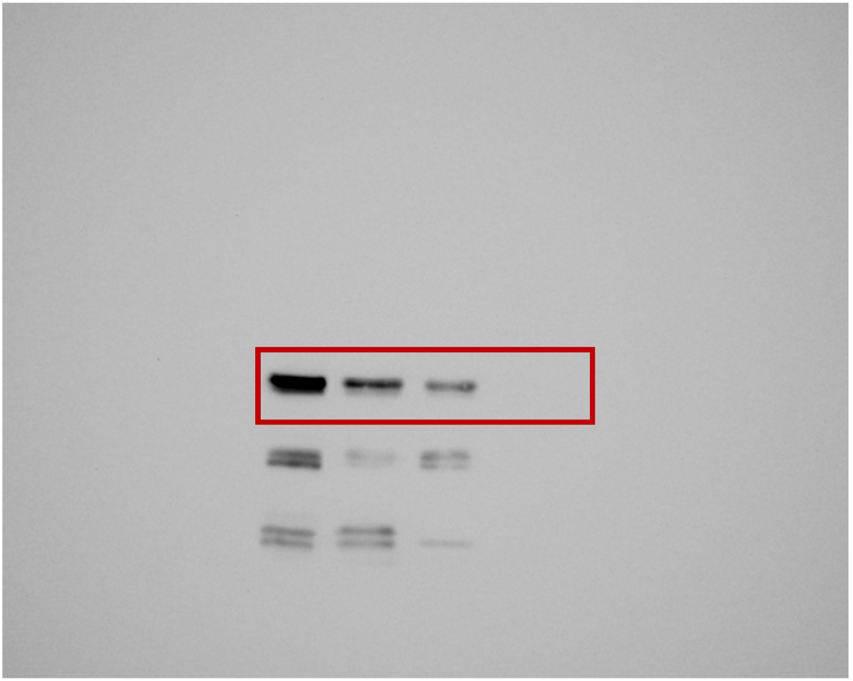

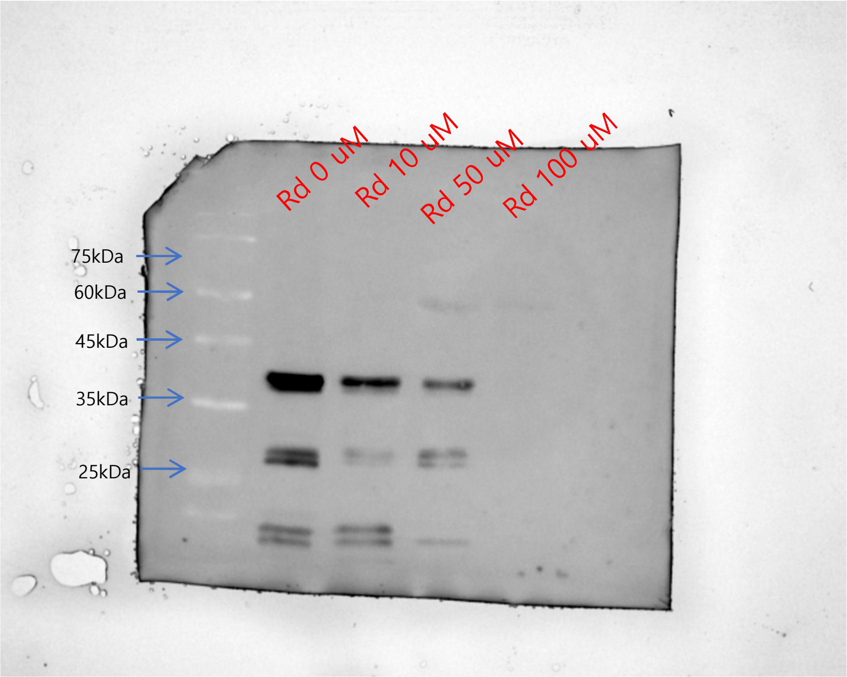

RPMI8226 20180620 C-myc (CST-5605) 1:500 dilution with 5% skim milk in PBST
2nd ab: Rabbit

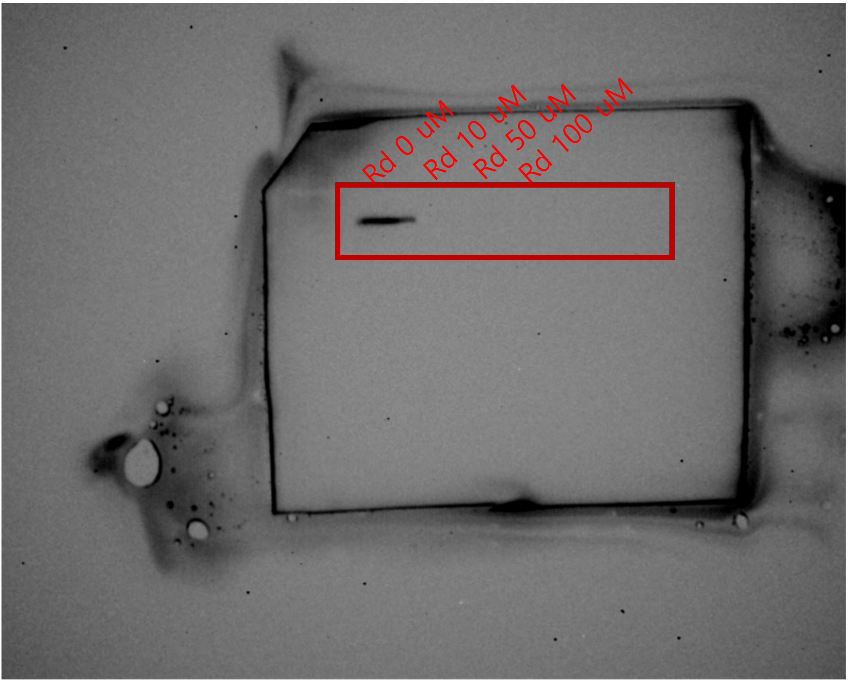

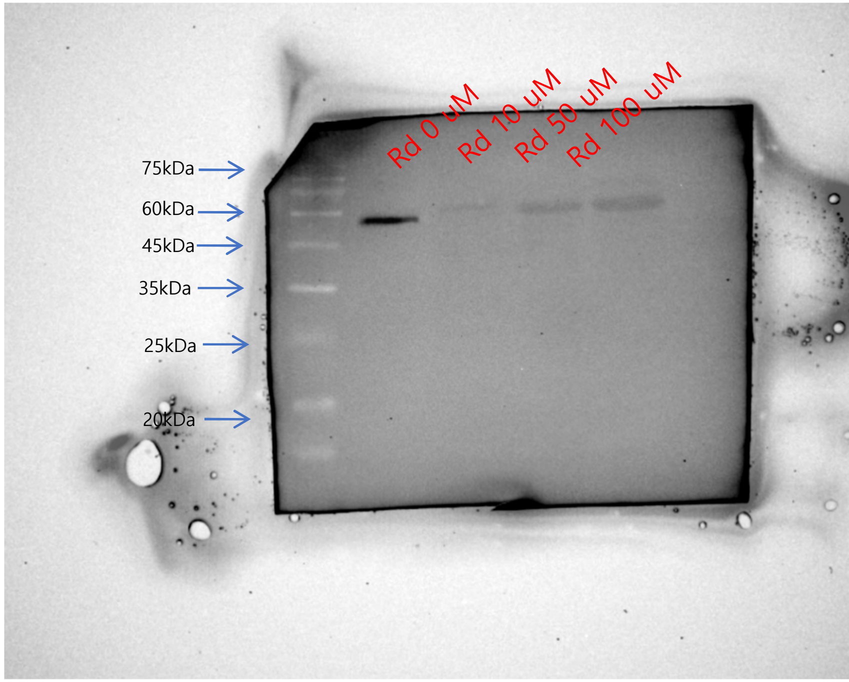

RPMI8226 20190327 Cyclin D1(CST-2978) 1:500 dilution with 5% skim milk in PBST
2nd ab: Rabbit

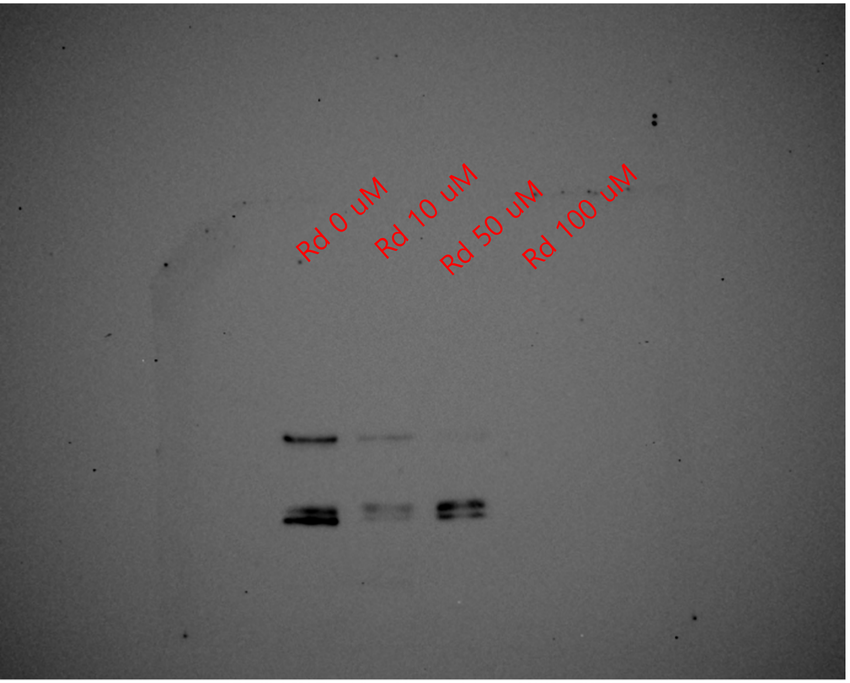

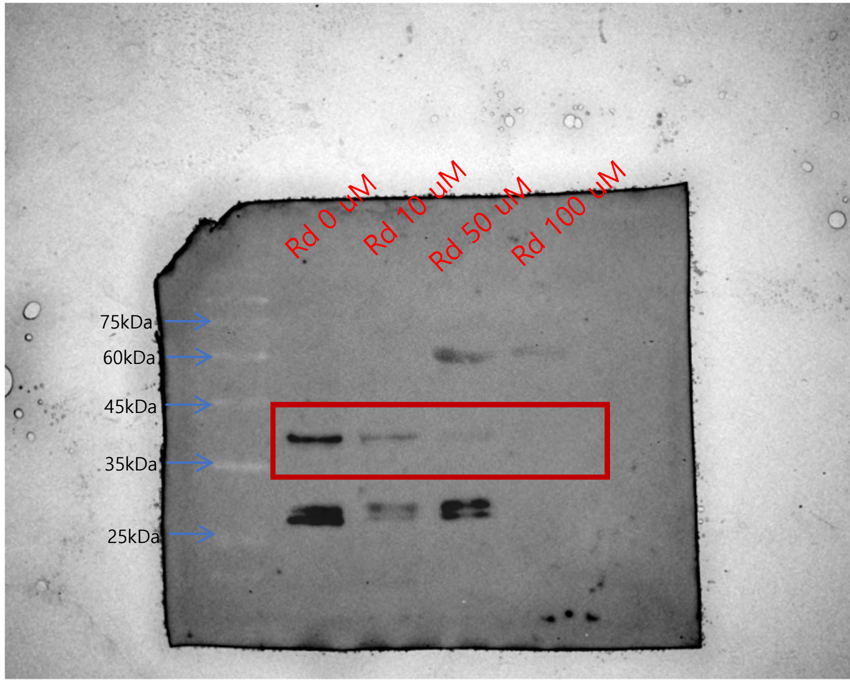

RPMI8226 20190320 Cyclin D3(CST-2936) 1:500 dilution with 5% skim milk in PBST
2nd ab: Mouse

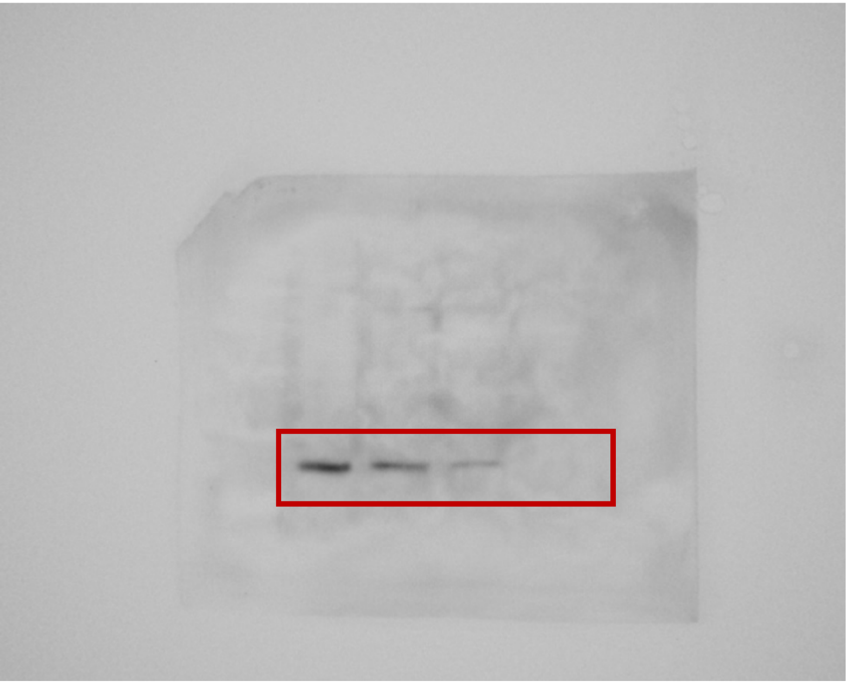

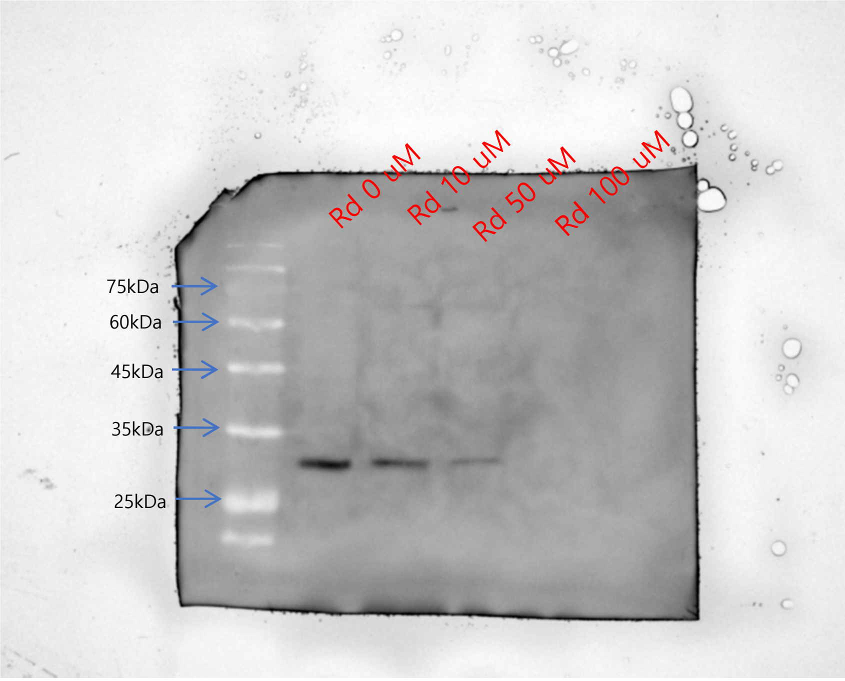

RPMI8226 20180406 B-actin(SC-47778) 1:200 dilution with 5% skim milk in PBST
2nd ab: Mouse

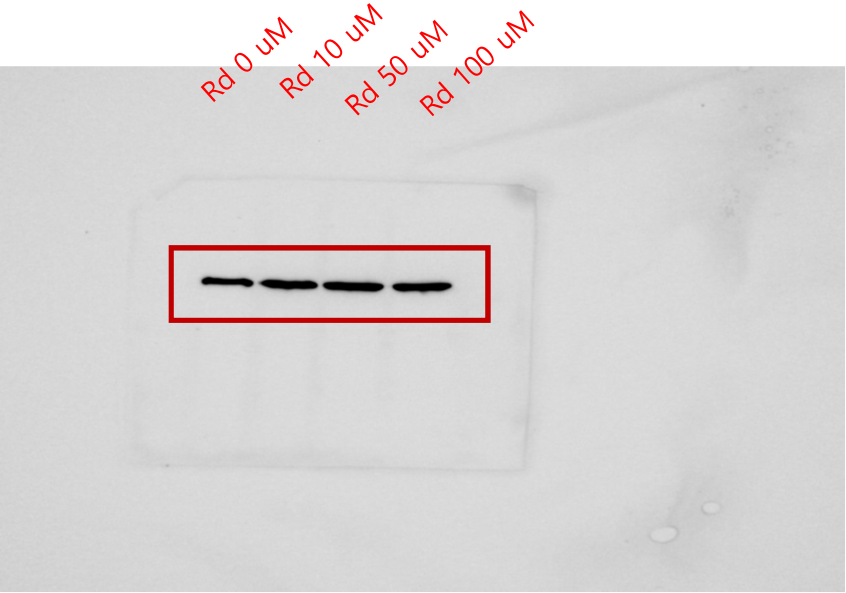

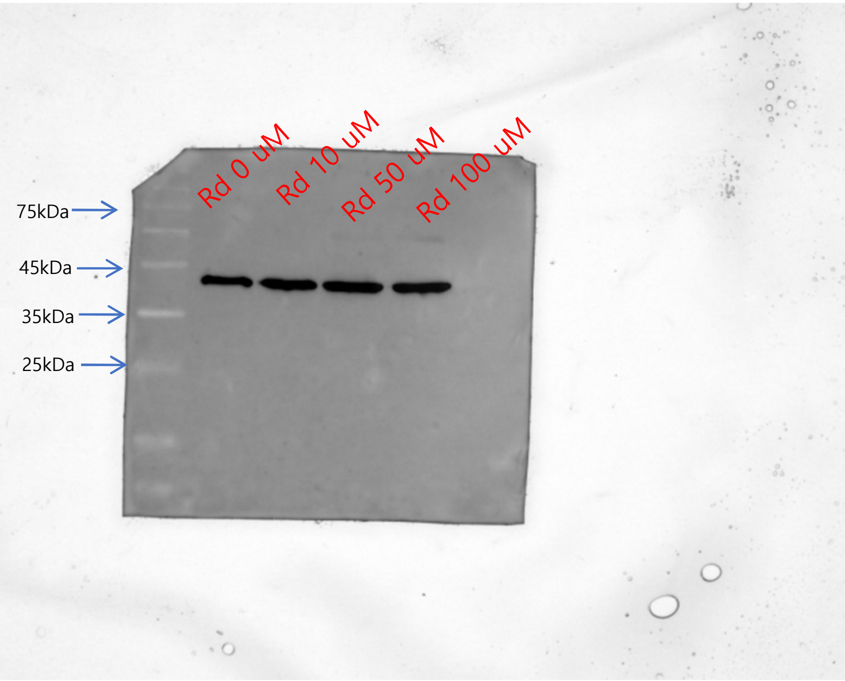

MM.1S

MM.1S 20180525 BCL-XL (SC-8392) 1:200 dilution with 5% skim milk in PBST
2nd ab: Mouse

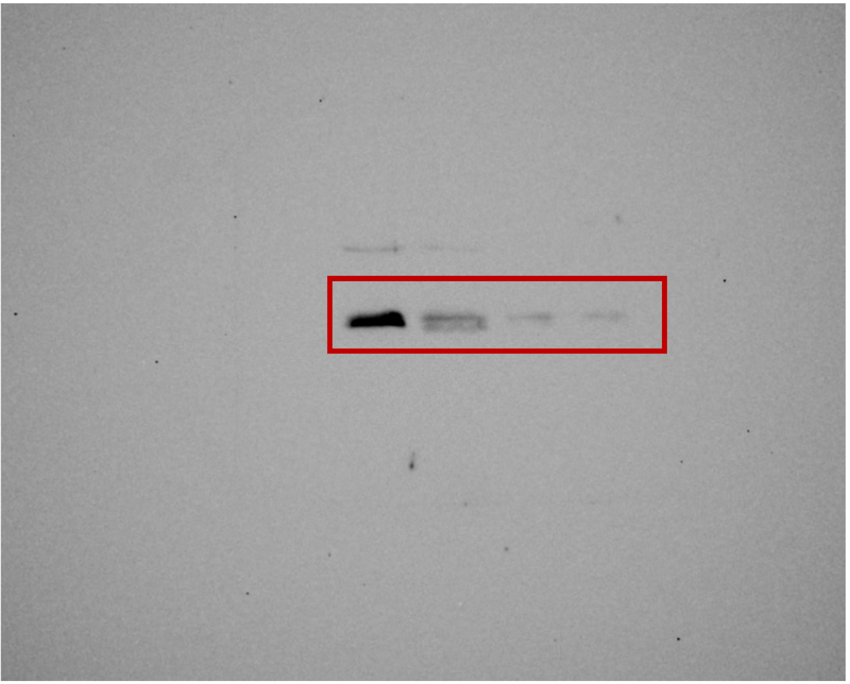

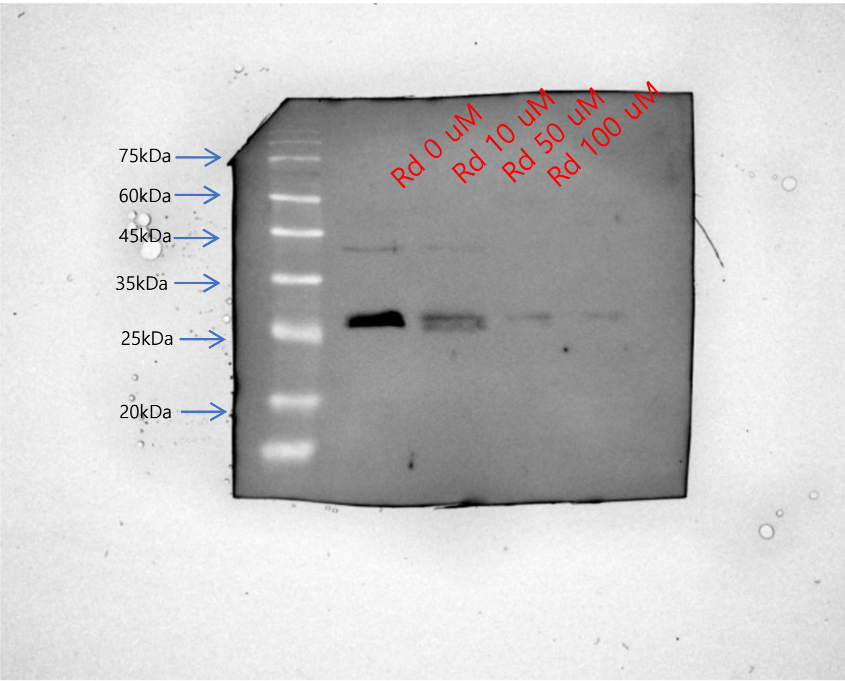

MM.1S 20181213 MCL-1(CST-5453) 1:500 dilution with 5% skim milk in PBST
2nd ab: Rabbit

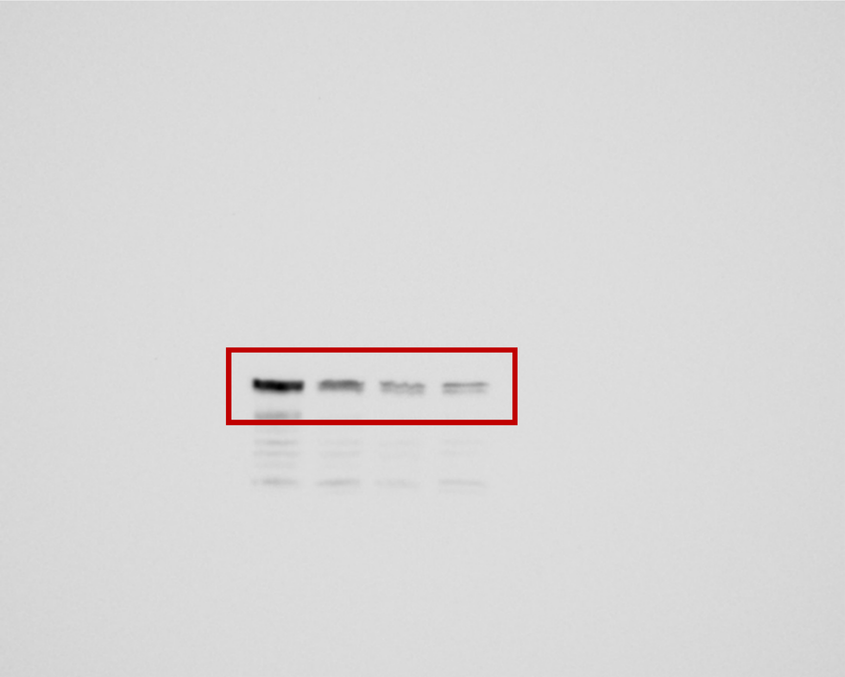

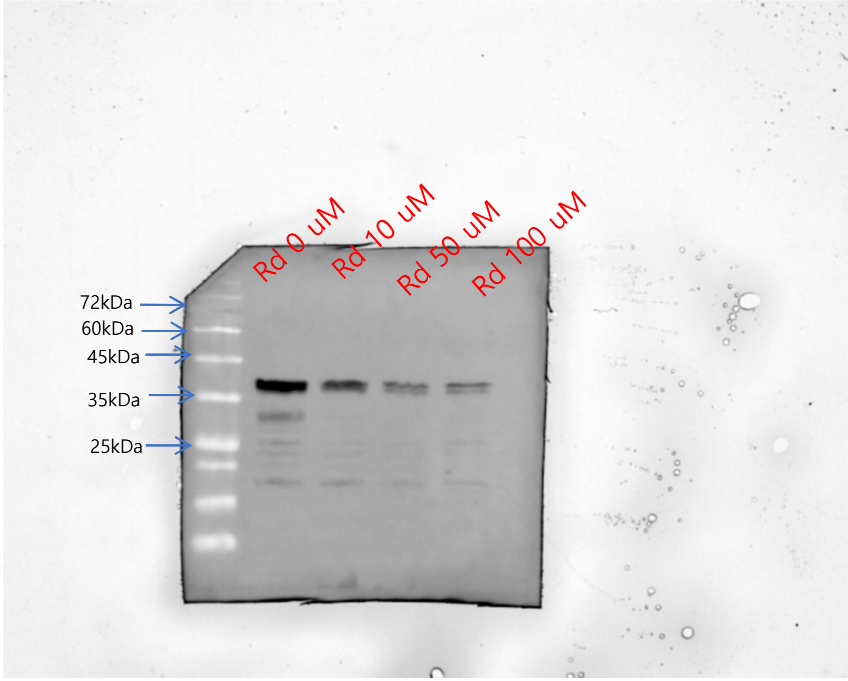

MM.1S 20180810 C-myc (CST-5605) 1:500 dilution with 5% skim milk in PBST
2nd ab: Rabbit

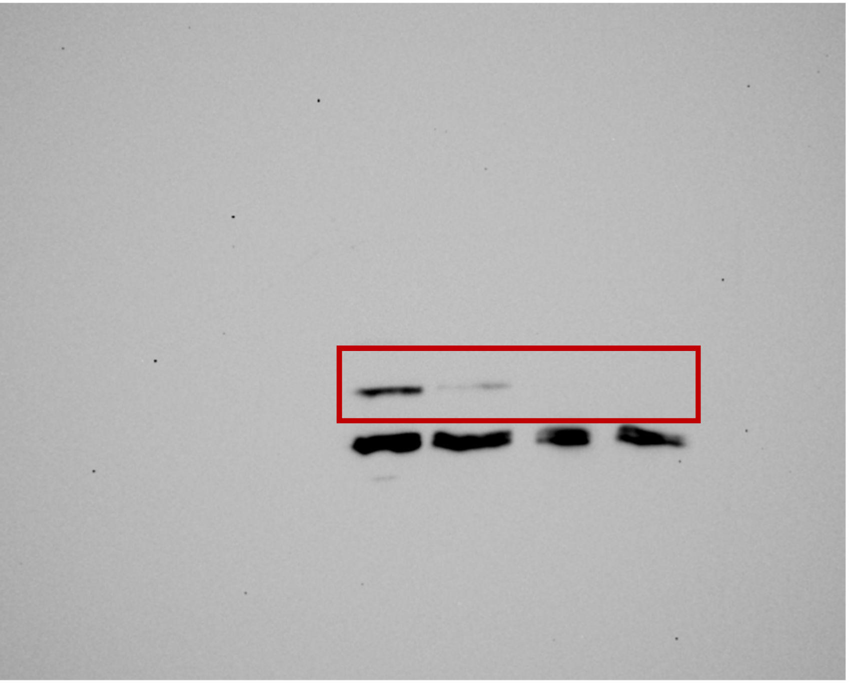

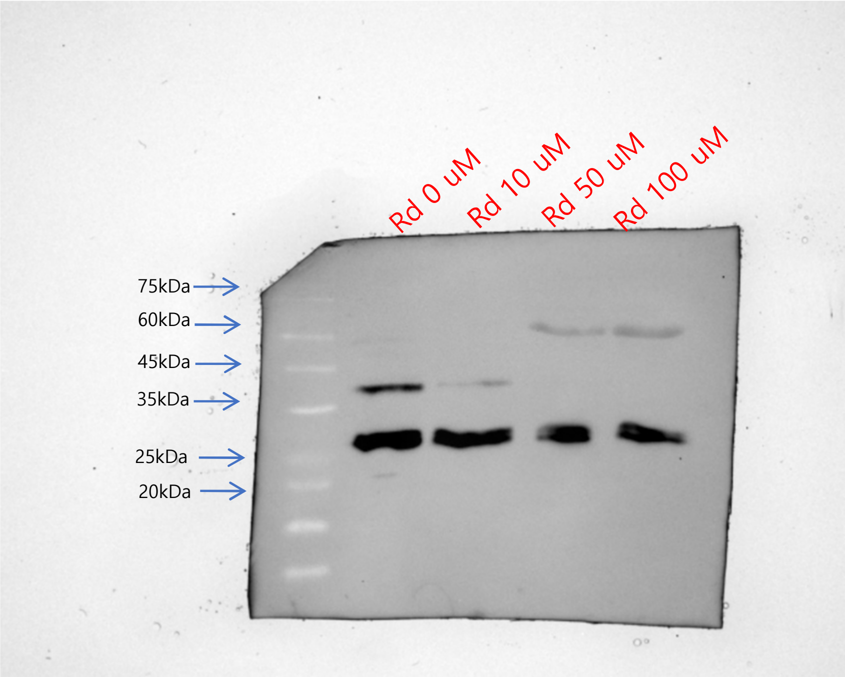

MM.1S 20180703 CyclinD1 (CST-2978) 1:500 dilution with 5% skim milk in PBST
2nd ab: Rabbit

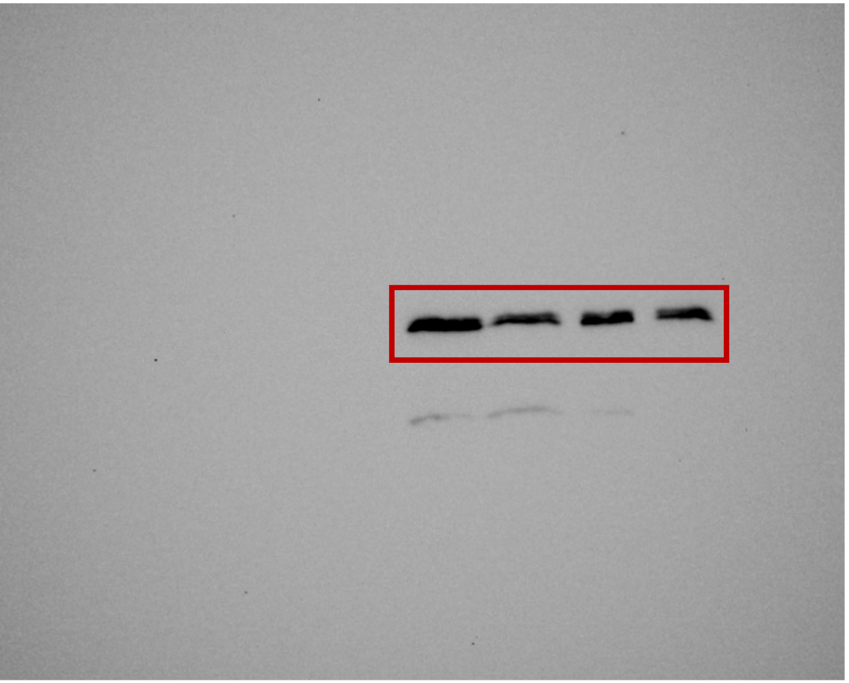

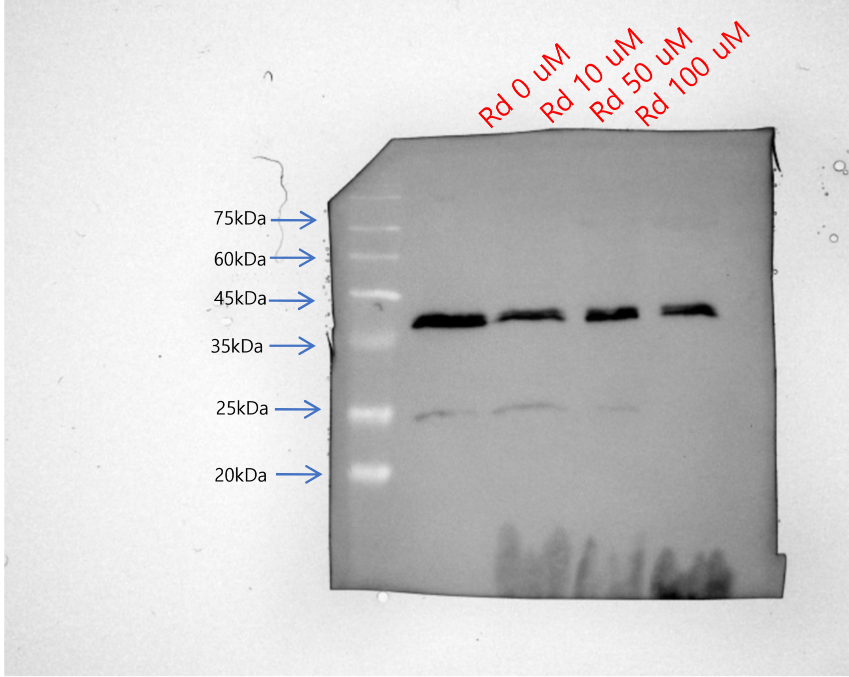

MM.1S 20181102 Cyclin D3(CST-2936) 1:500 dilution with 5% skim milk in PBST
2nd ab: Mouse

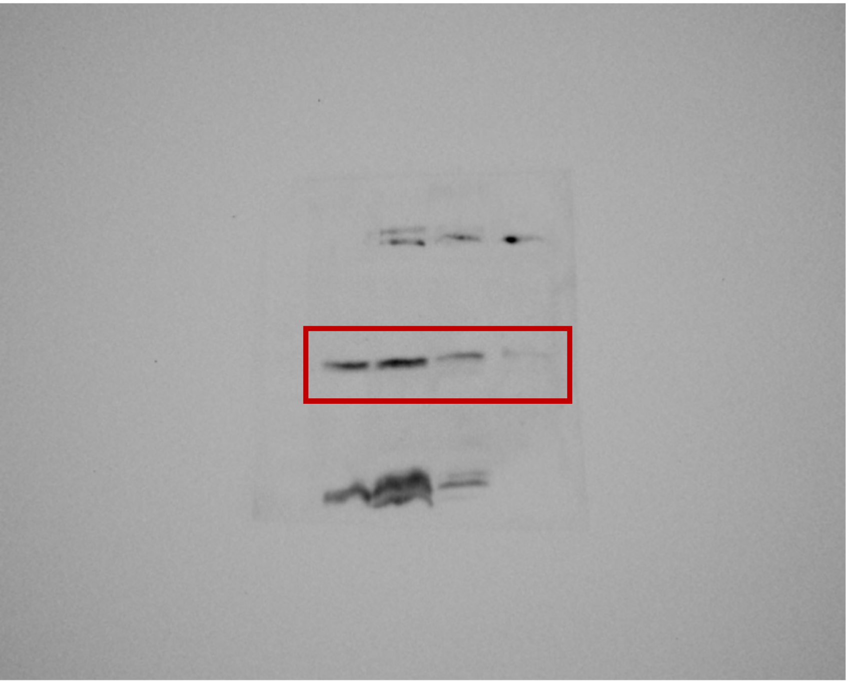

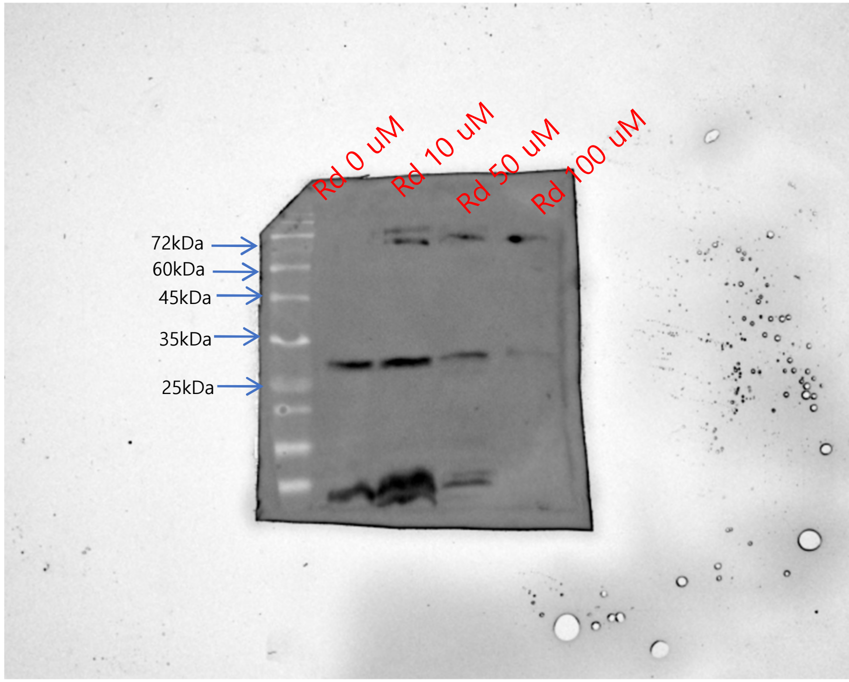

MM.1S 20181219 β-actin(SC-47778) 1:200 dilution with 5% skim milk in PBST
2nd ab: Mouse

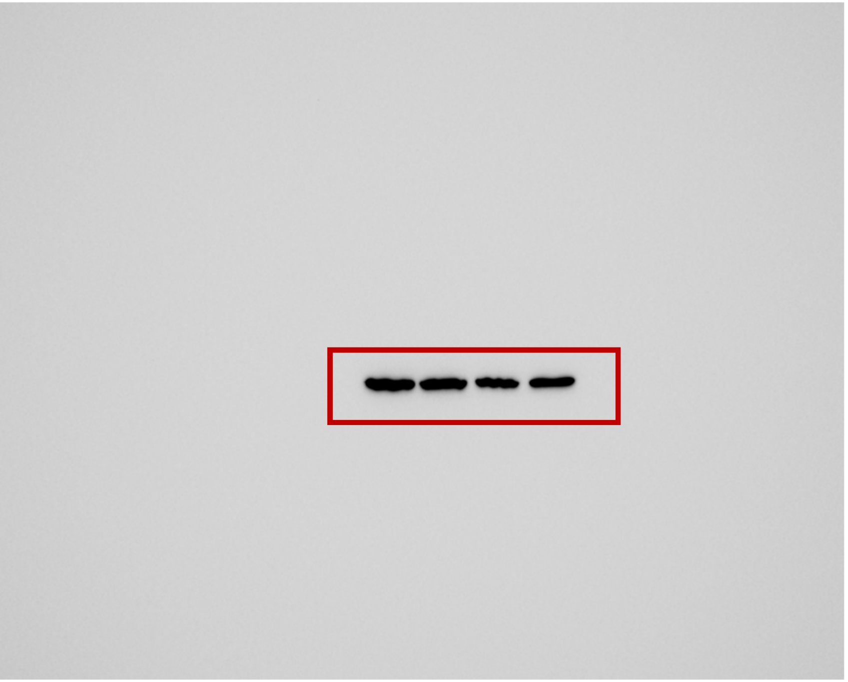

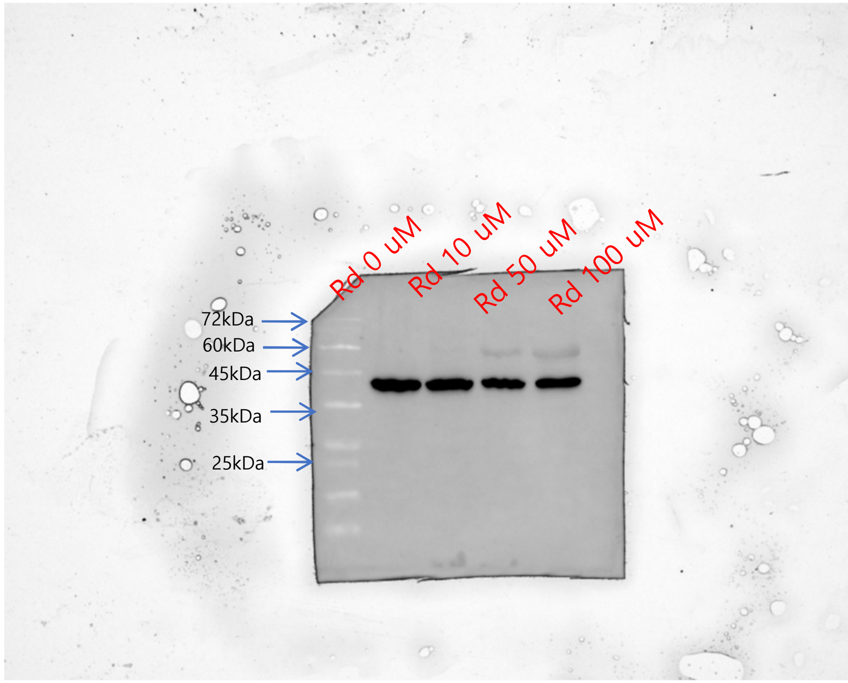

U266B1

U266B1 20190115 BCL-XL(CST-2764) 1:500 dilution with 5% skim milk in PBST
2nd ab: Rabbit

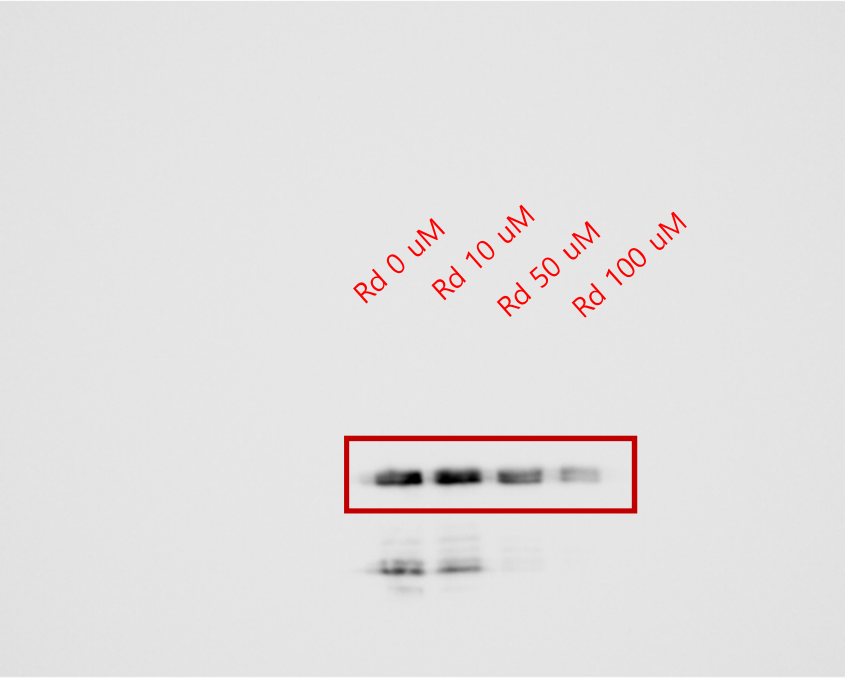

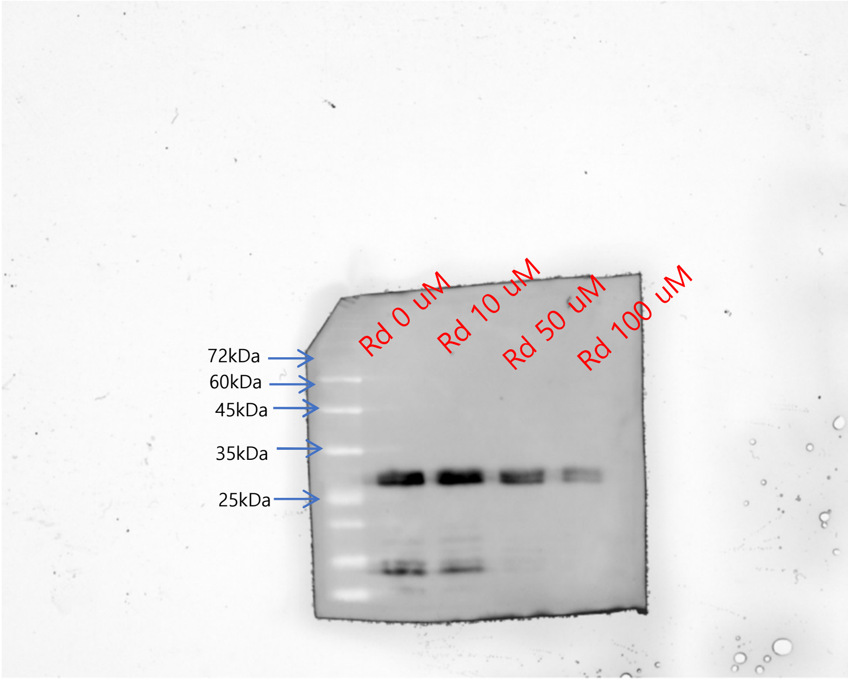

U266B1 20181122 MCL-1(CST-5453) 1:500 dilution with 5% skim milk in PBST
2nd ab: Rabbit

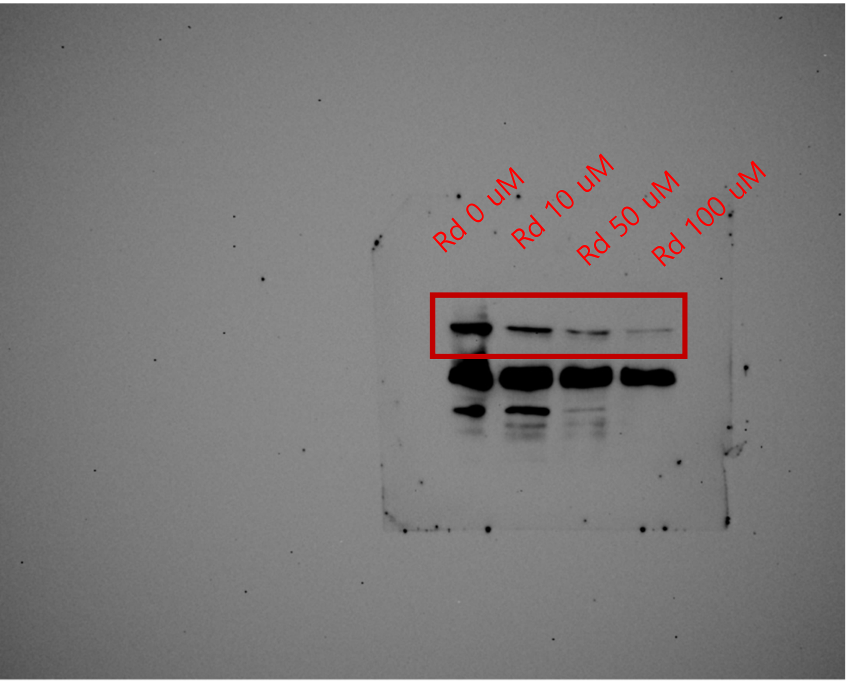

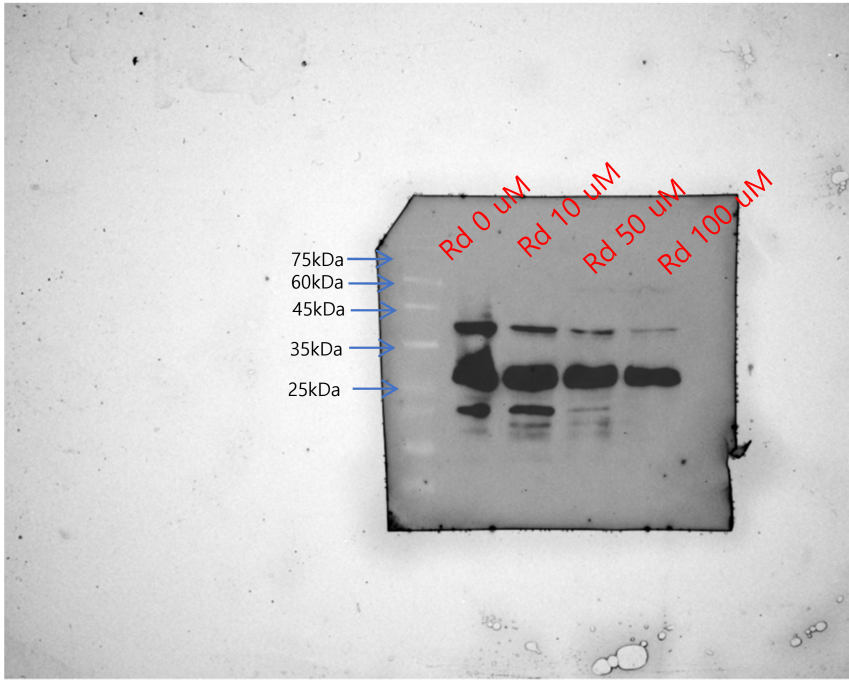

20210323 c-myc(SC-40) 1:200 dilution with 5% skim milk in PBST
2nd ab: Mouse

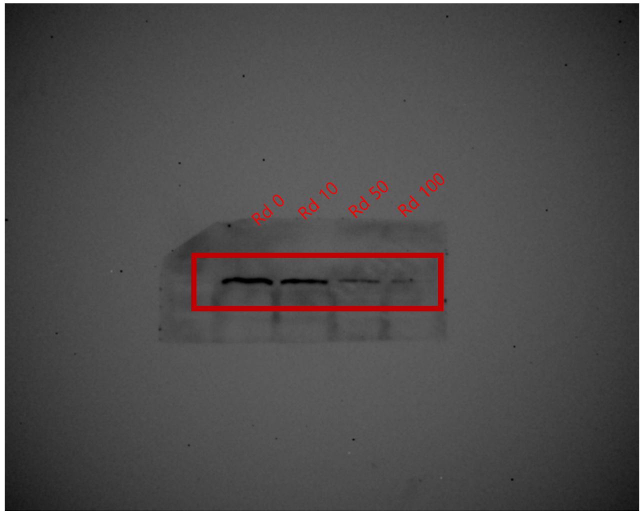

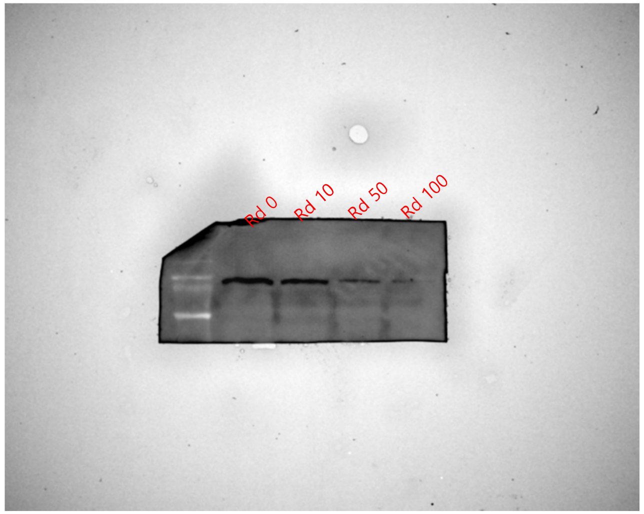

U266B1 20210316 CyclinD1(CST-2978) 1:500 dilution with 5% skim milk in PBST
2nd ab: Rabbit

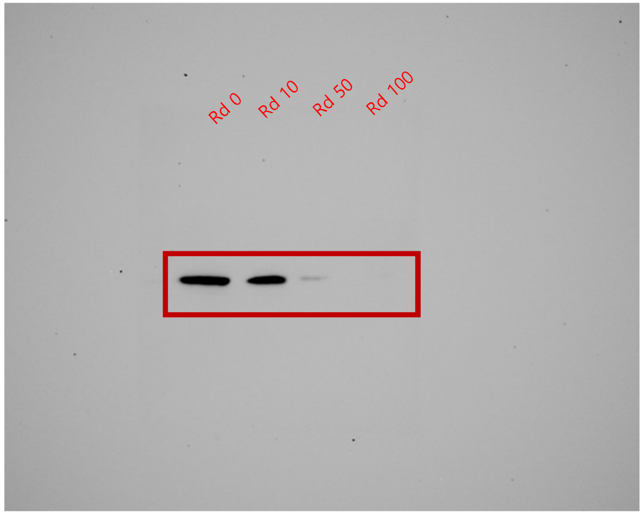

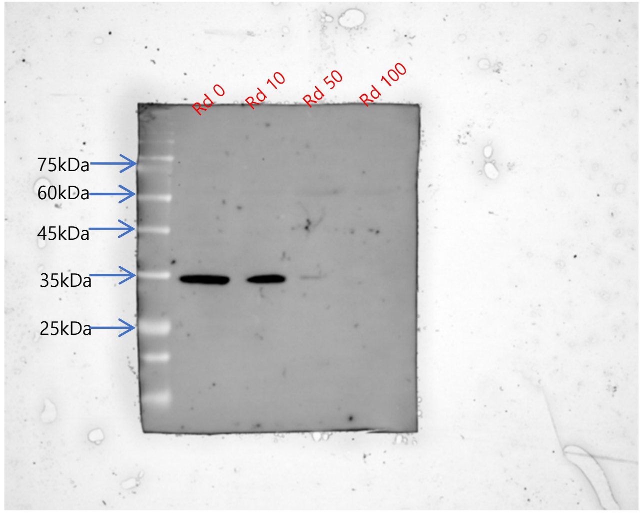

U266B1 20210316 CyclinD1(CST-2978) 1:500 dilution with 5% skim milk in PBST
2nd ab: Rabbit

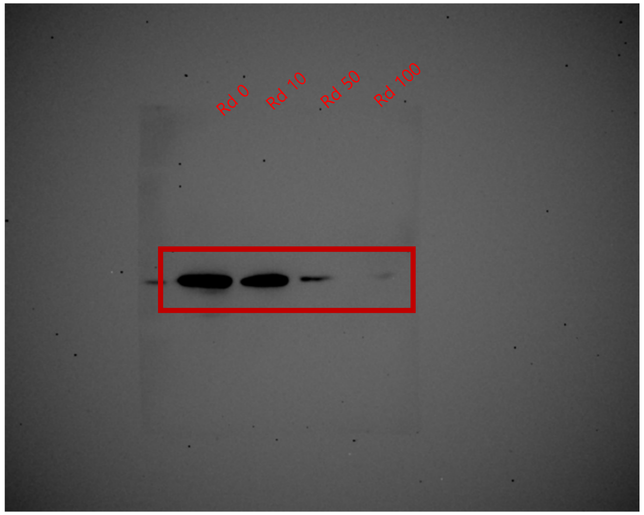

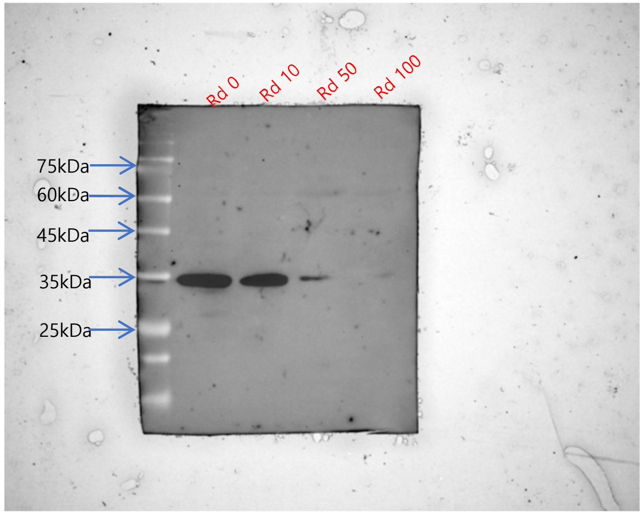

U266B1 20181116 Cyclin D3(CST-2936) 1:500 dilution with 5% skim milk in PBST
2nd ab: Mouse

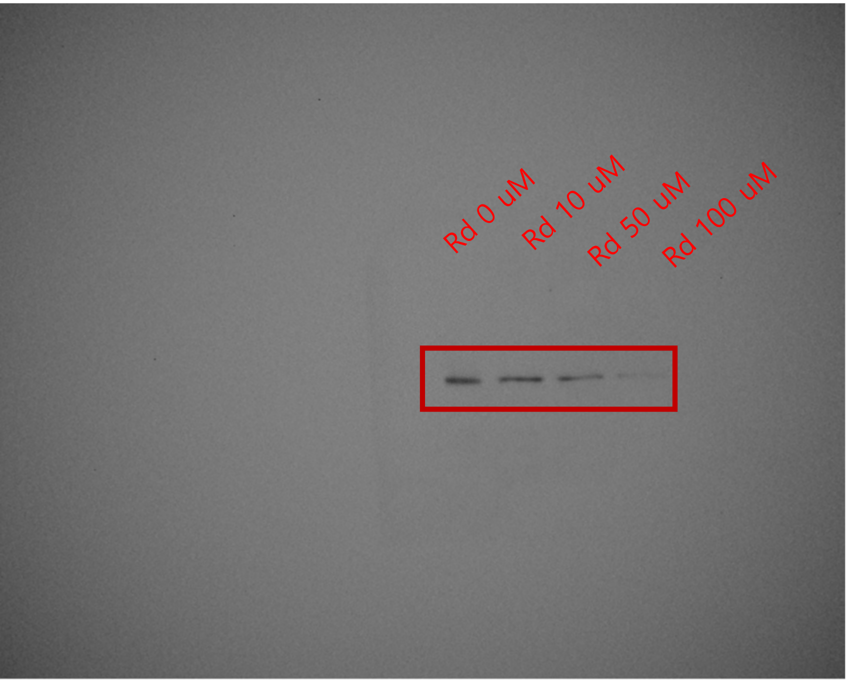

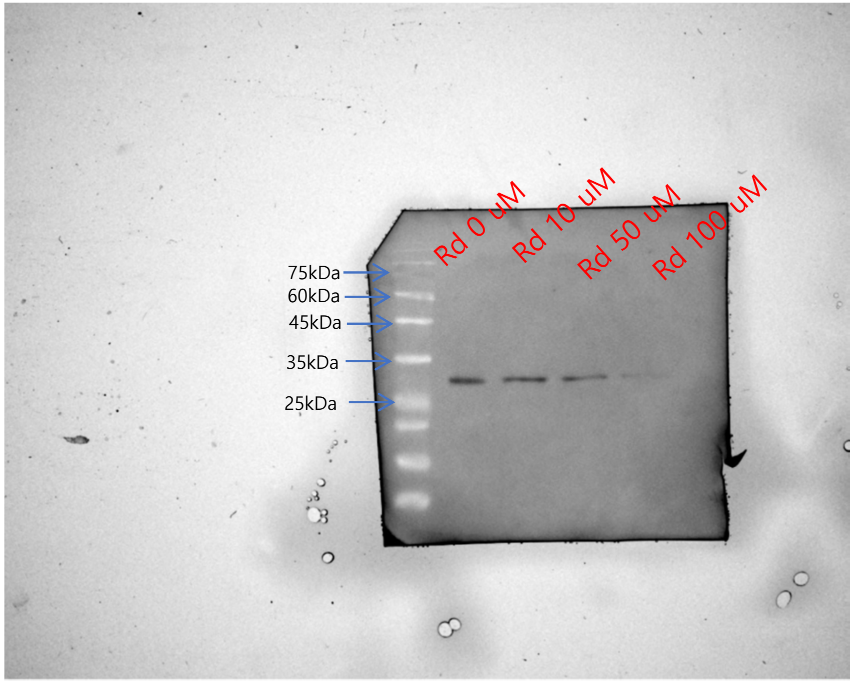

U266B1 20190424 Cyclin D3 (CST-2978) 1:500 dilution with 5% skim milk in PBST
2nd ab: Mouse

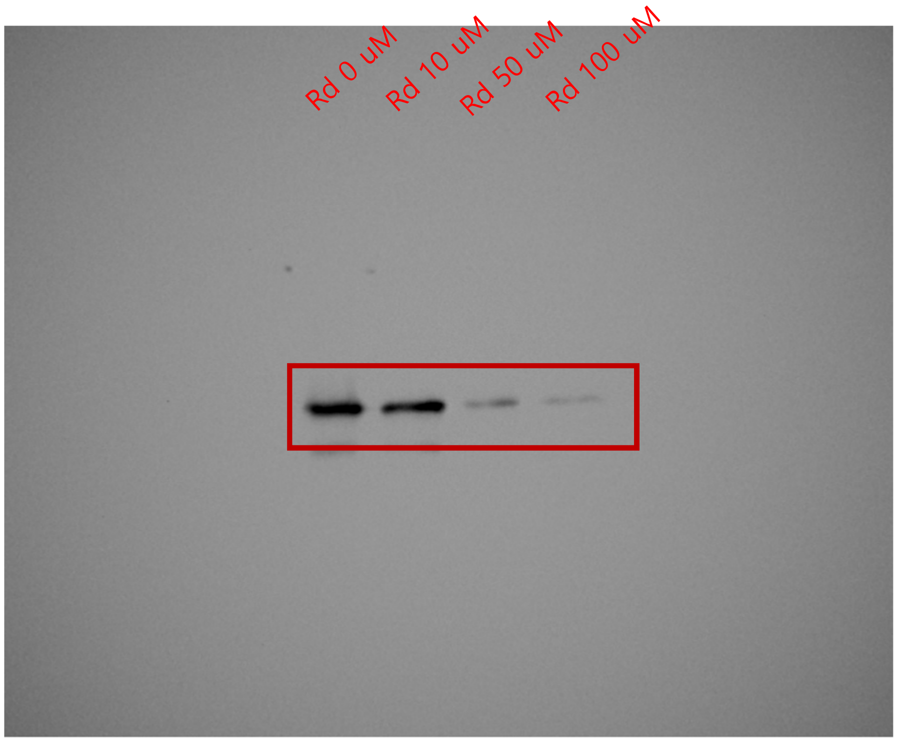

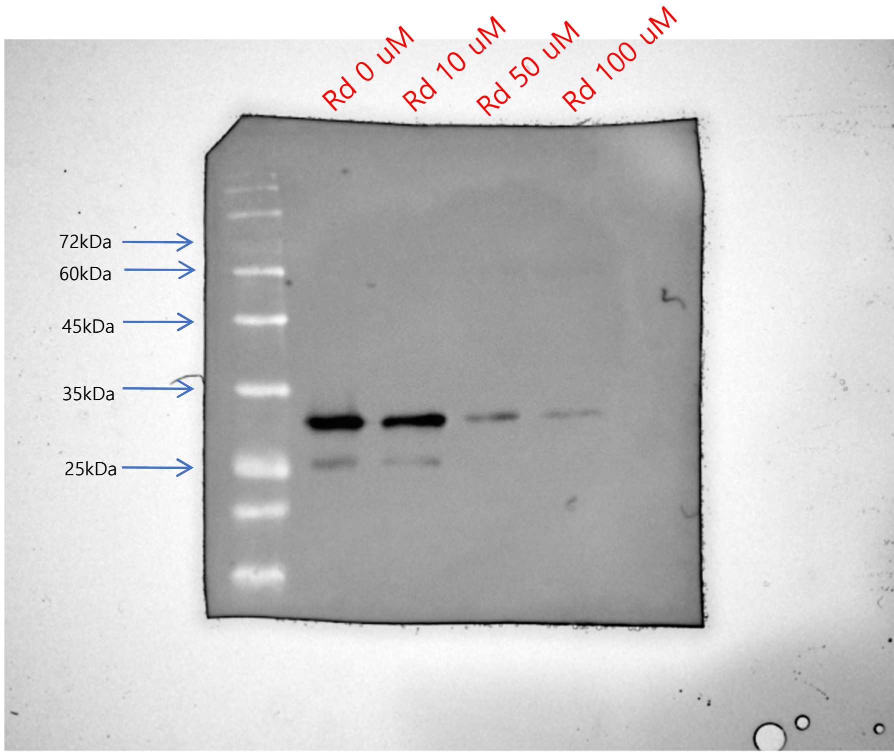

U266B1 20181127 β-actin(SC-47778) 1:00 dilution with 5% skim milk in PBST
2nd ab: Mouse

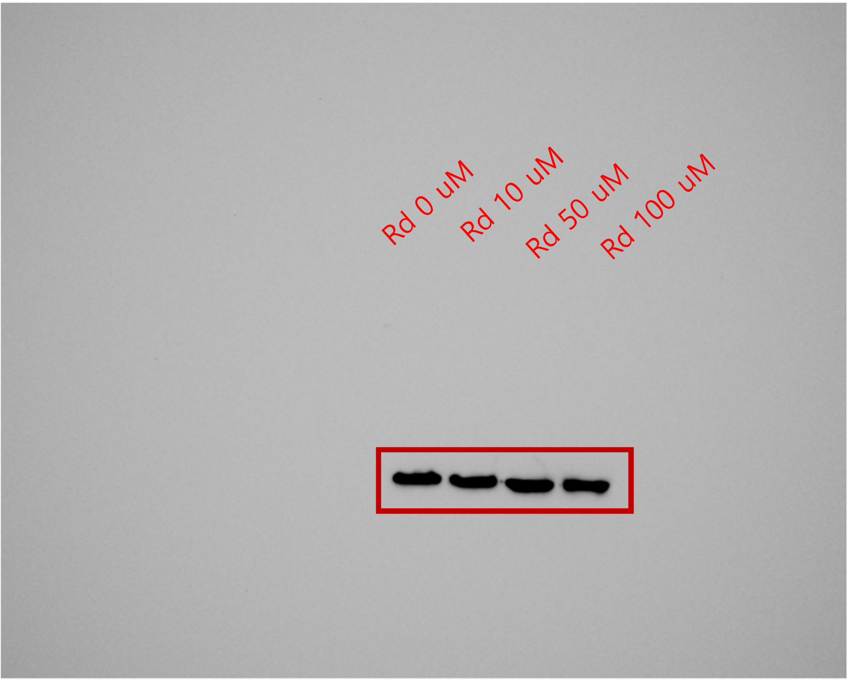

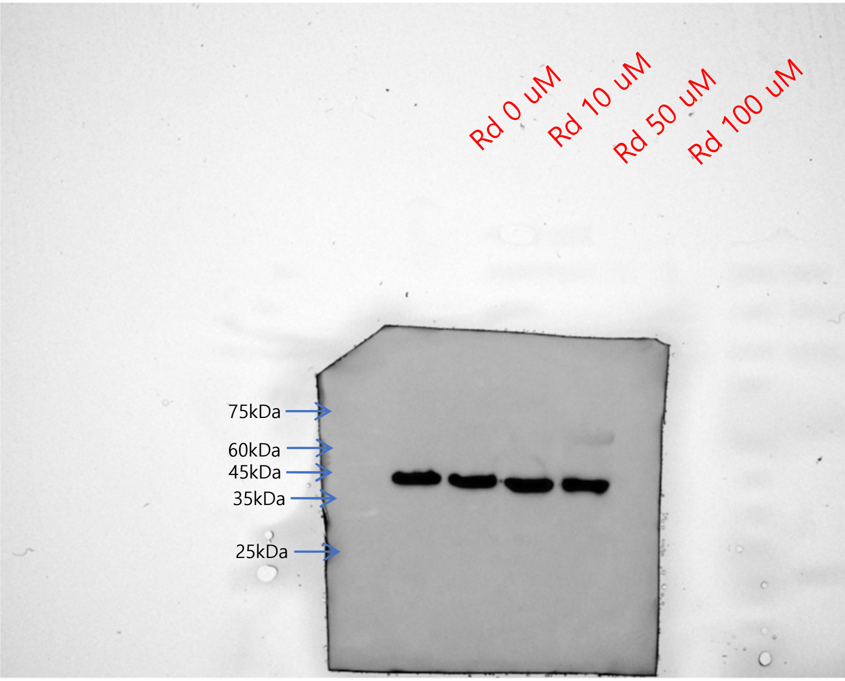

IM-9

IM-9 20180803 BCL-XL (CST-2764) 1:500 dilution with 5% skim milk in PBST
2nd ab: Rabbit

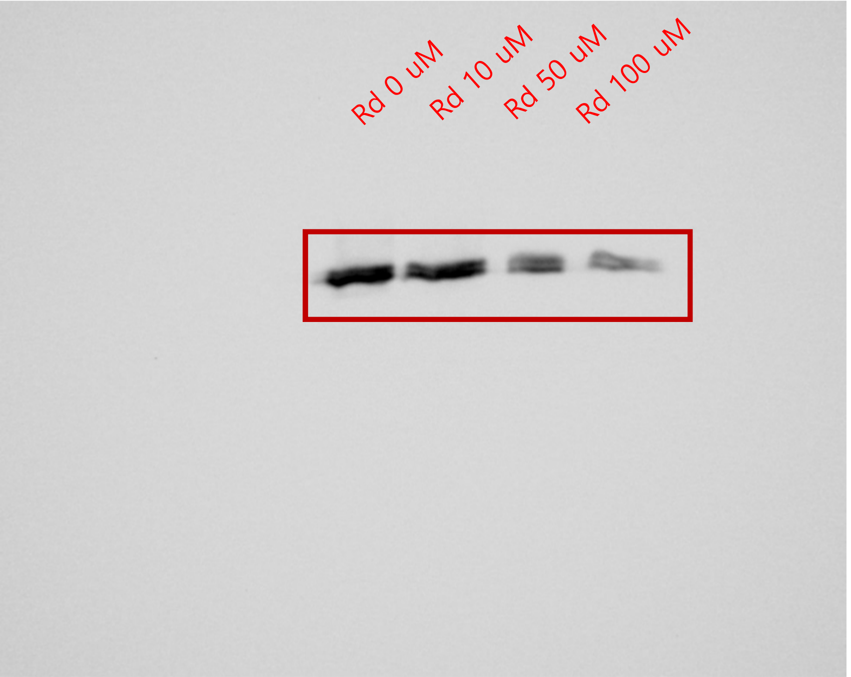

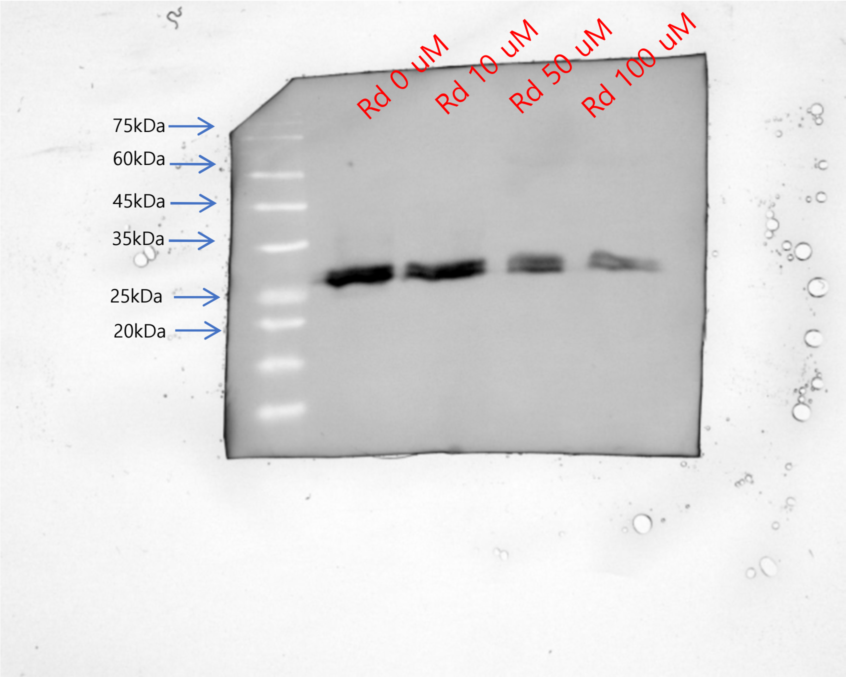

IM-9 20201209 Mcl-1(CST-5453) 1:500 dilution with 5% skim milk in PBST
2nd ab: Rabbit

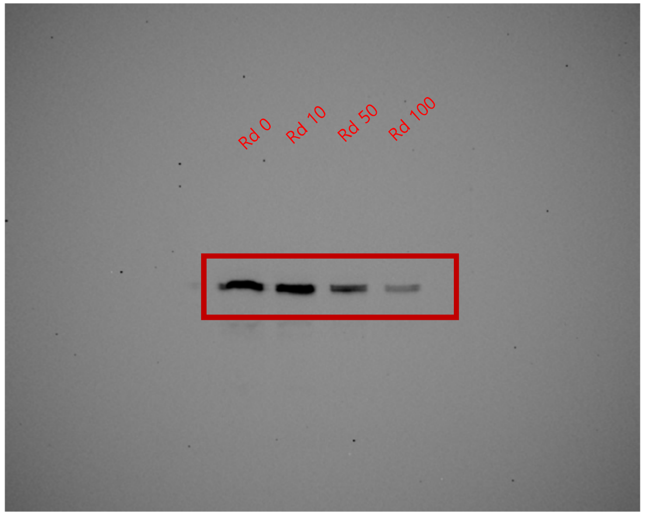

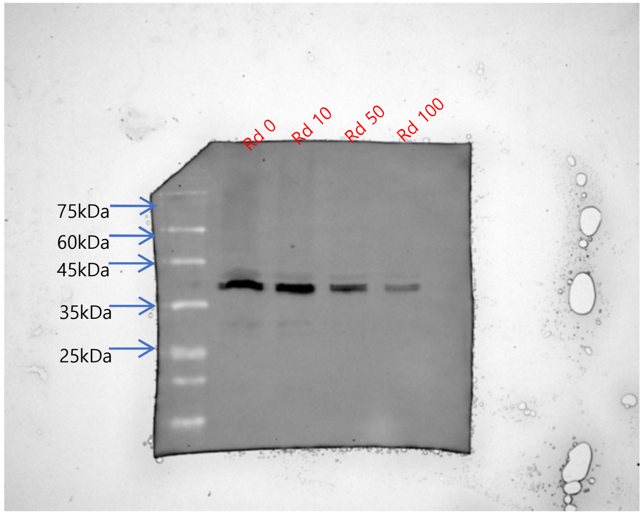

IM-9 20181228 c-myc(CST-5605) 1:500 dilution with 5% skim milk in PBST
2nd ab: Rabbit 57,65kda 12%

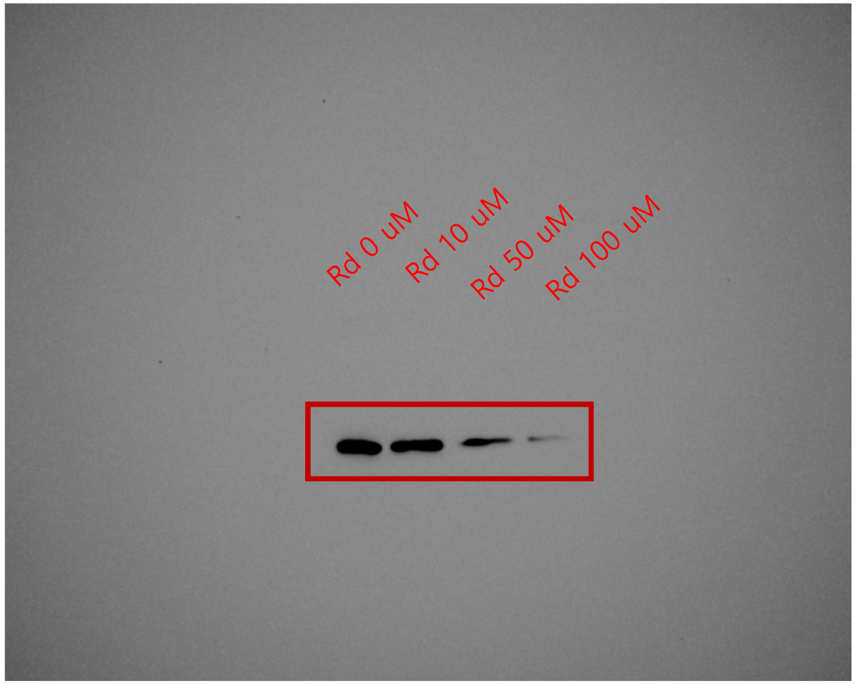

IM-9 20210126 CyclinD1(CST-2978) 1:500 dilution with 5% skim milk in PBST
2nd ab: Mouse 31kda 12%

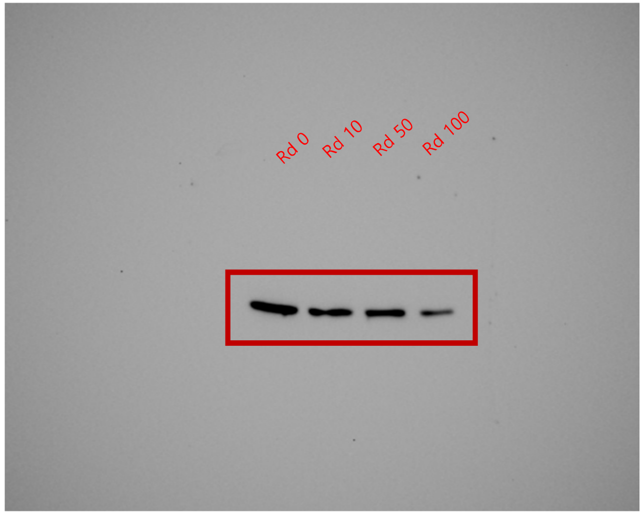

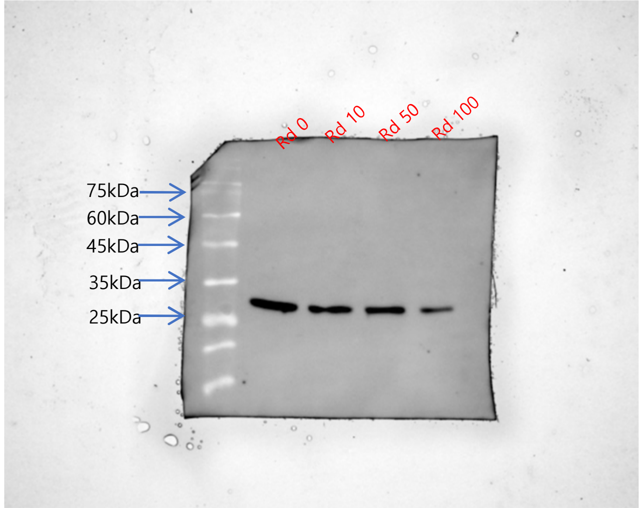

IM-9 20201208 CyclinD3(CST-2936) 1:500 dilution with 5% skim milk in PBST
2nd ab: Mouse

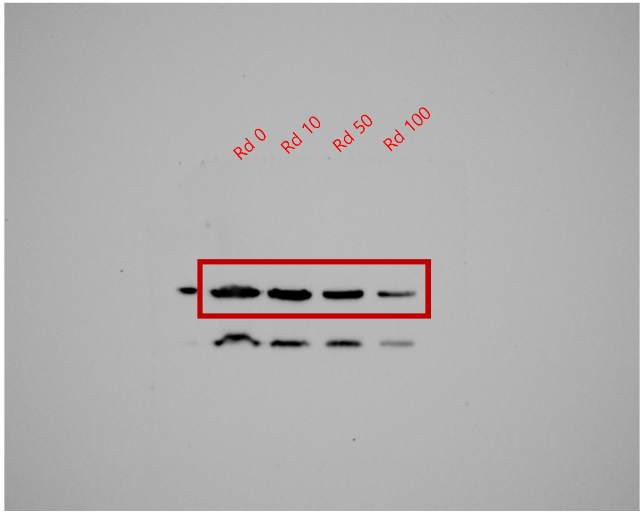

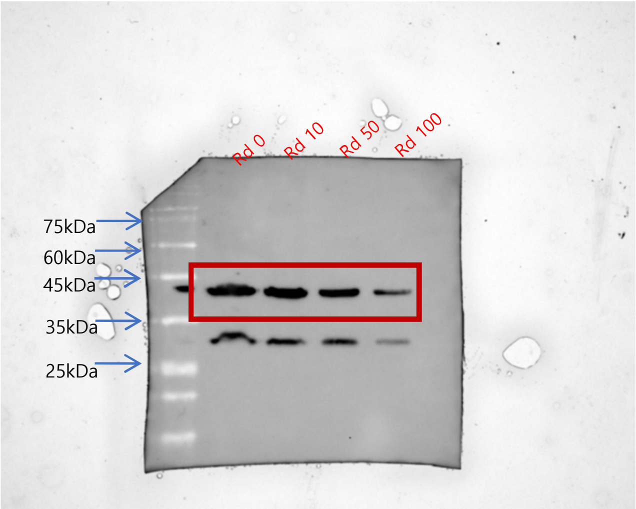

IM-9 20190122 B-actin(SC-47778) 1:200 dilution with 5% skim milk in PBST
2nd ab: Mouse 43kda 12%

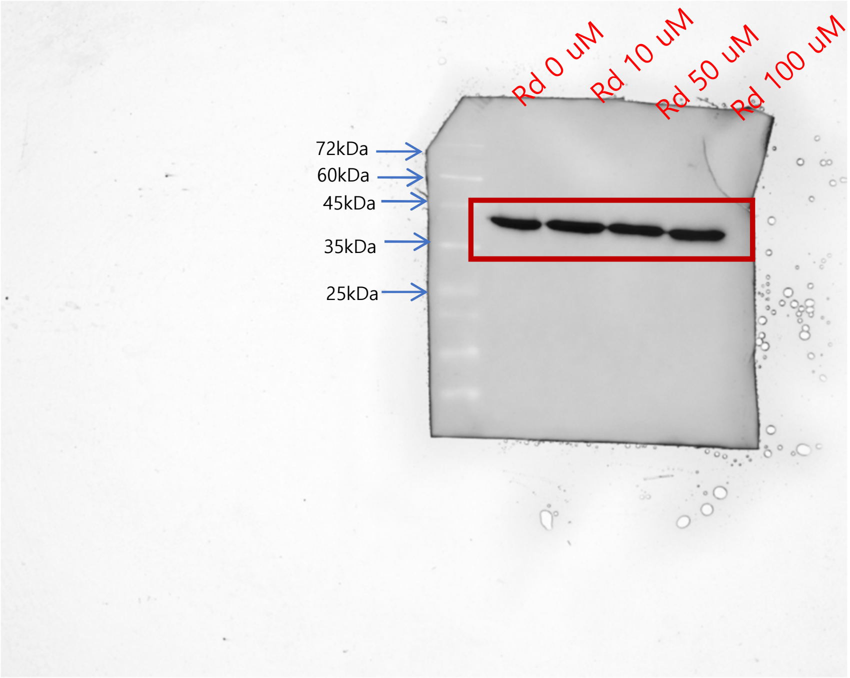

Fig. 3D. Heo et al

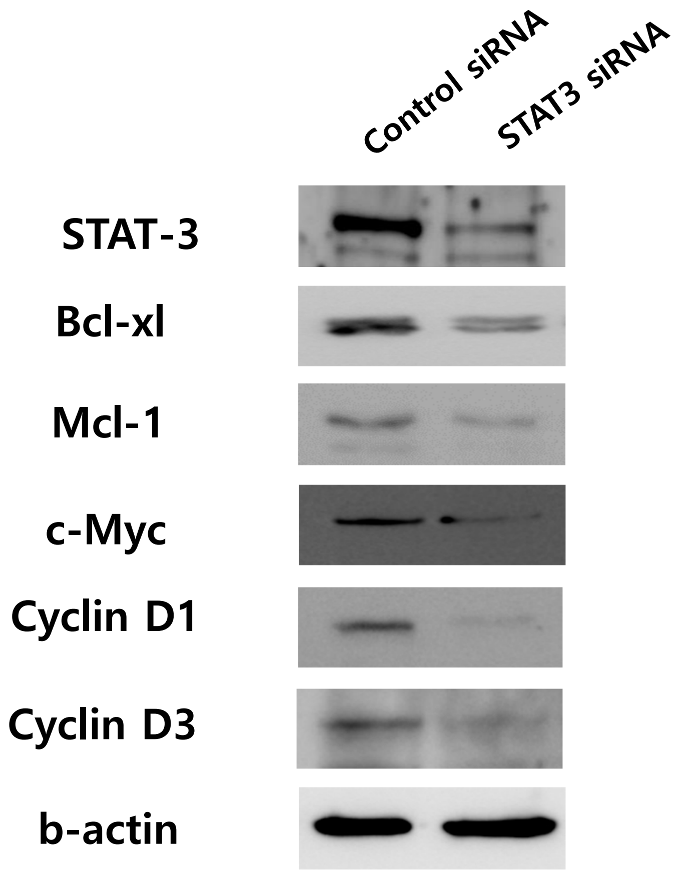

20190925 STAT3(ab-226942) 1:500 dilution with 5% skim milk in PBST 2nd ab: Goat

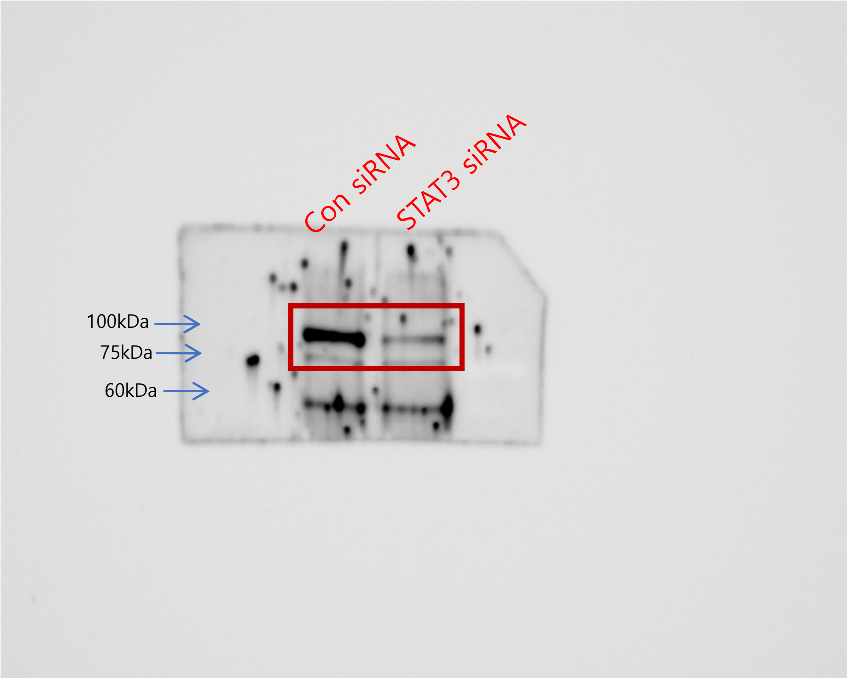

U266B1_siRNA 20190920 c-myc(SC-40) 1:200 dilution with 5% skim milk in PBST
2nd ab: Mouse

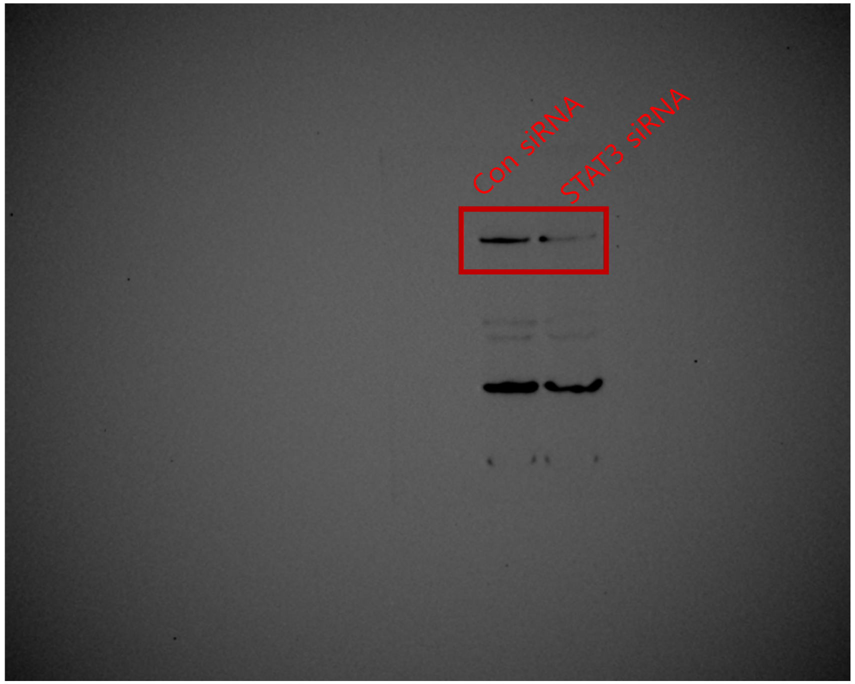

U266B1_siRNA 20190425   BCL-XL(CST-2764) 1:500 dilution with 5% skim milk in PBST
2nd ab: Rabbit

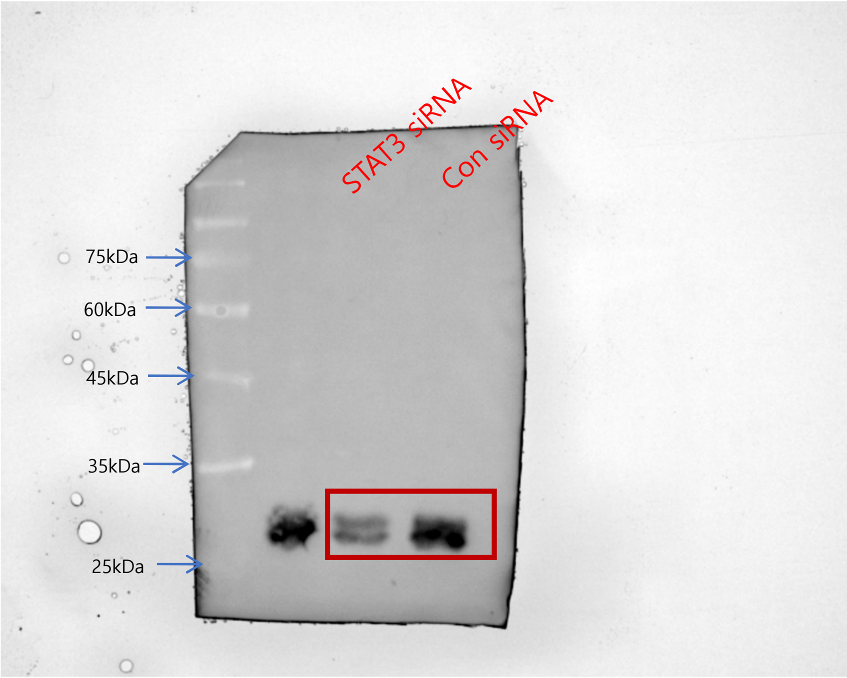

U266B1_siRNA 20190424 MCL-1(CST-5453) 1:500 dilution with 5% skim milk in PBST
2nd ab: Rabbit

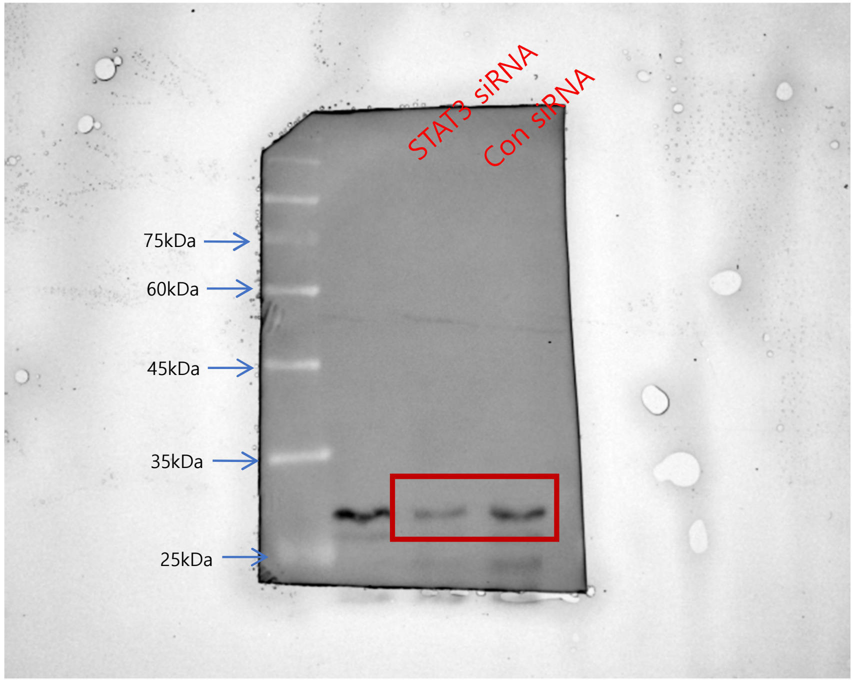

U266B1_siRNA 20190320 Cyclin D1(SC-8396) 1:200 dilution with 5% skim milk in PBST
2nd ab: Mouse

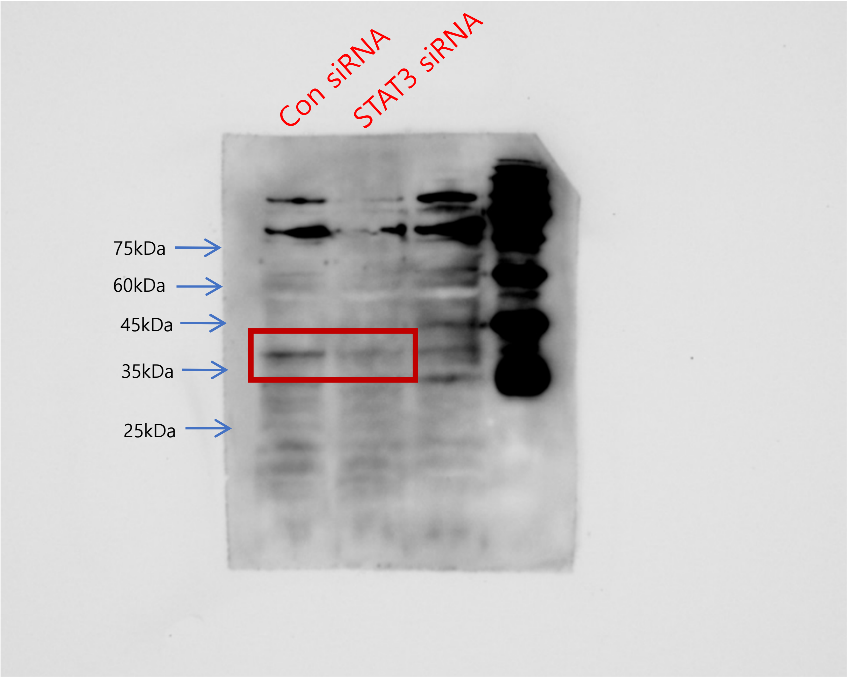

U266B1_siRNA 20190424 Cyclin D3(CST-2936) 1:500 dilution with 5% skim milk in PBST
2nd ab: Mouse

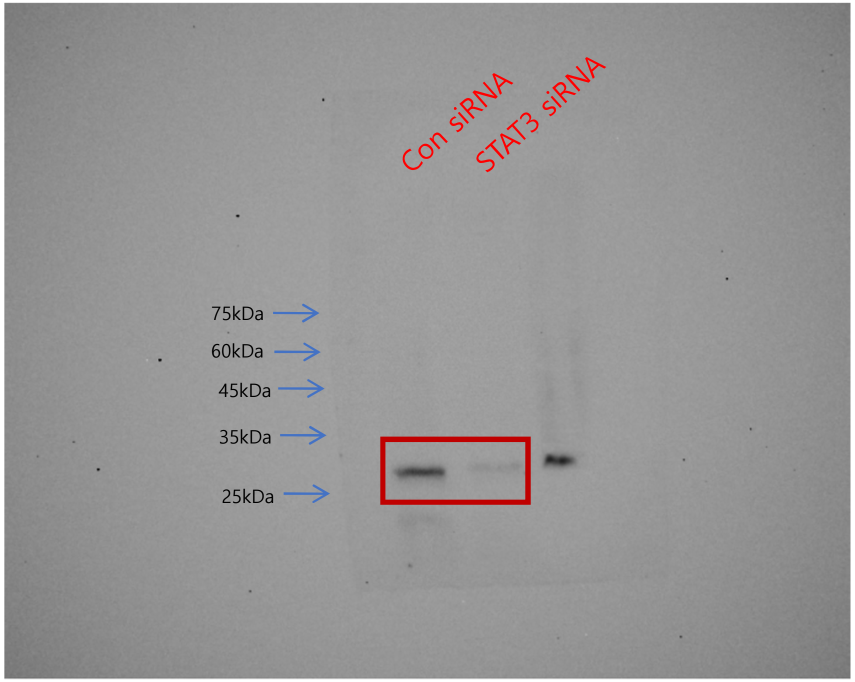

U266B1_siRNA 20190319 β-actin(SC-47778) 1:200 dilution with 5% skim milk in PBST
2nd ab: Mouse

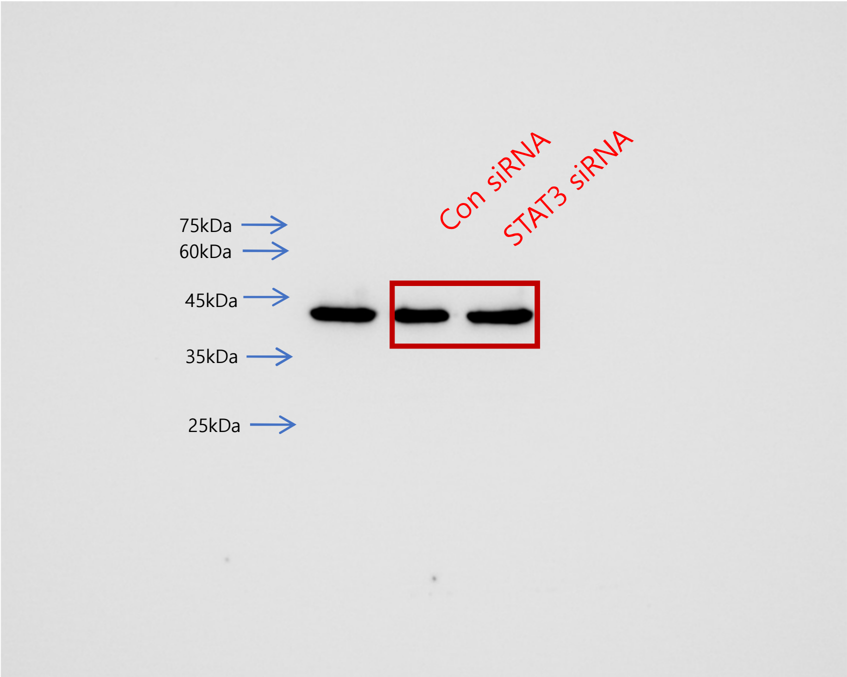

Supplement: S2 Raw images — (PDF) [file pone.0265958.s005.pdf]
