# Supplementary material for: Radotinib inhibits multiple myeloma cell proliferation via suppression of STAT3 signaling
Source: PLoS One. 2022 May 3;17(5):e0265958. doi: 10.1371/journal.pone.0265958 (PMC9064077; doi:10.1371/journal.pone.0265958)

Fig. 4D. Heo et al

IP(STAT3 = SC- 8019, Mouse)

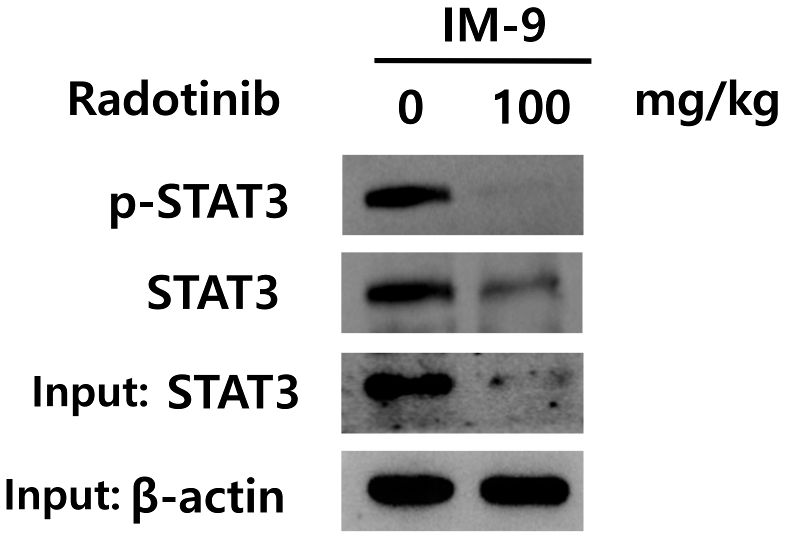

20201126 P-STAT3 (SC-8059) 1:200 dilution with 5% skim milk in PBST 2nd ab: Mouse

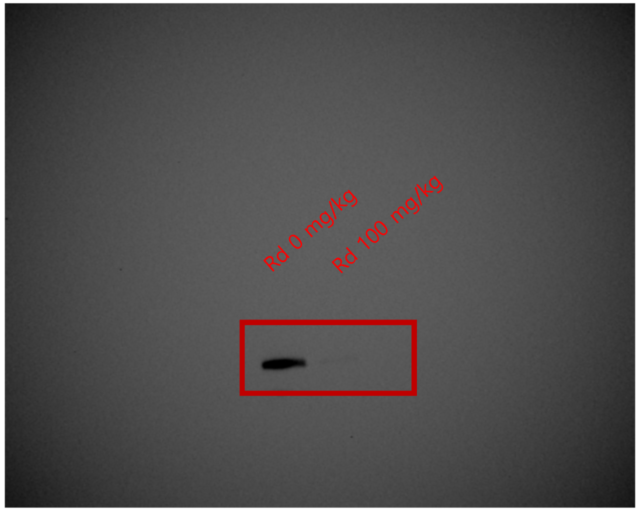

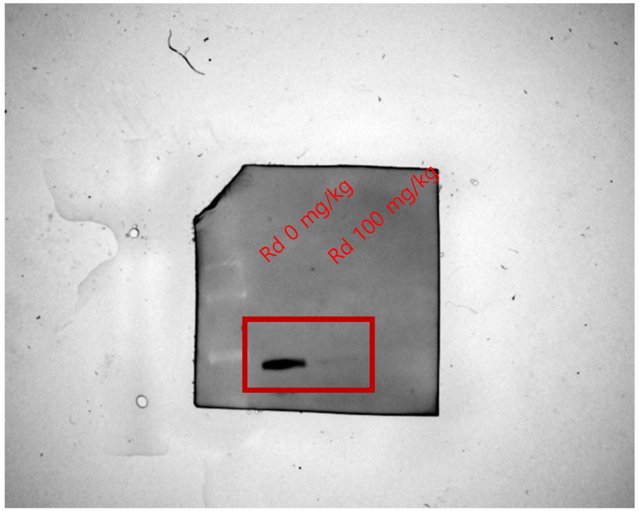

20201201 STAT3 (SC-8019) 1:200 dilution with 5% skim milk in PBST 2nd ab: Mouse

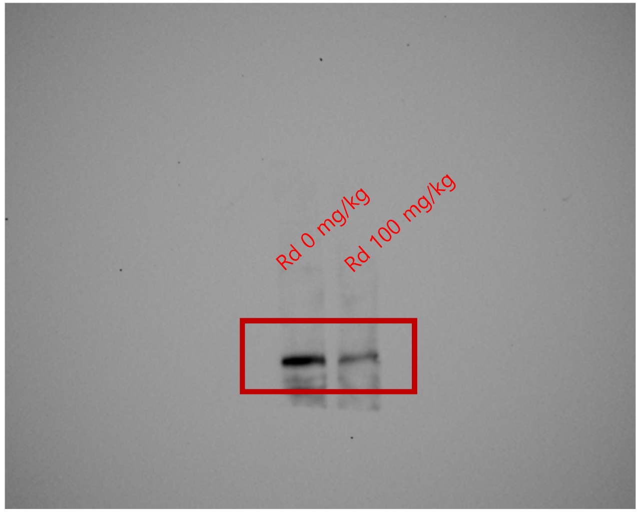

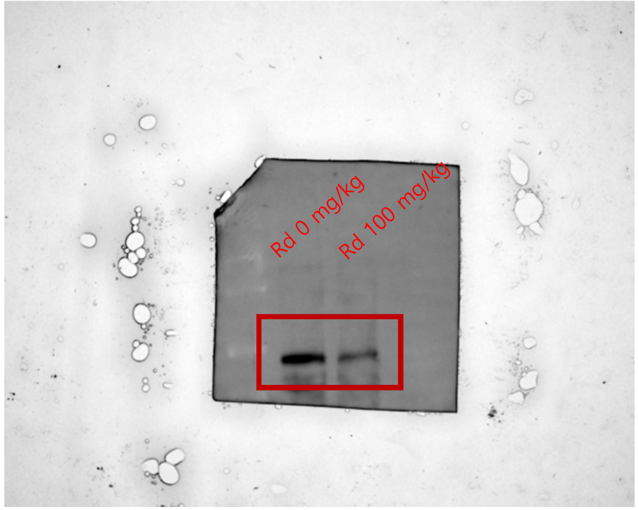

20210113 STAT3 (Input, SC-8019) 1:200 dilution with 5% skim milk in PBST
2nd ab: Mouse

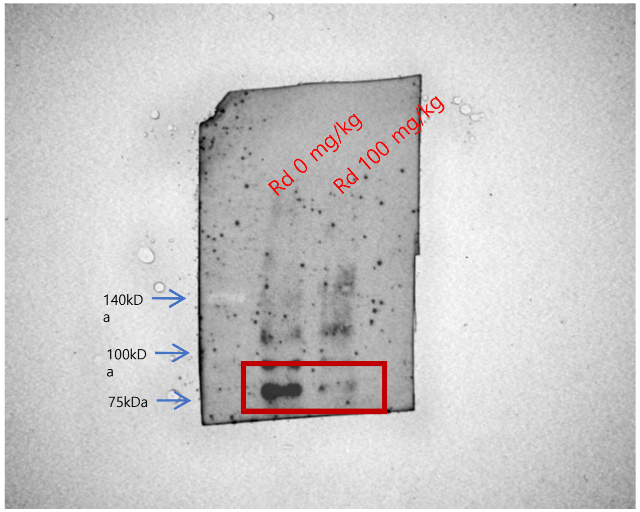

20201126 β-actin (Input, SC-47778) 1:200 dilution with 5% skim milk in PBST 2nd ab: Mouse

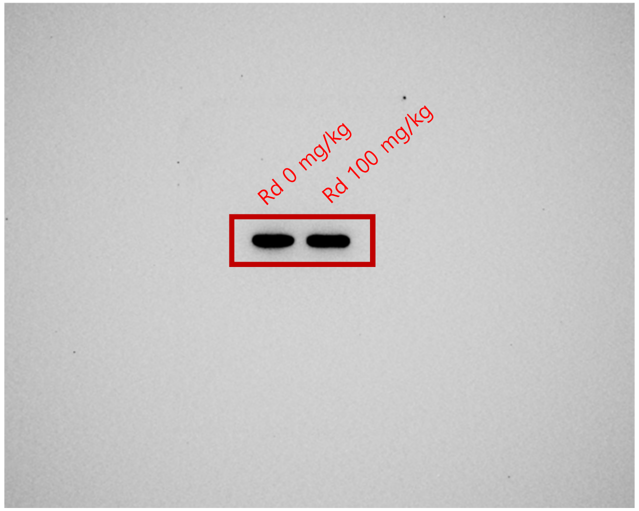

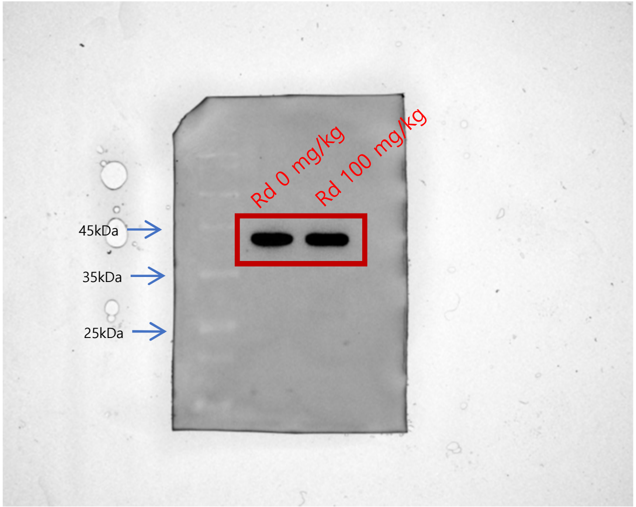

Fig. 4E. Heo et al

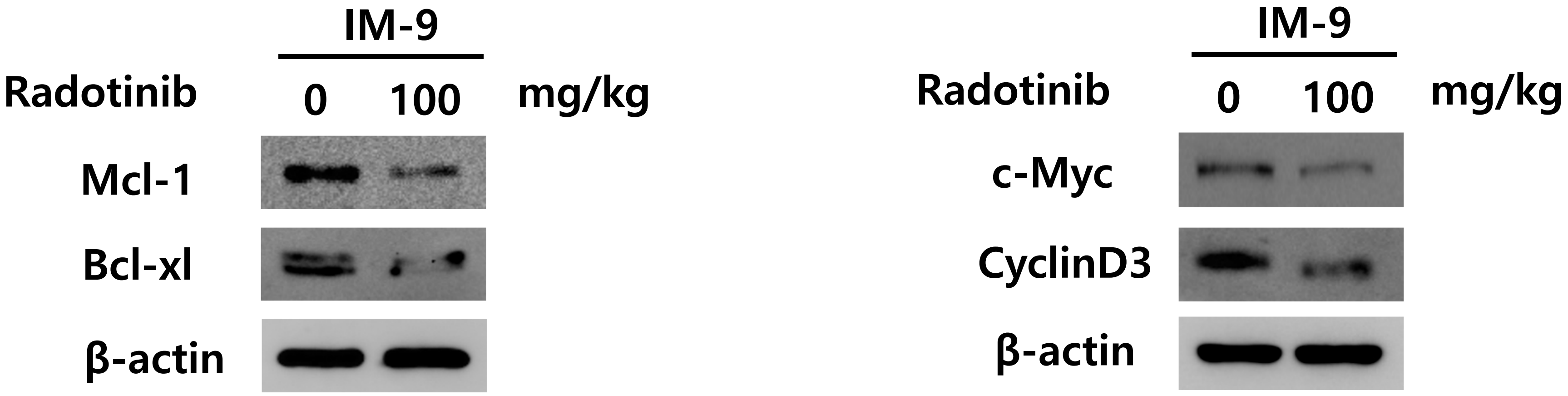

20201124 Mcl-1 (CST-5453) 1:500 dilution with 5% skim milk in PBST 2nd ab: Rabbit

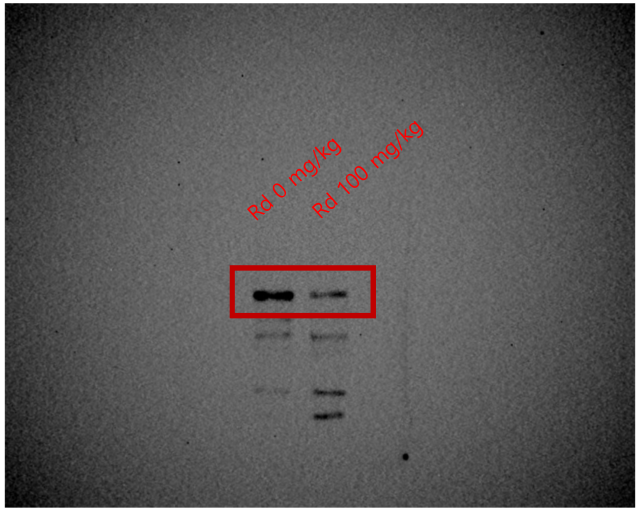

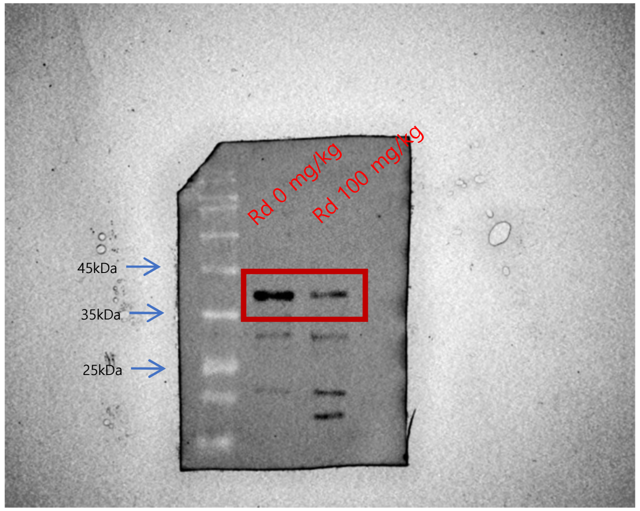

20201126 Bcl-xl (SC-8392) 1:200 dilution with 5% skim milk in PBST 2nd ab: Mouse

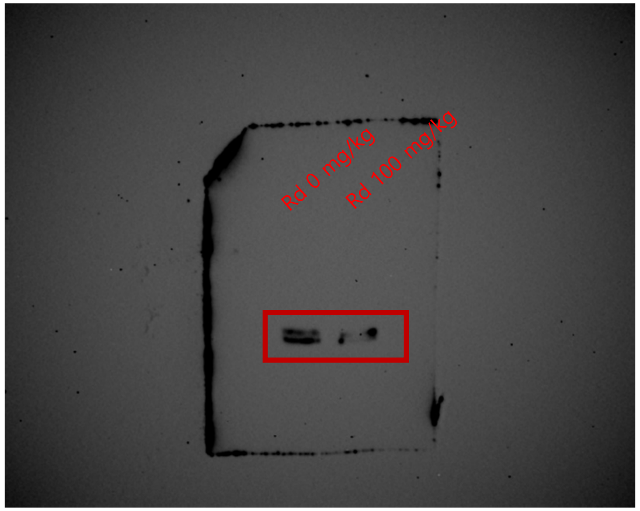

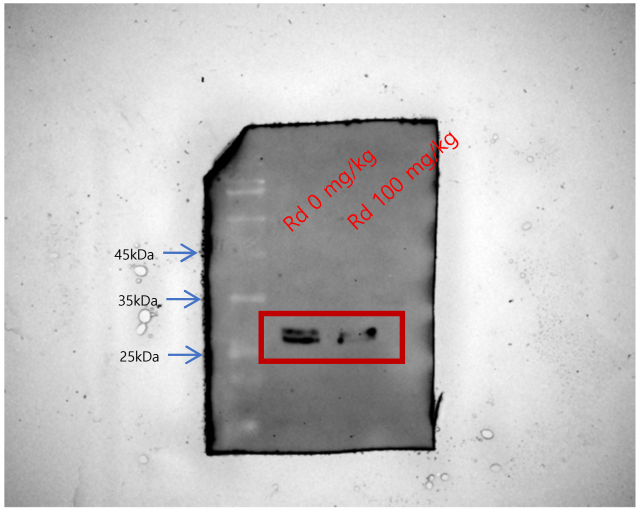

20210219 c-myc(SC-40) 1:200 dilution with 5% skim milk in PBST 2nd ab: Mouse

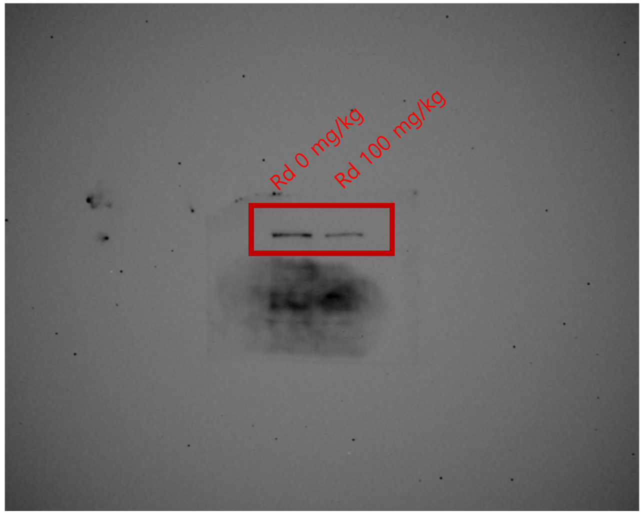

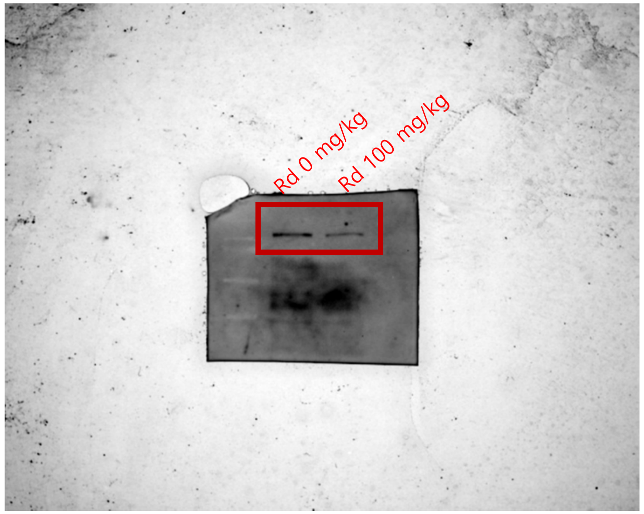

20210108 CyclinD3 (CST-2936) 1:500 dilution with 5% skim milk in PBST 2nd ab: Mouse

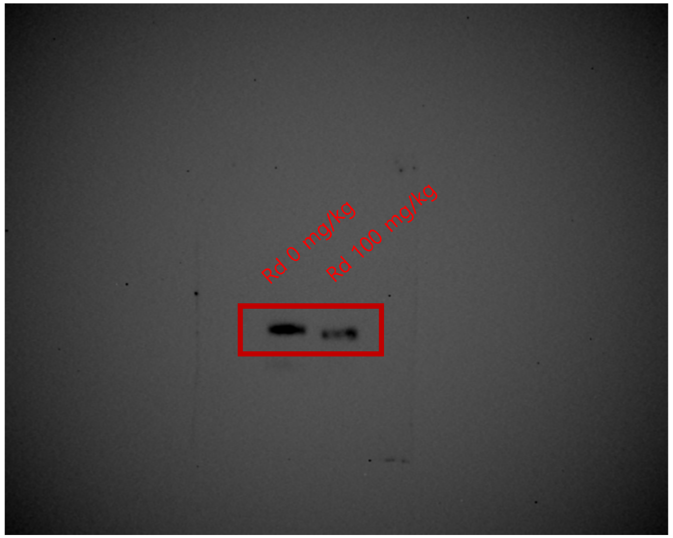

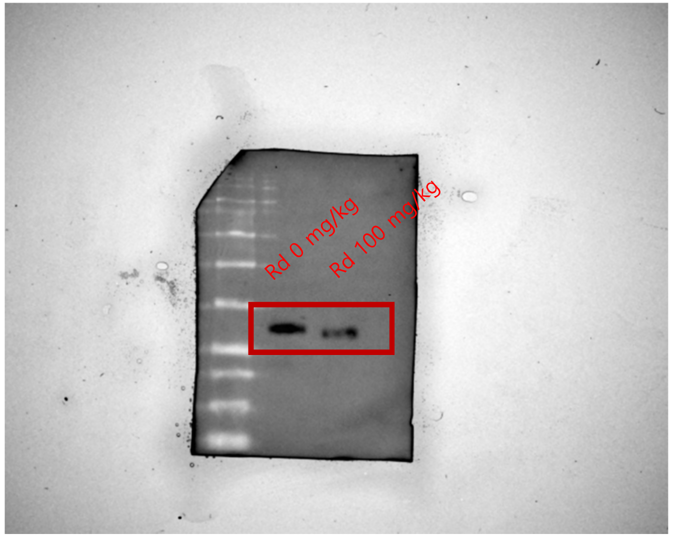

20201201 β-actin (SC-47778) 1:200 dilution with 5% skim milk in PBST 2nd ab: Mouse

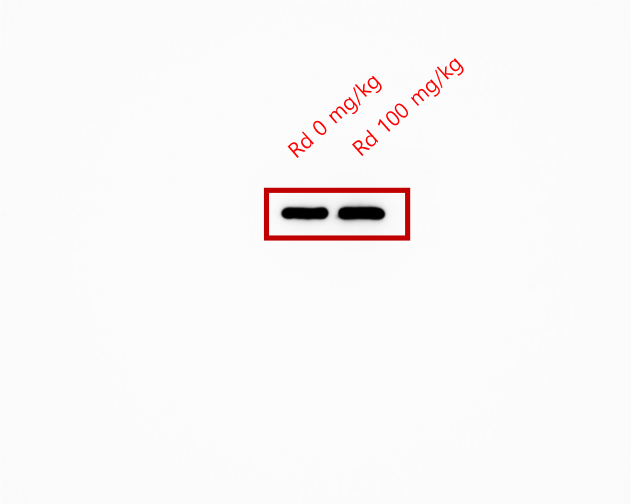

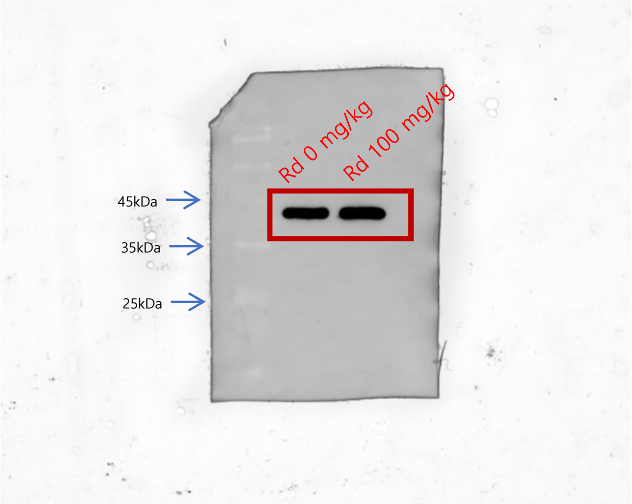

20210224 β-actin(SC-47778) 1:200 dilution with 5% skim milk in PBST 2nd ab: Mouse

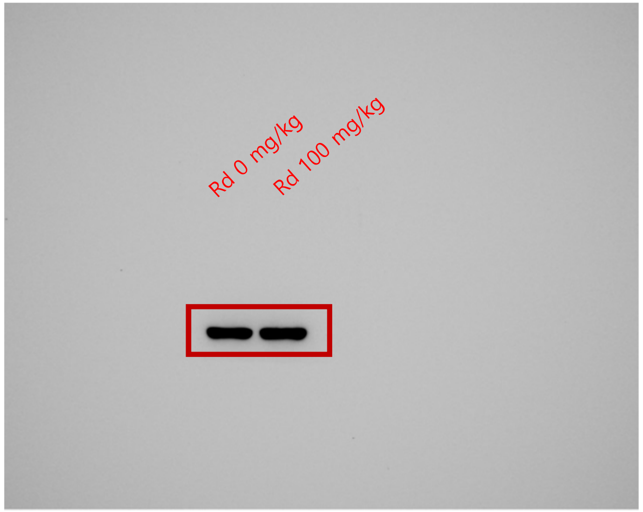

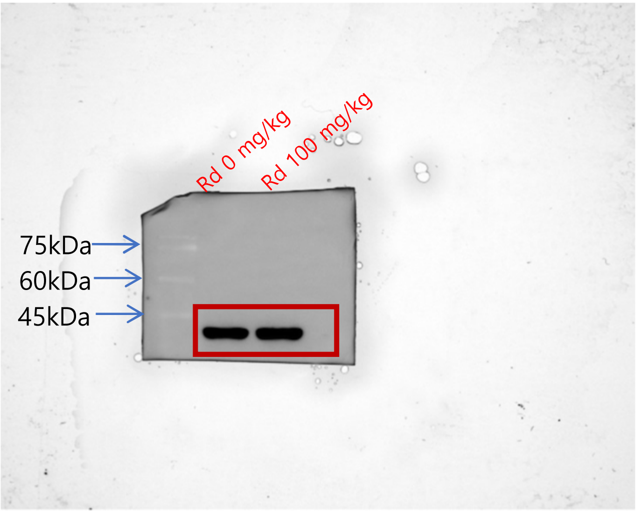

Supplement: S3 Raw images — (PDF) [file pone.0265958.s006.pdf]
